# When Does Vapor Pressure Deficit Drive or Reduce Evapotranspiration?

**DOI:** 10.1029/2019MS001790

**Published:** 2019-10-28

**Authors:** Adam Massmann, Pierre Gentine, Changjie Lin

**Affiliations:** ^1^ Department of Earth and Environmental Engineering Columbia University New York NY USA; ^2^ State Key Laboratory of Hydroscience and Engineering, Department of Hydraulic Engineering Tsinghua University Beijing China

**Keywords:** evapotranspiration, vapor pressure deficit, ecohydrology, stomatal conductance, ecosystem modeling, land‐atmosphere interaction

## Abstract

Increasing vapor pressure deficit (VPD) increases atmospheric demand for water. While increased evapotranspiration (ET) in response to increased atmospheric demand seems intuitive, plants are capable of reducing ET in response to increased VPD by closing their stomata. We examine which effect dominates the response to increasing VPD: atmospheric demand and increases in ET or plant response (stomata closure) and decreases in ET. We use Penman‐Monteith, combined with semiempirical optimal stomatal regulation theory and underlying water use efficiency, to develop a theoretical framework for assessing ET response to VPD. The theory suggests that depending on the environment and plant characteristics, ET response to increasing VPD can vary from strongly decreasing to increasing, highlighting the diversity of plant water regulation strategies. The ET response varies due to (1) climate, with tropical and temperate climates more likely to exhibit a positive ET response to increasing VPD than boreal and arctic climates; (2) photosynthesis strategy, with C3 plants more likely to exhibit a positive ET response than C4 plants; and (3) plant type, with crops more likely to exhibit a positive ET response, and shrubs and gymniosperm trees more likely to exhibit a negative ET response. These results, derived from previous literature connecting plant parameters to plant and climate characteristics, highlight the utility of our simplified framework for understanding complex land‐atmosphere systems in terms of idealized scenarios in which ET responds to VPD only. This response is otherwise challenging to assess in an environment where many processes coevolve together.

## Key Points

- We derive a simplified analytical model for ecosystem‐scale evapotranspiration response to changes in vapor pressure deficit
- Ecosystems exhibit a range of behavior, from reductions to increases in evapotransipration, in response to increasing vapor pressure deficit
- The choice of stomatal conductance model fundamentally alters the relationship between evapotranspiration and vapor pressure deficit

## 1 Introduction

Vapor pressure deficit (VPD) is expected to rise over continents in the future due to the combination of increased temperature and, depending on region, decreased relative humidity (Byrne & O'Gorman, 2013). Increases in VPD increase the atmospheric demand for evapotranspirated water (Monteith, 1965; Penman, 1948) but also reduce stomatal conductance through stomatal closure (Damour et al., 2010; Leuning, 1990; Medlyn et al., 2011; Mott, 2007; Rawson et al., 1977). Understanding the net evapotranspiration (ET) response to these two opposing effects of changes in VPD is crucial for assessing the impact of environmental VPD perturbations on the water cycle.

The opposing effects of increased atmospheric demand and higher stomatal closure lead to two possible perspectives for how ET responds to shifts in VPD. The first, a hydrometeorological perspective, is that higher VPD increases atmospheric demand for water from the land surface, and this drives an increase in ET (Penman, 1948). However, plants' stomata have evolved to optimally regulate the exchange of water and carbon and tend to partially close in response to increased atmospheric dryness (Ball et al., 1987; Farquhar, 1978; Katul et al., 2009; Leuning, 1990; Medlyn et al., 2011). This leads to a plant physiology perspective, in which an increase in VPD may actually correspond to a decrease in ET because of stomatal closure (e.g., Rigden & Salvucci, 2017). In other words, the question “When does VPD drive or reduce ET?” can be related to whether plant regulation or atmospheric demand dominates the ET response.

The ET response to changes in VPD alters water partitioning between the soil and atmosphere. If ecosystem plant response reduces ET with atmospheric drying, then soil moisture will be better conserved. This represents a sensible evolutionary strategy to cope with aridity: save water for periods when atmospheric demand for water is relatively low, and atmospheric carbon can be accessed with a relatively smaller cost in water loss. If instead stomata were fully passive (similar to soil pores; e.g., Or et al., 2013), increased atmospheric aridity would strongly reduce soil moisture (Berg et al., 2017). This could further increase aridity as low soil moisture levels increase the Bowen ratio, leading to increased temperature and atmospheric drying (Berg et al., 2016; Bouchet, 1963; Brutsaert, 1999; Gentine et al., 2016; Morton, 1965; Ozdogan et al., 2006; Salvucci & Gentine, 2013; Zhou et al., 2019). Therefore, passive regulation and a lack of soil moisture conservation do not seem to be a sensible strategy for plants from an evolutionary standpoint. This simplified logic explains generally why plants evolved to respond to VPD but also excludes many details and special cases (e.g., plant to plant interaction, thermal regulation with transpiration, and highly specialized photosynthesis strategies like Crassulacean acid metabolism photosynthesis; see Brooker, 2006; Cushman, 2001; Lin et al., 2017).

We can use intuition about plant water conservation strategy to hypothesize about ET response to changes in VPD. Plants and ecosystems that evolved to conserve water, such as arid shrubs, should be more likely to reduce ET with increasing VPD, and plants that have evolved or have been engineered to prioritize carbon gain over water conservation, such as crops, will be more likely to increase ET with increasing VPD. Atmospheric conditions must matter as well. At the ecosystem scale, there are limits to plant water conservation strategies. As atmospheric demand for water (VPD) increases, ecosystems may begin to reach their water conservation limits and might not be able to entirely limit ET flux to the atmosphere. At this stage, any further increase in VPD will most likely drive a (limited) increase in ET, because the increase in atmospheric demand for water overwhelms the limited plant response to conserve water.

The objective of the present manuscript is to use reasonable approximations established in prior research as a tool to develop a framework for understanding plant responses to atmospheric drying and the VPD dependence of ET while keeping other variables fixed. This framework will aid interpretation of observations and full complexity models and facilitate the disentanglement of complex land‐atmosphere feedbacks. In particular, our approach has applications for understanding climate change impacts, given expected increases in VPD with rising temperature. In the past, similar simplified approaches were used to understand interactions between stomatal conductance, ET, and the environment (e.g., Jarvis, 1984; Jarvis & McNaughton, 1986; McNaughton & Jarvis, 1991). However, these researchers did not explore explicitly the sensitivity of ET to VPD, including VPD's effect on stomatal conductance and plant function.

Approaching the problem of ET response to VPD is aided by recent results drastically improving our understanding of VPD's impact on physiology, especially at the leaf level. Medlyn et al. (2011) developed a model for leaf‐scale stomatal conductance (*g*_*s*_), including VPD response, by combining an optimal photosynthesis theory (Cowan & Farquhar, 1977; Katul et al., 2009; Katul et al., 2010) with an empirical approach and extended the use of this model to the ecosystem scale in Medlyn et al. (2017). Additionally, Zhou et al. (2014) demonstrated that a quantity underlying water use efficiency $\left(uWUE=\frac{GPP\;\sqrt{VPD}}{ET}\right)$ properly captures a constant relationship between gross primary production (GPP), ET, and VPD over a diurnal cycle at the ecosystem scale. *uWUE* is also relatively well conserved in the growing season across space and time, within a plant functional type (Zhou et al., 2015). While stomatal conductance parameterizations and *uWUE* greatly simplify complex plant physiological processes, they still capture ecosystem behavior for vegetated surfaces (Medlyn et al., 2017; Zhou et al., 2014) and are useful tools to transparently develop intuition for the behavior of complex land‐atmosphere systems.

In this manuscript, we leverage *uWUE* and recent developments in stomatal conductance parameterizations (Medlyn et al., 2011) with a Penman‐Monteith framework (hereafter PM; Monteith, 1965; Penman, 1948) to derive the theoretical one‐way response of ET to VPD with other environmental variables properly controlled for, that is, we develop a framework for evaluating the partial derivative of ET with respect to VPD. It is useful to disconnect the impact of VPD from other variables as VPD is known to increase dramatically with future climate and limit ET more than soil moisture (Novick et al., 2016), but disentangling VPD effects from soil moisture effects has been difficult in previous research, given their covariability (Lin et al., 2018; Zhou et al., 2019). We are able to disentangle the effect of VPD on ET because we explicitly include VPD's full effect on stomatal conductance, including its impact on photosynthesis. The use of an energy balance framework (PM) allows us to include in our analysis the effects of the energy cost of evaporating water from a surface, which is an important factor in the natural environment, compared to prescribed in situ environmental conditions. This is relevant because previous research focusing on the leaf scale (Damour et al., 2010; Mott & Peak, 2013; Oren et al., 1999; Rawson et al., 1977; Turner et al., 1984) does not consistently or analogously include the energetic constraints on ET under which ecosystems operate. The leaf‐scale results agree that stomatal conductance decreases in response to VPD (Damour et al., 2010; Oren et al., 1999), which we expect to be true for an ecosystem as well. However, leaf‐scale results also indicate that transpiration usually increases with increasing VPD in a concave downward shape (e.g., Mott & Peak, 2013; Rawson et al., 1977; Turner et al., 1984), which may not be true for ET at the ecosystem scale once energetic constraints on ET are included in the analysis. Our approach allows us to estimate the expected ET response to VPD at the ecosystem scale, including the effects of surface energy balance constraints, and assess how ecosystem ET response to VPD deviates from previous leaf‐scale analyses.

This manuscript presents the range of possible ecosystem‐scale ET responses to VPD, given parameters previously established in peer‐reviewed literature. Additionally, we explore the sensitivity of the ET‐VPD relationship to stomatal model and framework choice, highlighting the importance of (1) future research on stomatal conductance and ecosystem‐scale modeling and (2) thoughtful selection of photosynthesis and stomatal conductance models in more sophisticated land surface and Earth system models.

## 2 Methods

The PM equation (Monteith, 1965; Penman, 1948) estimates ET as a function of observable atmospheric variables and surface conductances:

(1) $$ET=\frac{\Delta R_{\text{net}}+g_{a}\rho_{a}c_{p}VPD}{\Delta +\gamma (1+\frac{g_{a}}{g_{s}})},$$

where Δ is the change in saturation vapor pressure with temperature, given by Clausius‐Clapeyron ( $\frac{d\;e_{s}}{d\;T}$), *R*_net_ is the net radiation minus ground heat flux, *g*_a_ is aerodynamic conductance, *ρ*_a_ is air density, *c*_p_ is specific heat of air at constant pressure, *γ* is the psychometric constant, and *g*_s_ is the stomatal conductance (Table 1). The issue with PM is that *g*_s_ is a function of carbon uptake, which has a strong functional relation to ET through stomatal function. Therefore, when PM is formulated in terms of *g*_s_, it is an implicit function of ET itself rather than an explicit function of ET. Here we will derive a new form of PM in which ET is an explicit function of plant parameters and environmental conditions and use it to assess the ecosystem‐scale response to VPD (by taking a partial derivative).

**Table 1:** Definition of Symbols and Variables, With Citation for How Values Are Calculated, if Applicable

Variable	Description	Units	Citation
*e*_s_	Saturation vapor pressure	Pa	—
*T*	Temperature	K	—
*P*	Pressure	Pa	—
Δ	$\frac{de_{s}}{dT}$	Pa/K	—
*R*_net_	Net radiation at land surface minus ground heat flux	W/m^2^	—
*g*_a_	Aerodynamic conductance	m/s	Shuttleworth (2012)
*ρ*_a_	Air density	kg/m^3^	—
*c*_p_	Specific heat capacity of air at constant pressure	J·K^−1^·kg^−1^	—
*VPD*	Vapor pressure deficit	Pa	—
*γ*	Psychometric constant	Pa/K	—
*g*_s_	Atomatal conductance	m/s	Medlyn et al. (2017)
*g*_1_	Ecosystem‐scale slope parameter	Pa^0.5^	Medlyn et al. (2017)
*R*	Universal gas constant	J·mol^−1^·K^−1^	—
*R*_air_	Gas constant of air	J·K^−1^·kg^−1^	—
*σ*	Uncertainty parameter	—	—
*c*_a_	CO_2_ concentration	*μ*mol CO_2_/mol air	—
$\lambda =\frac{\partial \;transpiration}{\partial \;CO_{2}\;assimilation}$	Marginal water cost of leaf carbon	mol H_2_O/mol CO_2_	—
Γ	CO_2_ compensation point	—	—
Γ^*^	CO_2_ compensation point without dark respiration	—	—
*GPP*	Gross primary production	*μ*mol C·s^−1^ m^−2^	—
*ET*	Evapotranspiration	W/m^2^	—
*uWUE*	Underlying water use efficiency	*μ*mol C Pa^0.5^ J^−1^ ET	Zhou et al. (2015)

Medlyn et al. (2011) developed a model for stomatal conductance (*g*_s_) by combining an optimal photosynthesis theory (Cowan & Farquhar, 1977) with an empirical approach, which describes the dependence of *g*_s_ to VPD. They also extended this model to the ecosystem scale (Medlyn et al., 2017):

(2) $$g_{s}=\frac{R\;T}{P}\;1.6\left(1+\frac{g_{1}}{\sqrt{VPD}}\right)\frac{GPP}{c_{a}},$$

where *g*_1_ is a VPD “slope” parameter, GPP is the ecosystem‐scale GPP, and *c*_a_ is the atmospheric CO_2_ concentration at the canopy. Medlyn et al. (2011) relate the slope parameter (*g*_1_) to physical parameters as

(3) $$g_{1}\propto \sqrt{\frac{3\;\Gamma^{*}\;\lambda }{1.6}},$$

where g_1_ is presented in units of $\sqrt{\text{mol fraction}}$ for convenience (which can be converted to $\sqrt{Pa}$ using the ideal gas law and ambient pressure), Γ^*^ is the CO_2_ compensation point for photosynthesis (without dark respiration), and *λ* is the marginal water cost of leaf carbon ( $\frac{\partial \;\text{transpiration}}{\partial \;CO_{2}assimilation}$; Farquhar et al., 1980; Katul et al., 2009). So *g*_1_ is a leaf‐scale term reflecting the trade‐off of water for carbon uptake. The higher the *g*_1_, the more open the stomata and the more they release water in exchange for carbon.

While equation (2) can be used in PM (equation (1)), it will make analytical work with the function intractable because GPP is functionally related to ET itself. Additionally, a perturbation to VPD should induce a physiological plant response that will alter GPP and cause an indirect change in stomatal conductance, in addition to the direct effect of VPD in equation (2). Therefore, in order to derive the full plant response of ET to VPD, we must account for the functional relationship between GPP, ET, and VPD and its effect on stomatal conductance. We can use aforementioned semiempirical results of Zhou et al. (2015), which were inspired by optimal photosynthesis theory, as a tool to approach this problem. Zhou et al. (2015) showed that underlying Water Use Efficiency (*uWUE*)

(4) $$uWUE=\frac{GPP\cdot \sqrt{VPD}}{ET}$$

is relatively constant across time within a plant functional type and correctly captures a constant relationship between GPP, ET, and VPD over a diurnal cycle during the growing season (Zhou et al., 2014). The theoretical derivation of the square root VPD dependence in *uWUE* leverages the same assumptions used in Medlyn et al. (2011) to derive the square root VPD dependence of the stomatal conductance model (equation (2)). We can use *uWUE* to remove the *GPP* dependence in *g*_s_ in a way that makes PM analytically tractable:

(5) $$g_{s}=\frac{R\;T}{P}1.6\left(1+\frac{g_{1}}{\sqrt{VPD}}\right)\frac{uWUE\;ET}{c_{a}\;\sqrt{VPD}}.$$

Plugging equation (5) into equation (1) and rearranging gives a new explicit expression for PM, in which dependence on *GPP* is removed:

(6) $$ET=\frac{\Delta R_{\text{net}}+\frac{g_{a}\;P}{T}\left(\frac{c_{p}VPD}{R_{\text{air}}}-\frac{\gamma c_{a}\sqrt{VPD}}{R\;1.6\;uWUE(1+\frac{g_{1}}{\sqrt{VPD}})}\right)}{\Delta +\gamma }.$$

By accounting for photosynthesis changes in ecosystem conductance, with equation (6), we have used recent results (Medlyn et al., 2011, 2017; Zhou et al., 2014, 2015) to derive ET explicitly as function of environmental variables and two plant‐specific constants, the slope parameter (*g*_1_), and *uWUE*. For the first time, we have removed the implicit dependence of ET on itself through the stomatal conductance term, and we have also replaced the added complexity of a stomatal conductance reduction factor and a photosynthesis model with a single parameter (*uWUE*). Both the plant parameters reflect water conservation strategy. The slope parameter is related to the willingness of stomata to trade water for CO_2_ and to keep stomata open (carbon cost in terms of water). *uWUE* is a semiempirical ecosystem‐scale constant related to how WUE changes with VPD (specifically *VPD* ^−1/2^). It is also roughly proportional to physical constants:

$$uWUE\underset{∼}{\propto }\sqrt{\frac{c_{a}-\Gamma }{1.6\lambda }},$$

where Γ is the CO_2_ compensation point (equation (5) in Zhou et al., 2014). So *uWUE* is related to atmospheric CO_2_ concentration and compensation point and is inversely proportional to the marginal water cost of leaf carbon (*λ*). This relationship with *λ* (∝*λ* ^−1/2^) is important as it is the inverse of g_1_'s relationship with *λ* ( $\propto \sqrt{\lambda }$). So *uWUE* should increase as *λ* decreases, and g_1_ should decrease as *λ* decreases.

With equation (6), we can take the partial derivative of ET with respect to VPD to understand whether VPD drives or reduces ecosystem‐scale ET:

(7) $$\frac{\partial \;ET}{\partial \;VPD}=\frac{2\;g_{a}\;P}{T(\Delta +\gamma )}\left(\frac{c_{p}}{R_{\text{air}}}-\frac{\gamma c_{a}}{1.6\;R\;\;uWUE}\left(\frac{2g_{1}+\sqrt{VPD}}{2{\left(g_{1}+\sqrt{VPD}\right)}^{2}}\right)\right),$$

providing analytical framework for ecosystem response to atmospheric demand with environmental conditions held fixed. There are a few subtleties to taking the derivative in equation (7): Δ ( $\frac{de_{s}}{dT}$) and *VPD* are functionally related, so while taking the derivative, we evaluate $\frac{\partial \;ET}{\partial \;VPD}=\frac{\partial \;ET}{\partial \;e_{s}}{\left.\frac{\partial \;e_{s}}{\partial \;VPD}\right|}_{\text{RH fixed}}+\frac{\partial \;ET}{\partial \;RH}{\left.\frac{\partial \;RH}{\partial \;VPD}\right|}_{e_{sfixed}}$. *RH* and *e*_*s*_ are assumed to be approximately independent, which is supported by data (see supporting information).

This derivation relied either implicitly or explicitly on several assumptions. First, we assume that VPD at the leaf surface is the same as VPD at measurement height; physically, this implies that leaves are perfectly coupled to the atmosphere. In reality, for some conditions and plant types, the leaves can become decoupled from the boundary layer (De Kauwe et al., 2017; Medlyn et al., 2017). Therefore, our derivation will be most applicable in times like the growing season (when we also expect *uWUE* to be most valid), when relatively high insolation induces instability and convective boundary layers, and we would expect the surface to be generally well coupled. An additional assumption in the formulation of *uWUE* (Zhou et al., 2014, 2015) and the stomatal conductance model of Medlyn et al. (2017) is that direct soil evaporation contributions to ET remain small relative to transpiration. Again, this should be more true during the growing season. The ratio of evaporation to transpiration may increase immediately after rainfall events due to high soil moisture, ponding, and interception, but VPD is generally low anyways during these times. However, some plant types allow for systematically larger contributions of evaporation in ET, particularly those with sparse canopies and smaller relative amounts of transpiration. We therefore might expect that the theory will be most applicable to forest plant functional types, which will be most strongly coupled to the boundary layer due to larger surface roughness, and will also generally have the highest ratios of transpiration to evaporation. Finally, we assume that *g*_1_ and *uWUE* are constant with respect to the conceptual VPD perturbation. Both quantities have been shown to be relatively constant with respect to changes in VPD (Franks et al., 2017; Zhou et al., 2014). These parameters will however vary with plant species and characteristics (e.g., wood density; Lin et al., 2015), as well as environmental conditions including soil water content and temperature (Lin et al., 2015; Manzoni et al., 2013). Exploring possible soil moisture (in)dependence of the plant parameters (*g*_1_ and *uWUE*) is particularly interesting because soil moisture only enters the partial derivative directly through these plant parameters. If the plant parameters are weak functions of soil moisture, then the theory can be directly applied to a broader range of conceptual VPD scenarios, including observed compound events between high VPD and low soil moisture (Zhou et al., 2019). Supporting information for this manuscript explores the soil moisture dependence of the plant parameters, but excessive noise and inconsistencies preclude conclusions about the nature of any dependence. We provide it in case it is of interest to the reader and to motivate future research.

Because equation (7) is a partial derivative, its utility is as a conceptual model for the change in ET in response to VPD with all other variables held fixed. This provides a useful tool for identifying the effect of VPD on ET through atmospheric demand and plant response and allows a practitioner to disentangle complicated feedbacks when many quantities covary. However, users of equation (7) should take care that their interpretation matches the assumptions inherent in a partial derivative. For example, results will only be as valid as the assumption that *g*_1_ and *uWUE* (and by extension *λ*) are fixed with respect to the user's conceptual change in VPD. Care must also be taken with possible indirect effects associated with a change in VPD: For example, a change in ET induced by a change in VPD can cause a change in surface temperature, which would drive a change in net radiation. These types of indirect effects and feedbacks are not considered in equation (7): Temperature (a variable) is mathematically fixed.

### 2.1 Framing Equation (7) With Previous Research

To account for both the variability in plant parameters and the environment, we will systematically analyze how the ET response to VPD (equation (7)) varies. Equation (7) includes a “sign” term that determines the sign of the response in addition to magnitude:

(8) $$\frac{c_{p}}{R_{\text{air}}}-\frac{\gamma c_{a}}{1.6\;R\;\text{uWUE}}\left(\frac{2g_{1}+\sqrt{VPD}}{2{\left(g_{1}+\sqrt{VPD}\right)}^{2}}\right),$$

and a “scaling” term multiplying the sign term:

(9) $$\frac{g_{a}\;P}{T(\Delta +\gamma )}.$$

In the sign term, most of the quantities are relatively constant, except for VPD and the plant parameters *g*_1_ and *uWUE*. In the scaling term, most of the terms are relatively constant with the exception of aerodynamic conductance (*g*_a_) and temperature (especially its effect through Δ). To determine the range of probable ET responses to VPD, we will systematically vary these parameters according to Table 2, while all other parameters are held fixed (Table 3). Physical variables (*g*_a_ and *T*) and plant physiological parameters (*g*_1_ and *uWUE*) are varied according to literature‐based expectations for a range of growing season conditions and plant types (Medlyn et al., 2017; Zhou et al., 2015). Using this previous literature, we can connect the effect of varying plant parameters to specific plant types and characteristics.

**Table 2:** Variable Quantities in the Evapotranspiration Response to Vapor Pressure Deficit

Symbol	Units (units in citation)	Min	Med	Max	Citation
*g*_1_	Pa^1/2^ (kPa^1/2^)	63.25 (2.00)	126.49 (4.00)	189.74 (6.00)	Figures 2 and 7
					(Medlyn et al., 2017)
*uWUE*	*μ*mol C Pa^1/2^ J^−1^ ET	2.33 (6.99)	3.17 (9.52)	4.01 (12.05)	Table 4
	(g C hPa^1/2^ kg^−1^ H_2_O)				(Zhou et al., 2015)
*T*	°C	10.00	20.00	30.00	—
*g*_a_	m/s	0.015	0.035	0.055	—

*Note.* Each value is varied to determine the effect of a range of expected plant and environmental conditions on evapotranspiration response to vapor pressure deficit. A citation is provided for values of *g*_1_ and *uWUE*, which are directly derived from previous literature. Conceptually, min. values are extracted from literature to correspond to approximately the 15th percentile of observed conditions during the growing season, med. values correspond to approximately the 50th percentile, and max. values correspond to approximately the 85th percentile. Values for *T* and *g*_a_ are calculated from FLUXNET‐2015 data (see supporting information for description), rounding the 15th, 50th, and 85th percentiles to the nearest 5°C and 0.005 m/s, respectively.

**Table 3:** Quantities That Are Fixed in the Evapotranspiration Response to Vapor Pressure Deficit (Relative to Those in Table 2)

Symbol	Units	Value
*P*	Pa	100000.00
*γ*	Pa/K	64.50
*R*_air_	J·K^−1^·kg^−1^	288.00
*c*_a_	*μ*mol CO_2_/mol air	400.00

All code and data used in this analysis, including those used to generate the figures and tables, are publicly available online (https://github.com/massma/climate_et).

## 3 Results

**Figure 1:**
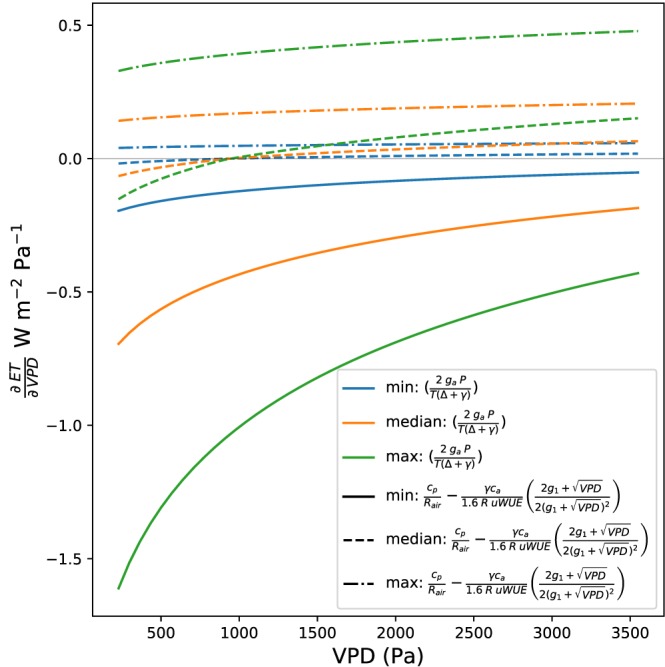
The functional form of $\frac{\partial \;ET}{\partial \;VPD}$ for minimum, median, and maximum values of both the sign term and the scaling term. ET = evapotranspiration; uWUE = underlying Water Use Efficiency; VPD = vapor pressure deficit.

By varying four parameters (*g*_a_, *T*, *uWUE*, and *g*_1_) at three different values (Table 2), we generate nine different values for the scaling term, nine different curves (as a function of VPD) for the sign term, and 81 different curves for the ET response to VPD ( $\frac{\partial \;ET}{\partial \;VPD}$). To aid visualization, we can examine a subset of nine of these 81 curves, defined by the minimum, median, and maximum values for both the scaling and sign term (Figure 1). The range of ET responses to VPD varies from those where ET almost always decreases with increasing VPD (water conservative), to those where ET almost always increases with increasing VPD (water intensive). Additionally, for some parameters, whether ET will increase or decrease with increasing VPD depends on atmospheric demand (Figure 1).

**Figure 2:**
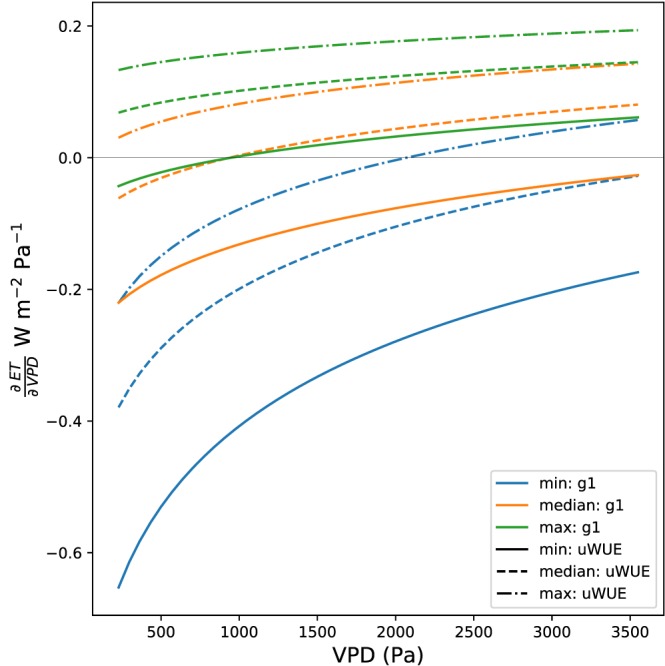
The functional form of $\frac{\partial \;ET}{\partial \;VPD}$ evaluated at the median value of the scaling term, for varying values of *g*_1_ and *u* *W* *U* *E* as given in Table 2. ET = evapotranspiration; uWUE = underlying Water Use Efficiency; VPD = vapor pressure deficit.

Examining the sign term independent of the scaling term illuminates the role of the two plant parameters, *g*_1_ and *uWUE*, in determining the degree to which the response is water conservative ( $\frac{\partial \;ET}{\partial \;VPD}<0$) or water intensive ( $\frac{\partial \;ET}{\partial \;VPD}>0$; Figure 2). Higher *g*_1_ and *uWUE* shift the curve toward increasing ET responses with VPD (water intensive), and smaller *g*_1_ values lead to a larger VPD dependence of the response (i.e., ET response is a stronger function of VPD). However, care should be exercised when interpreting the range of ET responses, because *g*_1_ and *uWUE* should generally be anticorrelated due to their dependencies on *λ*. As *λ* increases, *g*_1_ should increase, and *uWUE* should decrease. Because high and low values of *uWUE* and *g*_1_ were determined independently from each other in previous literature, plant types with anomalously high or low values for *both* *g*_1_ and *uWUE* should be relatively rare. However, some plant types do exhibit cooccurring high values for *g*_1_ and *uWUE*. For example, C3 crops have both the highest *g*_1_ value in Franks et al. (2017) and the highest *uWUE* value in Zhou et al. (2015).

**Figure 3:**
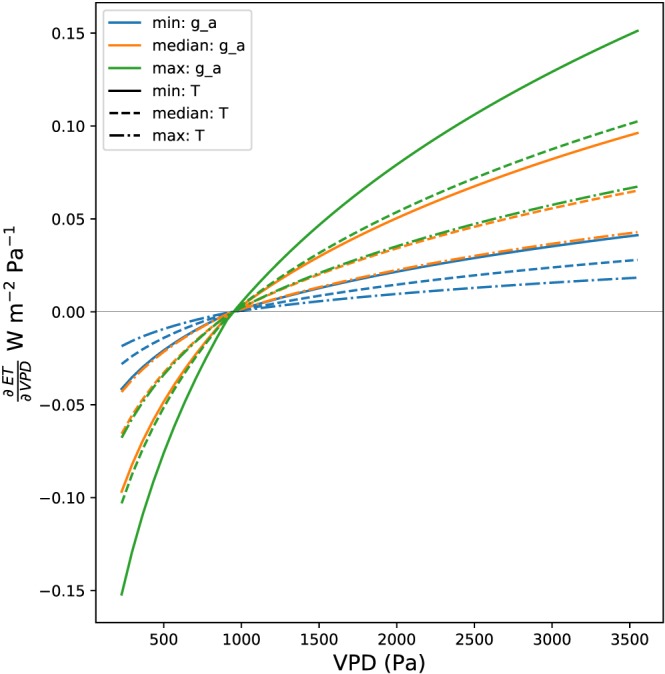
The functional form of $\frac{\partial \;ET}{\partial \;VPD}$ evaluated at the median value of the sign term, for varying values of *g*_a_ and *T* as given in Table 2. ET = evapotranspiration; VPD = vapor pressure deficit.

Examining the scaling term independent of the sign term shows how aerodynamic conductance (communication between the atmosphere and the surface) and temperature (controls the efficiency of energy conversion to latent heat through Δ) amplify or suppress the plant response represented in the sign term (Figure 3). Both lower temperatures and higher aerodynamic conductance lead to amplified ET response to VPD, with the variability of aerodynamic conductance resulting in a slightly higher variability of ET response relative to temperature.

## 4 Discussion

Interpreting both the derivation and results of this manuscript hinges on an understanding and appreciation for the usefulness of partial derivatives for understanding the behavior of complex systems in a simplified framework. Our results are a conceptual tool for answering the question, “What is the ET response to VPD with all other quantities held fixed?” This is useful, and we would argue that this is critical, for understanding complex responses when many quantities are varying simultaneously (Zhou et al., 2019). If we cannot understand the contribution of one term to observed variability, independent of other confounding factors, there is little hope of disentangling and understanding the relative role of many covarying quantities in determining observed variability. Equation (7) explicitly provides an estimate of the ET response to VPD, given assumptions about ecosystem plant characteristics through the parameters *g*_1_ and *uWUE*. Equation (7) is generic to external environmental factors and the time scale over which a VPD response acts and has a straightforward conceptual interpretation. It provides the ET response, given other quantities and parameters held fixed, subject to the assumptions outlined in section 2. So while the plant parameters *g*_1_ and *uWUE* may vary with plant species and environmental conditions like soil moisture, we can still assess the ET response to VPD for a given ecosystem state or for a given soil moisture condition.

However, given that *g*_1_ and *uWUE* can vary and they both have a large impact on the ET response (Figure 1 and 2), it is useful to examine how these quantities vary in previous literature, framed by our results on $\frac{\partial \;ET}{\partial \;VPD}$. In Zhou et al. (2015), most plant types' *uWUE* is similar to our median value, under which the sign of the ET response depends on VPD. However, crops, shrubs, and grass are exceptions; they exhibit an *uWUE* different than the median value in Table 2. Crops, which we expect to prioritize carbon uptake over water conservation, have a higher *uWUE* value closer to our maximum value. This higher *uWUE* results in a higher likelihood for an increasing ET response to VPD, which matches intuition given that we expect crops to keep stomata open for access to carbon, at the cost of increased water loss during high VPD. Shrubs, and to a lesser extent grass, have an *uWUE* closer to the minimum *uWUE*. For these plant types, we would then expect a decrease of ET in response to VPD. It is important to note that, while Zhou et al. (2015) did not examine the role of soil moisture for within‐plant‐type variability of *uWUE*, we might expect some variation, especially in extreme cases when soil water becomes a limiting factor (see supporting information).

The ecosystem‐scale results of Medlyn et al. (2017) did not display the robust relationship between plant type and plant parameter (*g*_1_) that was demonstrated by Zhou et al. (2015) for *uWUE*. Given that there is a robust relationship between *g*_1_ and plant type in the leaf‐scale results of Lin et al. (2015) and the ecosystem‐scale results of Zhou et al. (2015) rely on the same underlying theory as in Medlyn et al. (2017), the ambiguous results in Medlyn et al. (2017) could be due to noise in ecosystem‐scale observations, the consequences of imposing a model structure in some of their *g*_1_ estimations, or real ecosystem‐scale within‐plant‐type variability (e.g., multiple species impacting the relationship within the eddy covariance footprint). These differences between leaf‐scale behavior and ecosystem‐scale behavior highlight the importance of understanding how and why slope parameters show increased variability at the ecosystem scale, as argued by Medlyn et al. (2017). However, if we assume that there is some real analogy between leaf‐scale results in Lin et al. (2015) and ecosystem‐scale behavior and that some of the ambiguity in Medlyn et al. (2017) is due to model and/or observational error, then we can use the relationships between *g*_1_ and plant characteristics defined by Lin et al. (2015) to frame the ET response to VPD. This is subject to the strong caveat that we still do not fully understand *g*_1_ behavior at the ecosystem scale. Extrapolating the results of Lin et al. (2015; e.g., Figure 2) to the ecosystem scale would reveal the following relationships between *g*_1_ and plant types:

- C3 plants would have a generally higher *g*_1_ than C4 plants.
- Crops would have a larger *g*_1_ than shrubs, grass, and angiosperm trees, which have a higher *g*_1_ than gymnosperm trees.
- Tropical and temperate climates would generally be characterized by plants with a higher *g*_1_ than arctic and boreal climates.

For all of the above relationships, a higher *g*_1_ means a higher likelihood of a positive ET response to increasing VPD (water intensive) and a smaller VPD dependence of the ET response.

Our theoretical results highlight the variability of ET response to VPD as a function of both climate and plant characteristics, especially water usage strategy. Whether an ecosystem increases or decreases ET in response to VPD will vary depending on vegetation and climate. Generalizations about ET response to VPD therefore require thoughtful consideration of ecosystem characteristics; statements such as “ET increases with warming due to increases in VPD” may be false depending on the water conservation strategy of a given ecosystem (Lemordant et al., 2018).

Additionally, the sensitivity of ET response to plant parameters highlights the importance of understanding and developing stomatal conductance models and parameters for the ecosystem scale. In particular, why and how *g*_1_ behavior at the leaf scale is not analogous to the ecosystem scale must be understood. While the derivation here was explicit about the assumption of a constant *g*_1_ term with respect to the VPD perturbation, many sophisticated land surface models and Earth system models employing stomatal conductance models make similar assumptions about the stationarity of VPD slope terms (Franks et al., 2017; Lawrence et al., 2019; Niu et al., 2011; Rogers et al., 2017). Many models will assume a constant VPD slope term (e.g., *g*_1_) within a given plant functional type, and research derived from these models often does not acknowledge limitations of this assumption or the difficulty in the literature with quantifying an ecosystem‐scale slope parameter (Medlyn et al., 2017). Given that something so fundamental as the ET response to VPD varies strongly with *g*_1_, we must invest more in understanding *g*_1_'s behavior at the ecosystem scale and the efficacy of models using constant slope terms for ecosystem‐scale fluxes. Fortunately, our framework, and the new approach of using robust ecosystem‐scale ratios (*uWUE*) to remove the implicit dependence of stomatal conductance on ET, is flexible enough to be applied to any stomatal conductance model that contains a dependence on GPP.

So far, we have discussed the role of different parameters in modulating the ET response. However, the form of the ET response is imposed by our choice of stomatal conductance model. We now examine how stomatal model choice, and specifically the exponent of the VPD dependence in the stomatal conductance model, can impact the general form of the ET‐VPD relationship. There is a theoretical basis for the square root VPD dependence in both the stomatal conductance model and *uWUE* based on the assumption that stomata behave to maximize carbon gain while minimizing water loss, which observations also generally support (Lin et al., 2015; Lloyd, 1991; Medlyn et al., 2011, 2017; Zhou et al., 2014, 2015). However, some purely empirical results that fit the exponent of the VPD dependence to data have shown that it may vary slightly from 1/2, suggesting that stomata, as well as ecosystem‐scale quantities based on stomata theory, may not always function optimally (Lin et al., 2018; Zhou et al., 2015). Specifically with regard to *uWUE*, one would not expect that this ecosystem‐scale WUE quantity will respond to VPD exactly analogously to stomata. Direct soil evaporation's contributions to ET should shift the exponent of the VPD dependence. The results from Zhou et al. (2015) corroborate this: They found a mean empirically fit exponential VPD dependence of 0.55, varying slightly from the theoretically optimal value of 0.5 for AmeriFlux sites. Results in Lin et al. (2018) also show variance in the empirical exponent of the VPD dependence in the stomatal conductance model, but interpretation of this variance is more difficult as Lin et al. (2018) do not handle the GPP dependence of stomatal conductance in a directly analogous manner to the optimal theory in Medlyn et al. (2011) and Medlyn et al. (2017). Regardless, given that these recent results on the relationship between VPD, GPP, and ET (Medlyn et al., 2011, 2017; Zhou et al., 2014, 2015) form the backbone of our analysis and are what allowed us to derive an explicit ET expression for the first time (equation (6)), we will analyze if and how assumptions about the exponent of the VPD dependence impact the shape of the ET‐VPD dependence. This analysis is also important to understand whether the choice of stomatal conductance model alters the fundamental behavior of the ET‐VPD relationships, as many commonly used models utilize a VPD exponent other than the 1/2 suggested by optimal theory (e.g., Leuning, 1990 which uses an exponent of 1). It is useful to understand how the choice of model imposes the functional form of the ET‐VPD relationship, given its fundamental importance for land‐atmosphere interactions.

### 4.1 Functional Form of ET Dependence on VPD and Its Relation to the VPD Exponent

The results presented in section 3 indicates that for a given *uWUE* and *g*_1_, the ET dependence on VPD should be concave upward. In other words, there should be some local minimum in ET at a critical VPD_crit_, assuming the scaling and plant terms (e.g., aerodynamic conductance, Δ, *g*_1_, and *uWUE*) are held fixed. This result warrants further investigation, because to our knowledge, no earlier work has derived the theoretical ecosystem‐scale relationship between ET and VPD in an energy balance framework while controlling for other environmental conditions. In particular, there is an apparent lack of consensus over whether the shape of the ET‐VPD curve should be concave upward (our result) or concave downward in the absence of dramatic water stress. Given that understanding the ET‐VPD relationship of the one‐way plant response is fundamental to hypothesizing about any feedbacks between the land surface and the atmosphere, we analyze why our derived ET‐VPD relationship is concave upward, particularly with respect to the exponent of VPD dependence in *uWUE* and the Medlyn unified stomatal conductance model, as other models and empirical results have suggested exponents varying from 1/2 (Leuning, 1990; Zhou et al., 2015; Lin et al., 2018).

By introducing *n*, the exponent of VPD in *uWUE*, and *m*, the exponent of VPD in the stomatal conductance model, we can free our theory from assumptions about VPD dependence:

(10) $$g_{s}=\frac{R\;T}{P}1.6\left(1+\frac{g*}{VPD^{m}}\right)\frac{*WUE\;ET}{c_{a}\;VPD^{n}},$$

where

$$*WUE=\frac{GPP}{ET}VPD^{n},$$

and g* is a generic slope parameter of units *VPD* ^*m*^. To determine how the exponent *n* and *m* alter the shape of the ET‐VPD dependence, we find the roots of the second derivative of ET, using equation (10) for stomatal conductance (*g*_s_), with respect to VPD:

(11) $$\frac{\partial^{2}\;ET}{\partial \;VPD^{2}}=0\;\forall \;\frac{VPD^{m}}{g*}=\frac{m\left(m-2n-\sqrt{m^{2}-4mn+2m-4n^{2}+4n+1}+1\right)}{2n\left(n-1\right)}-1.$$

**Figure 4:**
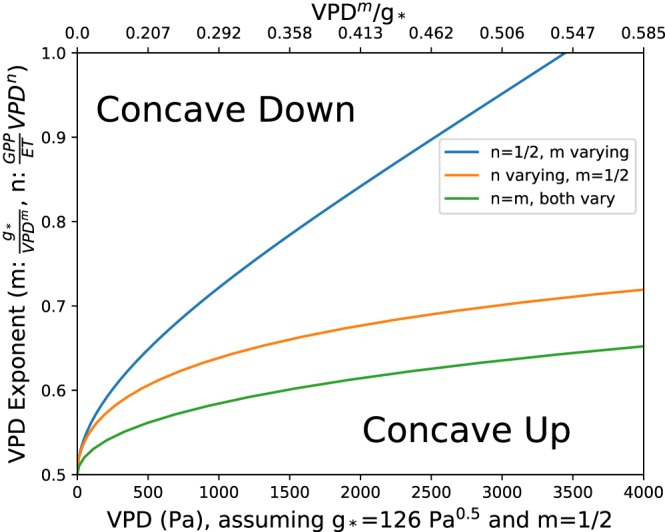
Solutions corresponding to inflection points between concave up and concave down ET‐VPD curves (equation (11)) for three specific scenarios. Solutions are defined in terms of a nondimensional VPD (*V* *P* *D* ^*m*^/g_*_), but to aid physical interpretation, the horizontal axis is additionally provided in terms of dimensionalized VPD assuming *m*=1/2 and g_*_=110Pa^1/2^ (average of all plant functional type *g*_1_). The vertical axis has a different interpretation depending on the solution curve. For the blue line (*m* varying), it corresponds to *m*; for the orange line (*n* varying), it corresponds to *n*; and for the green line, it corresponds to the value of both *n* and *m* (*n*=*m*). Regions of the parameter space that correspond to concave up and concave down results are labeled: For each curve, the parameter space “below” the curve corresponds to a concave up relationship, while the parameter space “above” the curve corresponds to a concave down relationship. ET = evapotranspiration; GPP = gross primary production; VPD = vapor pressure deficit.

With this result, we have defined the family of curves separating concave up from concave down ET solutions (Figure 4). These curves are only functions of the exponent of the VPD dependence and a quantity we call nondimensional VPD (*VPD* ^*m*^/g*). Several important relations reveal themselves from equation (11):

- For optimal behavior (*n*, *m* = 1/2), the ET‐VPD curve will be concave up regardless of the magnitude of the plant constants *g*_1_ and *uWUE*. Therefore, the general concave up nature of our results, given an assumption of optimal behavior, is insensitive to plant type.
- For all physically possible exponents of VPD dependence (*n*,*m*), whether the solution is concave up or concave down does not depend on *WUE.
- In general, increasing the exponent of VPD dependence for either *WUE or g_*_ increases the likelihood of a concave down result. Additionally, as the exponent of VPD dependence increases from the optimum value of 1/2, whether the curve is concave upward or concave downward becomes a function of the plant‐specific slope parameter g_*_, through nondimensional VPD (*VPD* ^*m*^/g_*_). Because the exponent of the VPD dependencies is capable of altering the fundamental shape of ET‐VPD dependence, future research investment in understanding the exact VPD dependence of stomatal conductance and further reconciliation of empirical and theoretical stomatal and ecosystem behavior should be prioritized.

While it is possible (and even likely) that in the future, some other form of VPD dependence is derived, at present (Medlyn et al., 2011) and (Zhou et al., 2014) established *n*=*m*=1/2 as the most likely candidate given current theory and empirical data. Additionally, we argue that a concave up result matches physical intuition more than a concave down result. Plants must maintain nutrient and sugar transport through the phloem and xylem. To accomplish this, stomata must remain slightly open (De Schepper et al., 2013; Nikinmaa et al., 2013; Ryan & Asao, 2014). Furthermore, even if complete stomatal closure were possible, cuticular water loss and (at the ecosystem‐scale) direct soil evaporation are still sources of ET which increase with VPD, independent of stomatal closure. Therefore, in the limit as VPD becomes large and we assume plants are exercising all strategies to reduce ET, any further increase in VPD should result in an increase in ET through cuticular water loss and/or direct soil evaporation. This inevitable transition from conditions when stomata respond strongly to VPD to conditions when stomata response is asymptoting toward full closure would cause a concave up ET‐VPD curve, which is matched by our theory. In short, plant response becomes more limited as VPD increases, while atmospheric demand monotonically increases with VPD, leading to the result that atmospheric demand dominates plant response when atmospheric demand is high.

This analysis allows us to understand the theoretical shape of the ET response to VPD with environmental conditions held fixed. Accomplishing this with purely statistical methods applied to flux observations would be very difficult, given the relatively fast time scale of plant response and the nonstationarity of (solar forced) environmental conditions over the relatively coarse (half hourly) flux estimates (which is required to obtain robust eddy covariance statistics). Our results on the shape of the ET‐VPD curve with environmental conditions held fixed can be built upon with future work examining how changes in VPD and environmental conditions (e.g., soil water storage) feedback upon one another. In the soil water storage example, over very long time scales, extremely high VPD perturbations coupled with no precipitation could result in decreases in soil water storage such that water becomes limiting. This could be represented by an extension of our framework in which *λ* is allowed to decrease with decreasing soil water, increasing *uWUE*, and decreasing *g*_1_. Here we focus our results by framing them with previous literature to build baseline intuition for ET‐VPD dependence.

## 5 Conclusions

We derived a new form of PM using the concept of semiempirical optimal stomatal regulation (Lin et al., 2015; Medlyn et al., 2011) and near‐constant *uWUE* (Zhou et al., 2015) to remove the implicit dependence of stomatal conductance on GPP and ET. With our new form of PM, we developed a theory for when an ecosystem will tend to reduce or increase ET with increasing VPD, which we framed using previous literature exploring the relationship between plant parameters, plant types, and climate. The goal was to understand the range of possible ET responses to VPD and develop some intuition for how the ET response may vary with plant types and climate. This intuition can be used to disentangle land‐atmosphere feedbacks in more complicated scenarios and will aid interpretation of observations and more sophisticated models.

ET response to VPD can vary from strongly water conservative (ET decreasing in response to increasing VPD) to strongly water intensive (ET increasing in response to VPD), which is indicative of the diversity of possible plant water conservation strategies. Higher *uWUE* and *g*_1_ values increase the likelihood of a positive ET response, while decreasing temperature and increasing g_a_ amplify the magnitude of the response. Previous literature (Lin et al., 2015; Zhou et al., 2015) suggests that crops, through association with higher *g*_1_ and *uWUE*, are more likely to exhibit a positive ET response to VPD. Shrubs (lower *uWUE*), C4 plants (lower *g*_1_), gymnosperm trees (lower *g*_1_), and plants in arctic and boreal climates (lower *g*_1_) are more likely to exhibit a negative ET response to VPD. However, interpretation of *g*_1_‐induced variability is partially muddied by ambiguity in applying leaf‐scale estimations of *g*_1_ to the ecosystem scale (Medlyn et al., 2017).

Our paper builds intuition for how plants respond to VPD perturbations. We show that given optimal stomatal function and fixed environmental conditions, the ET‐VPD dependence is theoretically concave upward, with ET increasing with increasing VPD as VPD increases past some critical value where $\frac{\partial \;ET}{\partial \;VPD}=0$. However, future research should focus on fully understanding the functional form of VPD dependence, as this concave up result is sensitive to the exponent of VPD dependence, which we currently believe is 1/2 for both uWUE and the stomatal conductance model (Medlyn et al., 2011; Zhou et al., 2014). Indeed, this sensitivity to the exponent of VPD dependence is an important result itself: Land surface models, including those used in Earth system models for climate forecasts, employ different assumptions about the exponent of VPD dependence in stomatal conductance (e.g., Ball et al., 1987; Leuning, 1990; Medlyn et al., 2011), and these assumptions can fundamentally change the relationship between ET and VPD from one that is concave upward (local minimum in ET) to one that is concave downward (local maximum in ET).

Our results are also applicable to understanding the impact of expected increases in VPD induced by global change. Plant physiological responses to direct CO_2_ effects (e.g., Lemordant et al., 2018; Swann et al., 2016) receive more attention than physiological response to indirect effects like increased VPD (Novick et al., 2016). Here we provide a framework for understanding ET response to VPD using a simplified model of two plant parameters. Feedbacks between the land and the atmosphere may alter the net response to a long time scale global VPD perturbation, but our focus on the one‐way plant response to a VPD perturbation in the atmospheric boundary layer is an important first step to disentangling such feedbacks, both in observations and model simulations of the present and future. By removing PM's dependence on implicit relationships between GPP, VPD, and ET, we allow for explicit future analysis of plant‐VPD feedbacks in the atmospheric boundary layer (equation (6)). Our approach can be extended to examine varying plant response to more nuanced consideration of plant type and climate. Any plant physiological heterogeneity or feedback that can be conceptualized with shifts in *g*_1_ (e.g., Lin et al., 2015; Medlyn et al., 2017) and/or *uWUE* (e.g., Zhou et al., 2014) is representable within our framework, which opens the door for a hierarchy of more sophisticated climate‐ and plant‐specific analyses of ET sensitivity to environmental variables (including VPD). We argue that such simplified conceptual frameworks are critical tools for disentangling land‐atmosphere feedbacks at various scales, from diurnal to seasonal and beyond, and to characterize ET response in a warmer, atmospherically drier, and enriched CO_2_ world.

## Supporting information



Supporting Information S1
