# Supplementary material for: When Does Vapor Pressure Deficit Drive or Reduce Evapotranspiration?
Source: J Adv Model Earth Syst. 2019 Oct 28;11(10):3305–20. doi: 10.1029/2019MS001790 (PMC6919419; doi:10.1029/2019MS001790)
Supplement: Supplementary file 1 — Supporting Information S1 [file JAME-11-3305-s001.pdf]

Supporting Information for "When does vapor pressure deficit drive or reduce evapotranspiration?"

A. Massmann[1], P. Gentine[1], C. Lin[1,2]

[1]Department of Earth and Environmental Engineering, Columbia University, New York, NY 10027

[2]State Key Laboratory of Hydroscience and Engineering, Department of Hydraulic Engineering, Tsinghua University, Beijing, CN 100084

Contents of this file

1. Figures S1 to S68
2. Table S1

Introduction

The manuscript analyzes the partial derivative of ET with respect to VPD. This assumes that all other quantities remain fixed, including the plant parameters g_1 and uWUE (and by extension, λ). In reality, these parameters may vary with environmental conditions, and specifically soil moisture. However because soil moisture *only* enters the partial derivative directly through these plant terms, if the plant parameters are weak functions of soil moisture then the theory can be directly applied to a broader range of conceptual VPD scenarios, including observed compound events between high VPD and low soil moisture Zhou et al. (2019). To help the reader assess the soil moisture dependence of uWUE (and partially by extension λ and g_1), we provide here figures showing the distribution

of uWUE with SWC for each of 66 FLUXNET sites from the FLUXNET-2015 database. The functional relationship between uWUE and SWC varies, with a mix of sites showing strong and weak SWC-dependence. For all sites the ratio of signal to noise is very low, an unfortunate consequence of taking a ratio of two highly uncertain eddy-covariance derived fluxes. In general we find this analysis inconclusive.

The paper does not rely on assumptions about uWUE's functional relationship with soil moisture so we do not include the figures in the body of the manuscript. But given that constant uWUE and g_1 assumptions can make our theory more useful to the reader we provide the figures for their interpretation, and motivation for future research.

Additionally, we include a figure showing the joint distribution between saturation vapor pressure and relative humidity calculated from the FLUXNET-2015 data. Relative humidity and saturation vapor pressure are much more independent than saturation vapor pressure and VPD, and we use an assumption that relative humidity and saturation vapor pressure can be approximated as independent in order to evaluate $\frac{\partial ET}{\partial VPD}$. Please note that at a given site, the relationship may be more or less independent depending on the hydroclimate.

1. Description of data

1.1. Data

We use both meteorological and eddy-covariance data from the FLUXNET2015 database (data available at `https://fluxnet.fluxdata.org/data/fluxnet2015-dataset/`), including all Tier 1 sites with at least four years of data. Sixty-six sites met these requirements, and were grouped into nine plant functional types (PFT) according to the International Geosphere-Biosphere Programme vegetation classification scheme

Loveland et al. (1999): cropland (CRO), grass (GRA), deciduous broadleaf forest (DBF), evergreen broadleaf forest (EBF), evergreen needleleaf forest (ENF), mixed forest (MF), closed shrub (CSH), savannah (SAV), and woody savannah (WSA).

We filter and quality control the FLUXNET-2015 data using a similar procedure as Zhou et al. (2015):

- Only measured or highest ("good") quality gapfilled data, according to quality control flags, are used.

- To isolate the growing season, we only use days in which the average Gross Primary Productivity (GPP) exceeds 10% of the observed 95th percentile of GPP for a given site. GPP is calculated using the nighttime respiration partitioning method.

- We remove days with rain and the day following to avoid issues with rain interception and sensor saturation at high relative humidity (Medlyn et al. (2017)).

- For SWC measurements, we use the shallowest observed layer available at each site.

Additionally, as in Lin et al. (2018), we restrict data to the daytime, which is identified when downwelling shortwave radiation is greater than 50 W m^{-2} and sensible heat flux is greater than 5 W m^{-2}. To reduce the chance of sensor saturation at high relative humidity, we remove all time steps for which VPD is less than .01 kPa, and to reduce errors at low windspeeds we remove all periods with wind magnitudes less than 0.5 m s^{-1}. Timesteps with negative observed GPP or ET are also removed, and we aggregate half hourly data to hourly averages to reduce noise Lin et al. (2018). After these quality control procedures, 400,983 upscaled hourly observations remain.

References

Anthoni, P. M., Knohl, A., Rebmann, C., Freibauer, A., Mund, M., Ziegler, W., ... Schulze, E.-D. (2004, dec). Forest and agricultural land-use-dependent CO2 exchange in Thuringia, Germany. *Global Change Biology*, *10*(12), 2005–2019. Retrieved from https://doi.org/10.1111%2Fj.1365-2486.2004.00863.x doi: 10.1111/j.1365-2486.2004.00863.x

Archibald, S. A., Kirton, A., van der Merwe, M. R., Scholes, R. J., Williams, C. A., & Hanan, N. (2009, feb). Drivers of inter-annual variability in net ecosystem exchange in a semi-arid savanna ecosystem, South Africa. *Biogeosciences*, *6*(2), 251–266. Retrieved from https://doi.org/10.5194%2Fbg-6-251-2009 doi: 10.5194/bg-6-251-2009

Aubinet, M., Chermanne, B., Vandenhaute, M., Longdoz, B., Yernaux, M., & Laitat, E. (2001, jul). Long term carbon dioxide exchange above a mixed forest in the Belgian Ardennes. *Agricultural and Forest Meteorology*, *108*(4), 293–315. Retrieved from https://doi.org/10.1016%2Fs0168-1923%2801%2900244-1 doi: 10.1016/s0168-1923(01)00244-1

Baldocchi, D., Chen, Q., Chen, X., Ma, S., Miller, G., Ryu, Y., ... Battles, J. (2010, nov). The dynamics of energy, water, and carbon fluxes in a blue oak (Quercus douglasii) savanna in California. In *Ecosystem function in Savannas* (pp. 135–151). CRC Press. Retrieved from https://doi.org/10.1201%2Fb10275-10 doi: 10.1201/b10275-10

Berbigier, P., Bonnefond, J.-M., & Mellmann, P. (2001, jun). CO2 and water vapour fluxes for 2 years above Euroflux forest site. *Agricultural and Forest Meteorology*, *108*(3), 183–197. Retrieved from https://doi.org/10.1016%2Fs0168-1923%2801%2900240-4 doi: 10.1016/s0168-1923(01)00240-4

Bergeron, O., Margolis, H. A., Black, T. A., Coursolle, C., Dunn, A. L., Barr, A. G.,

& Wofsy, S. C. (2007, jan). Comparison of carbon dioxide fluxes over three boreal black spruce forests in Canada. *Global Change Biology*, *13*(1), 89–107. Retrieved from https://doi.org/10.1111%2Fj.1365-2486.2006.01281.x doi: 10.1111/j.1365-2486.2006.01281.x

Beringer, J., Hutley, L. B., Hacker, J. M., Neininger, B., & U, K. T. P. (2011, nov). Patterns and processes of carbon, water and energy cycles across northern Australian landscapes: From point to region. *Agricultural and Forest Meteorology*, *151*(11), 1409–1416. Retrieved from https://doi.org/10.1016%2Fj.agrformet.2011.05.003 doi: 10.1016/j.agrformet.2011.05.003

Beringer, J., Hutley, L. B., McHugh, I., Arndt, S. K., Campbell, D., Cleugh, H. A., ... Wardlaw, T. (2016, oct). An introduction to the Australian and New Zealand flux tower network - OzFlux. *Biogeosciences*, *13*(21), 5895–5916. Retrieved from http://www.biogeosciences.net/13/5895/2016/https://doi.org/10.5194https://doi.org/10.5194{%}2Fbg-13-5895-2016 doi: 10.5194/bg-13-5895-2016

Beringer, J., Hutley, L. B., Tapper, N. J., & Cernusak, L. A. (2007, may). Savanna fires and their impact on net ecosystem productivity in North Australia. *Global Change Biology*, *13*(5), 990–1004. Retrieved from https://doi.org/10.1111%2Fj.1365-2486.2007.01334.x doi: 10.1111/j.1365-2486.2007.01334.x

Cernusak, L. A., Hutley, L. B., Beringer, J., Holtum, J. A., & Turner, B. L. (2011, nov). Photosynthetic physiology of eucalypts along a sub-continental rainfall gradient in northern Australia. *Agricultural and Forest Meteorology*, *151*(11), 1462–1470. Retrieved from https://doi.org/10.1016%2Fj.agrformet.2011.01.006 doi:

10.1016/j.agrformet.2011.01.006

Chiesi, M., Maselli, F., Bindi, M., Fibbi, L., Cherubini, P., Arlotta, E., ... Seufert, G. (2005, dec). Modelling carbon budget of Mediterranean forests using ground and remote sensing measurements. *Agricultural and Forest Meteorology*, *135*(1-4), 22–34. Retrieved from https://doi.org/10.1016%2Fj.agrformet.2005.09.011 doi: 10.1016/j.agrformet.2005.09.011

Cleverly, J., Boulain, N., Villalobos-Vega, R., Grant, N., Faux, R., Wood, C., ... Eamus, D. (2013, jul). Dynamics of component carbon fluxes in a semi-arid Acacia woodland, central Australia. *Journal of Geophysical Research: Biogeosciences*, *118*(3), 1168–1185. Retrieved from https://doi.org/10.1002%2Fjgrg.20101 doi: 10.1002/jgrg.20101

Cook, B. D., Davis, K. J., Wang, W., Desai, A., Berger, B. W., Teclaw, R. M., ... Heilman, W. (2004, nov). Carbon exchange and venting anomalies in an upland deciduous forest in northern Wisconsin, USA. *Agricultural and Forest Meteorology*, *126*(3-4), 271–295. Retrieved from https://doi.org/10.1016%2Fj.agrformet.2004.06.008 doi: 10.1016/j.agrformet.2004.06.008

Desai, A. R., Bolstad, P. V., Cook, B. D., Davis, K. J., & Carey, E. V. (2005, jan). Comparing net ecosystem exchange of carbon dioxide between an old-growth and mature forest in the upper Midwest, USA. *Agricultural and Forest Meteorology*, *128*(1-2), 33–55. Retrieved from https://doi.org/10.1016%2Fj.agrformet.2004.09.005 doi: 10.1016/j.agrformet.2004.09.005

Dragoni, D., Schmid, H. P., Wayson, C. A., Potter, H., Grimmond, C. S. B., & Randolph, J. C. (2011, jan). Evidence of increased net ecosystem productivity associated with a longer vegetated season in a deciduous forest in south-central Indiana, USA. *Global*

Change Biology, *17*(2), 886–897. Retrieved from https://doi.org/10.1111%2Fj.1365-2486.2010.02281.x doi: 10.1111/j.1365-2486.2010.02281.x

Fischer, M. L., Billesbach, D. P., Berry, J. A., Riley, W. J., & Torn, M. S. (2007, oct). Spatiotemporal variations in growing season exchanges of CO2, H2O, and sensible heat in agricultural fields of the southern Great Plains. *Earth Interactions*, *11*(17), 1–21. Retrieved from https://doi.org/10.1175%2Fei231.1 doi: 10.1175/ei231.1

Galvagno, M., Wohlfahrt, G., Cremonese, E., Rossini, M., Colombo, R., Filippa, G., ... Migliavacca, M. (2013, apr). Phenology and carbon dioxide source/sink strength of a subalpine grassland in response to an exceptionally short snow season. *Environmental Research Letters*, *8*(2), 025008. Retrieved from https://doi.org/10.1088%2F1748-9326%2F8%2F2%2F025008 doi: 10.1088/1748-9326/8/2/025008

Garbulsky, M. F., Peñuelas, J., Papale, D., & Filella, I. (2008, dec). Remote estimation of carbon dioxide uptake by a Mediterranean forest. *Global Change Biology*, *14*(12), 2860–2867. Retrieved from https://doi.org/10.1111%2Fj.1365-2486.2008.01684.x doi: 10.1111/j.1365-2486.2008.01684.x

Goldstein, A., Hultman, N., Fracheboud, J., Bauer, M., Panek, J., Xu, M., ... Baugh, W. (2000, mar). Effects of climate variability on the carbon dioxide, water, and sensible heat fluxes above a ponderosa pine plantation in the Sierra Nevada (CA). *Agricultural and Forest Meteorology*, *101*(2-3), 113–129. Retrieved from https://doi.org/10.1016%2Fs0168-1923%2899%2900168-9 doi: 10.1016/s0168-1923(99)00168-9

Grünwald, T., & Bernhofer, C. (2007, jul). A decade of carbon, water and energy flux measurements of an old spruce forest at the Anchor Station Tharandt. *Tellus B*, *59*(3). Retrieved from https://doi.org/10.3402%2Ftellusb.v59i3.17000 doi:

10.3402/tellusb.v59i3.17000

Hinko-Najera, N., Isaac, P., Beringer, J., van Gorsel, E., Ewenz, C., McHugh, I., ... Arndt, S. K. (2017). Net ecosystem carbon exchange of a dry temperate eucalypt forest. *Biogeosciences*, *14*(16), 3781–3800. Retrieved from https://www.biogeosciences.net/14/3781/2017/ doi: 10.5194/bg-14-3781-2017

Hutley, L. B., Beringer, J., Isaac, P. R., Hacker, J. M., & Cernusak, L. A. (2011, nov). A sub-continental scale living laboratory: Spatial patterns of savanna vegetation over a rainfall gradient in northern Australia. *Agricultural and Forest Meteorology*, *151*(11), 1417–1428. Retrieved from https://doi.org/10.1016%2Fj.agrformet.2011.03.002 doi: 10.1016/j.agrformet.2011.03.002

Imer, D., Merbold, L., Eugster, W., & Buchmann, N. (2013, sep). Temporal and spatial variations of soil CO_2, CH_4 and N_2O fluxes at three differently managed grasslands. *Biogeosciences*, *10*(9), 5931–5945. Retrieved from https://doi.org/10.5194%2Fbg-10-5931-2013 doi: 10.5194/bg-10-5931-2013

Irvine, J., Law, B. E., Martin, J. G., & Vickers, D. (2008, dec). Interannual variation in soil CO2 efflux and the response of root respiration to climate and canopy gas exchange in mature ponderosa pine. *Global Change Biology*, *14*(12), 2848–2859. Retrieved from https://doi.org/10.1111%2Fj.1365-2486.2008.01682.x doi: 10.1111/j.1365-2486.2008.01682.x

Knohl, A., Schulze, E.-D., Kolle, O., & Buchmann, N. (2003, sep). Large carbon uptake by an unmanaged 250-year-old deciduous forest in central Germany. *Agricultural and Forest Meteorology*, *118*(3-4), 151–167. Retrieved from https://doi.org/10.1016%2Fs0168-1923%2803%2900115-1 doi: 10.1016/s0168-1923(03)00115-1

Kurbatova, J., Li, C., Varlagin, A., Xiao, X., & Vygodskaya, N. (2008, jul). Modeling carbon dynamics in two adjacent spruce forests with different soil conditions in Russia. *Biogeosciences*, *5*(4), 969–980. Retrieved from https://doi.org/10.5194%2Fbg-5-969-2008 doi: 10.5194/bg-5-969-2008

Leuning, R., Cleugh, H. A., Zegelin, S. J., & Hughes, D. (2005, apr). Carbon and water fluxes over a temperate eucalyptus forest and a tropical wet/dry savanna in Australia: measurements and comparison with MODIS remote sensing estimates. *Agricultural and Forest Meteorology*, *129*(3-4), 151–173. Retrieved from https://doi.org/10.1016%2Fj.agrformet.2004.12.004 doi: 10.1016/j.agrformet.2004.12.004

Lin, C., Gentine, P., Huang, Y., Guan, K., Kimm, H., & Zhou, S. (2018). Diel ecosystem conductance response to vapor pressure deficit is suboptimal and independent of soil moisture. *Agricultural and Forest Meteorology*, *250*, 24–34.

Lindauer, M., Schmid, H., Grote, R., Mauder, M., Steinbrecher, R., & Wolpert, B. (2014, oct). Net ecosystem exchange over a non-cleared wind-throw-disturbed upland spruce forest—measurements and simulations. *Agricultural and Forest Meteorology*, *197*, 219–234. Retrieved from https://doi.org/10.1016%2Fj.agrformet.2014.07.005 doi: 10.1016/j.agrformet.2014.07.005

Loubet, B., Laville, P., Lehuger, S., Larmanou, E., Fléchard, C., Mascher, N., … Cellier, P. (2011, mar). Carbon, nitrogen and greenhouse gases budgets over a four years crop rotation in northern France. *Plant and Soil*, *343*(1-2), 109–137. Retrieved from https://doi.org/10.1007%2Fs11104-011-0751-9 doi: 10.1007/s11104-011-0751-9

Loveland, T. R., Zhu, Z., Ohlen, D. O., Brown, J. F., Reed, B. C., & Yang, L. (1999). An analysis of the IGBP global land-cover characterization process. *Photogrammetric*

Engineering and Remote Sensing, *65*, 1021–1032.

Ma, S., Baldocchi, D. D., Xu, L., & Hehn, T. (2007, dec). Inter-annual variability in carbon dioxide exchange of an oak/grass savanna and open grassland in California. *Agricultural and Forest Meteorology*, *147*(3-4), 157–171. Retrieved from https://doi.org/10.1016%2Fj.agrformet.2007.07.008 doi: 10.1016/j.agrformet.2007.07.008

Marcolla, B., Cescatti, A., Manca, G., Zorer, R., Cavagna, M., Fiora, A., ... Zampedri, R. (2011, sep). Climatic controls and ecosystem responses drive the inter-annual variability of the net ecosystem exchange of an alpine meadow. *Agricultural and Forest Meteorology*, *151*(9), 1233–1243. Retrieved from https://doi.org/10.1016%2Fj.agrformet.2011.04.015 doi: 10.1016/j.agrformet.2011.04.015

Marcolla, B., Pitacco, A., & Cescatti, A. (2003, jul). Canopy architecture and turbulence structure in a coniferous forest. *Boundary-Layer Meteorology*, *108*(1), 39–59. Retrieved from https://doi.org/10.1023%2Fa%3A1023027709805 doi: 10.1023/a:1023027709805

McHugh, I. D., Beringer, J., Cunningham, S. C., Baker, P. J., Cavagnaro, T. R., Nally, R. M., & Thompson, R. M. (2017, jun). Interactions between nocturnal turbulent flux, storage and advection at an "ideal" eucalypt woodland site. *Biogeosciences*, *14*(12), 3027–3050. Retrieved from https://doi.org/10.5194%2Fbg-14-3027-2017 doi: 10.5194/bg-14-3027-2017

Medlyn, B. E., De Kauwe, M. G., Lin, Y.-S., Knauer, J., Duursma, R. A., Williams, C. A., ... others (2017). How do leaf and ecosystem measures of water-use efficiency compare? *New Phytologist*, *216*(3), 758–770.

Merbold, L., Ardö, J., Arneth, A., Scholes, R. J., Nouvellon, Y., de Grandcourt, A.,

... Kutsch, W. L. (2009, jun). Precipitation as driver of carbon fluxes in 11 African ecosystems. *Biogeosciences*, *6*(6), 1027–1041. Retrieved from https://doi.org/10.5194%2Fbg-6-1027-2009 doi: 10.5194/bg-6-1027-2009

Merbold, L., Eugster, W., Stieger, J., Zahniser, M., Nelson, D., & Buchmann, N. (2014, feb). Greenhouse gas budget (CO2, CH4and N2O) of intensively managed grassland following restoration. *Global Change Biology*, *20*(6), 1913–1928. Retrieved from https://doi.org/10.1111%2Fgcb.12518 doi: 10.1111/gcb.12518

Meyer, W. S., Kondrlovà, E., & Koerber, G. R. (2015, mar). Evaporation of perennial semi-arid woodland in southeastern Australia is adapted for irregular but common dry periods. *Hydrological Processes*, *29*(17), 3714–3726. Retrieved from https://doi.org/10.1002%2Fhyp.10467 doi: 10.1002/hyp.10467

Mkhabela, M., Amiro, B., Barr, A., Black, T., Hawthorne, I., Kidston, J., ... Zha, T. (2009, may). Comparison of carbon dynamics and water use efficiency following fire and harvesting in Canadian boreal forests. *Agricultural and Forest Meteorology*, *149*(5), 783–794. Retrieved from https://doi.org/10.1016%2Fj.agrformet.2008.10.025 doi: 10.1016/j.agrformet.2008.10.025

Monson, R. K., Turnipseed, A. A., Sparks, J. P., Harley, P. C., Scott-Denton, L. E., Sparks, K., & Huxman, T. E. (2002, may). Carbon sequestration in a high-elevation, subalpine forest. *Global Change Biology*, *8*(5), 459–478. Retrieved from https://doi.org/10.1046%2Fj.1365-2486.2002.00480.x doi: 10.1046/j.1365-2486.2002.00480.x

Montagnani, L., Manca, G., Canepa, E., Georgieva, E., Acosta, M., Feigenwinter, C., ... Ziegler, W. (2009, apr). A new mass conservation approach to the study of CO2

advection in an alpine forest. *Journal of Geophysical Research*, *114*(D7). Retrieved from https://doi.org/10.1029%2F2008jd010650 doi: 10.1029/2008jd010650

Moors, E. (2012). *Water Use of Forests in The Netherlands* (Unpublished doctoral dissertation). Vrije Universiteit Amsterdam.

Moureaux, C., Debacq, A., Bodson, B., Heinesch, B., & Aubinet, M. (2006, sep). Annual net ecosystem carbon exchange by a sugar beet crop. *Agricultural and Forest Meteorology*, *139*(1-2), 25–39. Retrieved from https://doi.org/10.1016%2Fj.agrformet.2006.05.009 doi: 10.1016/j.agrformet.2006.05.009

Papale, D., Migliavacca, M., Cremonese, E., Cescatti, A., Alberti, G., Balzarolo, M., ... Valentini, R. (2014, dec). Carbon, water and energy fluxes of terrestrial ecosystems in Italy. In *The greenhouse gas balance of italy* (pp. 11–45). Springer Berlin Heidelberg. Retrieved from https://doi.org/10.1007%2F978-3-642-32424-6_2 doi: 10.1007/978-3-642-32424-6_2

Pilegaard, K., Ibrom, A., Courtney, M. S., Hummelshøj, P., & Jensen, N. O. (2011, jul). Increasing net CO2 uptake by a Danish beech forest during the period from 1996 to 2009. *Agricultural and Forest Meteorology*, *151*(7), 934–946. Retrieved from https://doi.org/10.1016%2Fj.agrformet.2011.02.013 doi: 10.1016/j.agrformet.2011.02.013

Powell, T. L., Bracho, R., Li, J., Dore, S., Hinkle, C. R., & Drake, B. G. (2006, dec). Environmental controls over net ecosystem carbon exchange of scrub oak in central Florida. *Agricultural and Forest Meteorology*, *141*(1), 19–34. Retrieved from https://doi.org/10.1016%2Fj.agrformet.2006.09.002 doi: 10.1016/j.agrformet.2006.09.002

Prescher, A.-K., Grünwald, T., & Bernhofer, C. (2010, jul). Land use regulates carbon

budgets in eastern Germany: From NEE to NBP. *Agricultural and Forest Meteorology*, *150*(7-8), 1016–1025. Retrieved from https://doi.org/10.1016%2Fj.agrformet.2010.03.008 doi: 10.1016/j.agrformet.2010.03.008

Raz-Yaseef, N., Billesbach, D. P., Fischer, M. L., Biraud, S. C., Gunter, S. A., Bradford, J. A., & Torn, M. S. (2015, dec). Vulnerability of crops and native grasses to summer drying in the U.S. southern Great Plains. *Agriculture, Ecosystems & Environment*, *213*, 209–218. Retrieved from https://doi.org/10.1016%2Fj.agee.2015.07.021 doi: 10.1016/j.agee.2015.07.021

Schmidt, M., Reichenau, T., Fiener, P., & Schneider, K. (2012, nov). The carbon budget of a winter wheat field: An eddy covariance analysis of seasonal and inter-annual variability. *Agricultural and Forest Meteorology*, *165*, 114–126. Retrieved from https://doi.org/10.1016%2Fj.agrformet.2012.05.012 doi: 10.1016/j.agrformet.2012.05.012

Scott, R. L., Biederman, J. A., Hamerlynck, E. P., & Barron-Gafford, G. A. (2015, dec). The carbon balance pivot point of southwestern U.S. semiarid ecosystems: Insights from the 21st century drought. *Journal of Geophysical Research: Biogeosciences*, *120*(12), 2612–2624. Retrieved from https://doi.org/10.1002%2F2015jg003181 doi: 10.1002/2015jg003181

Scott, R. L., Hamerlynck, E. P., Jenerette, G. D., Moran, M. S., & Barron-Gafford, G. A. (2010, sep). Carbon dioxide exchange in a semidesert grassland through drought-induced vegetation change. *Journal of Geophysical Research*, *115*(G3). Retrieved from https://doi.org/10.1029%2F2010jg001348 doi: 10.1029/2010jg001348

Scott, R. L., Jenerette, G. D., Potts, D. L., & Huxman, T. E. (2009, nov). Effects of

seasonal drought on net carbon dioxide exchange from a woody-plant-encroached semi-arid grassland. *Journal of Geophysical Research*, *114* (G4). Retrieved from https://doi.org/10.1029%2F2008jg000900 doi: 10.1029/2008jg000900

Suni, T., Rinne, J., Reissel, A., Altimir, N., Keronen, P., Rannik, Ü., ... Vesala, T. (2003). Long-term measurements of surface fluxes above a Scots pine forest in Hyytiälä, southern Finland. *Boreal Environ. Res.*, *4*, 287–301.

Tedeschi, V., Rey, A., Manca, G., Valentini, R., Jarvis, P. G., & Borghetti, M. (2006, jan). Soil respiration in a Mediterranean oak forest at different developmental stages after coppicing. *Global Change Biology*, *12* (1), 110–121. Retrieved from https://doi.org/10.1111%2Fj.1365-2486.2005.01081.x doi: 10.1111/j.1365-2486.2005.01081.x

Thum, T., Aalto, T., Laurila, T., Aurela, M., Kolari, P., & Hari, P. (2007, nov). Parametrization of two photosynthesis models at the canopy scale in a northern boreal Scots pine forest. *Tellus B*, *59* (5). Retrieved from https://doi.org/10.3402%2Ftellusb.v59i5.17066 doi: 10.3402/tellusb.v59i5.17066

Valentini, R., Angelis, P., Matteucci, G., Monaco, R., Dore, S., & Mucnozza, G. E. S. (1996, jun). Seasonal net carbon dioxide exchange of a beech forest with the atmosphere. *Global Change Biology*, *2* (3), 199–207. Retrieved from https://doi.org/10.1111%2Fj.1365-2486.1996.tb00072.x doi: 10.1111/j.1365-2486.1996.tb00072.x

Verma, S. B., Dobermann, A., Cassman, K. G., Walters, D. T., Knops, J. M., Arkebauer, T. J., ... Walter-Shea, E. A. (2005, jul). Annual carbon dioxide exchange in irrigated and rainfed maize-based agroecosystems. *Agricultural and Forest Meteorology*, *131* (1-2), 77–96. Retrieved from https://doi.org/10.1016%2Fj.agrformet.2005.05.003 doi: 10.1016/j.agrformet.2005.05.003

Wick, B., Veldkamp, E., de Mello, W. Z., Keller, M., & Crill, P. (2005, aug). Nitrous oxide fluxes and nitrogen cycling along a pasture chronosequence in central Amazonia, Brazil. *Biogeosciences*, *2*(2), 175–187. Retrieved from https://doi.org/10.5194%2Fbg-2-175-2005 doi: 10.5194/bg-2-175-2005

Wohlfahrt, G., Hammerle, A., Haslwanter, A., Bahn, M., Tappeiner, U., & Cernusca, A. (2008, apr). Seasonal and inter-annual variability of the net ecosystem CO2 exchange of a temperate mountain grassland: Effects of weather and management. *Journal of Geophysical Research*, *113*(D8). Retrieved from https://doi.org/10.1029%2F2007jd009286 doi: 10.1029/2007jd009286

Zhou, S., Yu, B., Huang, Y., & Wang, G. (2015, may). Daily underlying water use efficiency for AmeriFlux sites. *Journal of Geophysical Research: Biogeosciences*, *120*(5), 887–902. doi: 10.1002/2015jg002947

Zhou, S., Zhang, Y., Park Williams, A., & Gentine, P. (2019). Projected increases in intensity, frequency, and terrestrial carbon costs of compound drought and aridity events. *Science Advances*, *5*(1). Retrieved from http://advances.sciencemag.org/content/5/1/eaau5740 doi: 10.1126/sciadv.aau5740

Zielis, S., Etzold, S., Zweifel, R., Eugster, W., Haeni, M., & Buchmann, N. (2014, mar). NEP of a Swiss subalpine forest is significantly driven not only by current but also by previous year's weather. *Biogeosciences*, *11*(6), 1627–1635. Retrieved from https://doi.org/10.5194%2Fbg-11-1627-2014 doi: 10.5194/bg-11-1627-2014

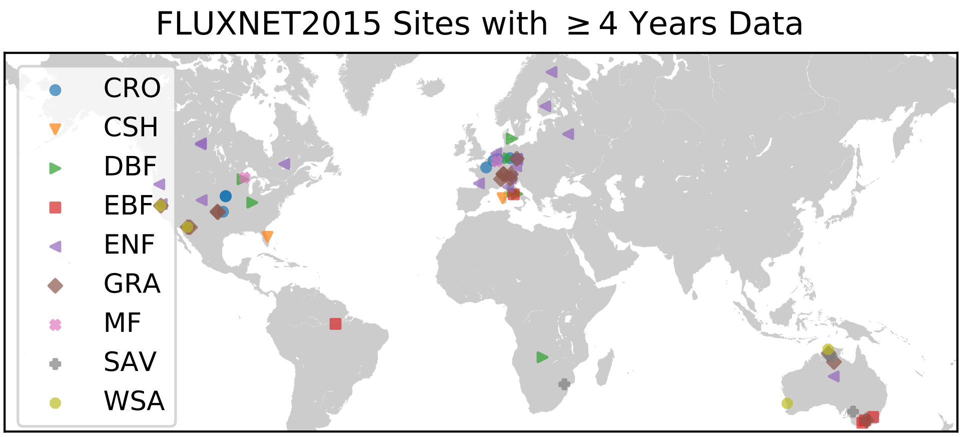

Figure S1. Plant functional type and location of FLUXNET2015 sites used in this analysis.

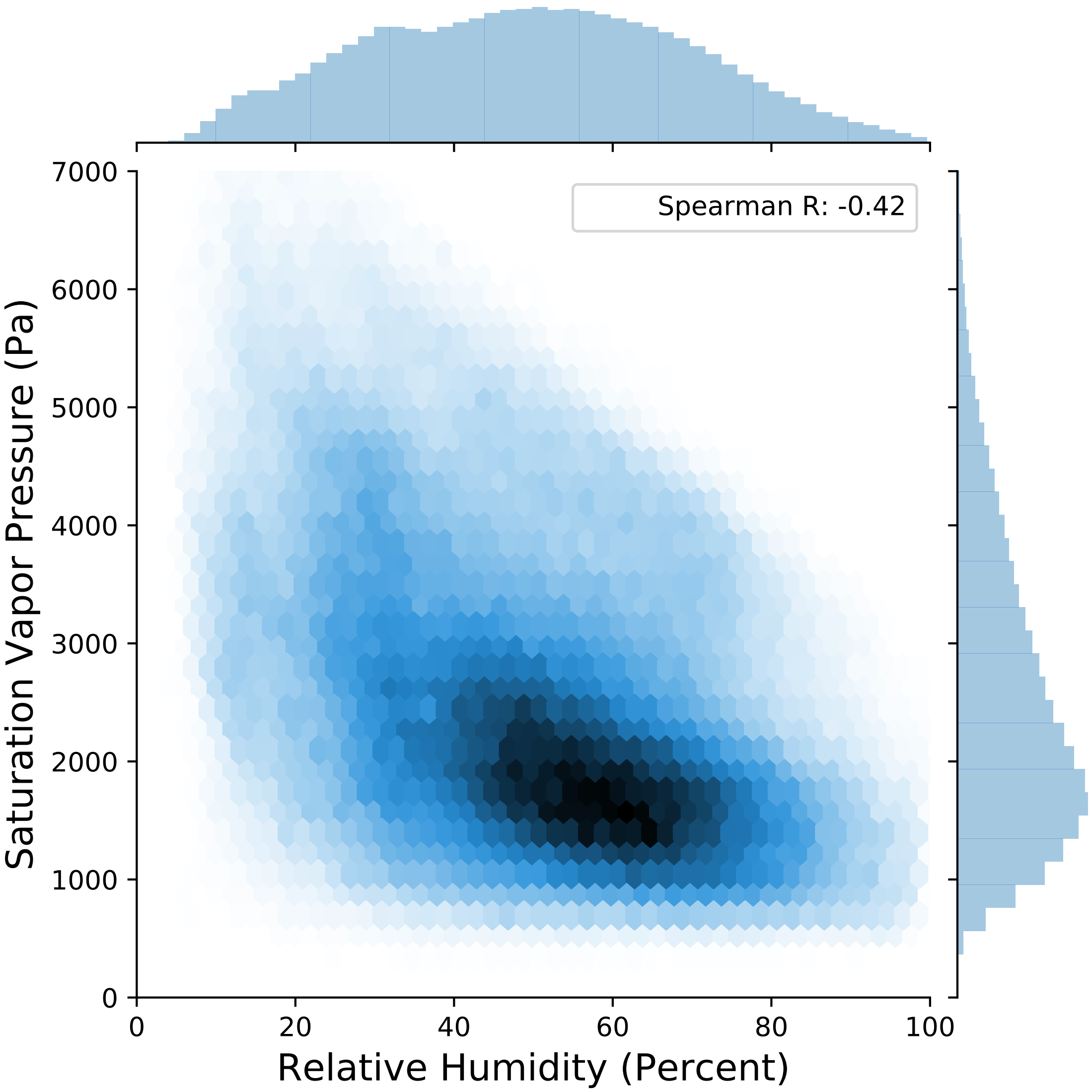

Figure S1. The joint distribution of relative humidity and saturation vapor pressure for the FLUXNET2015 dataset.

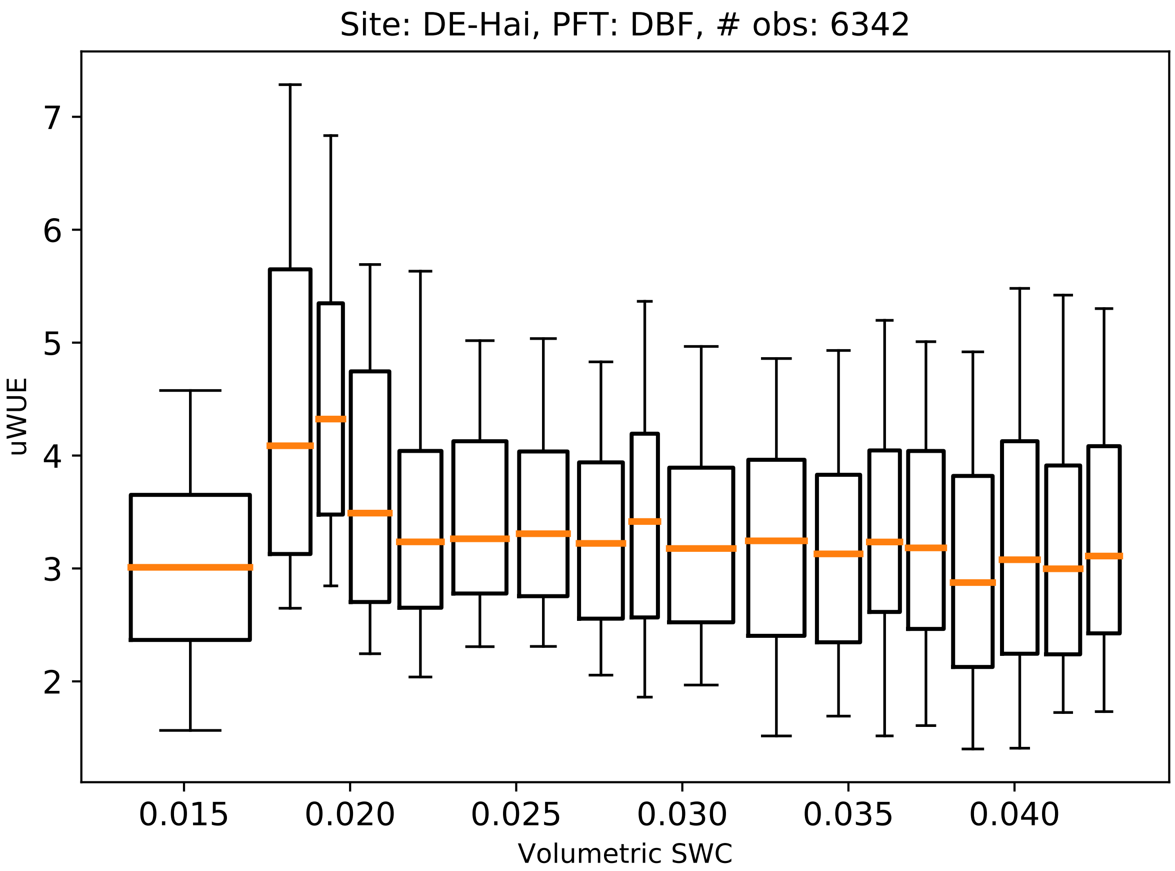

Figure S2. The relationship between uWUE and VPD at the FLUXNET site DE-Hai. Each box plot correspondes to 5% of the data. To aid visualization only the 0%-90% range of SWC bins are included.

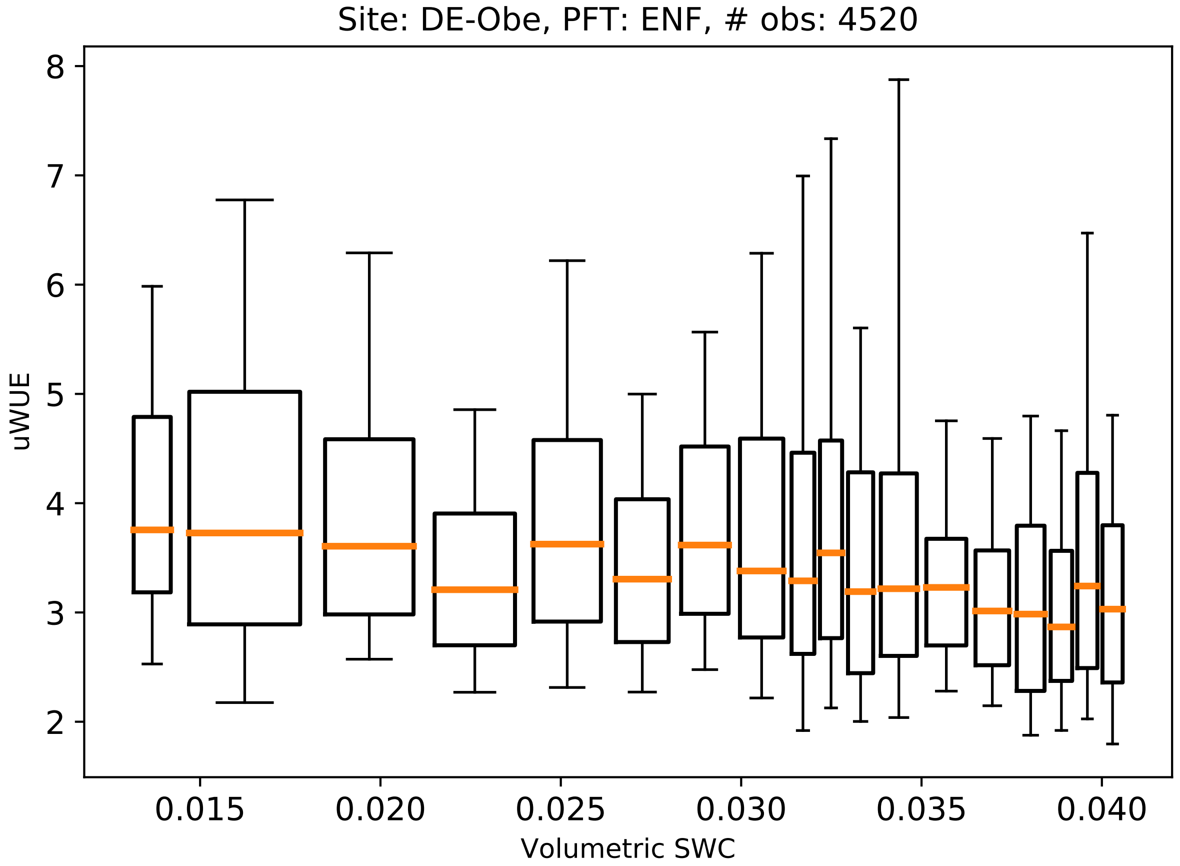

Figure S3. The relationship between uWUE and VPD at the FLUXNET site DE-Obe. Each box plot correspondes to 5% of the data. To aid visualization only the 0%-90% range of SWC bins are included.

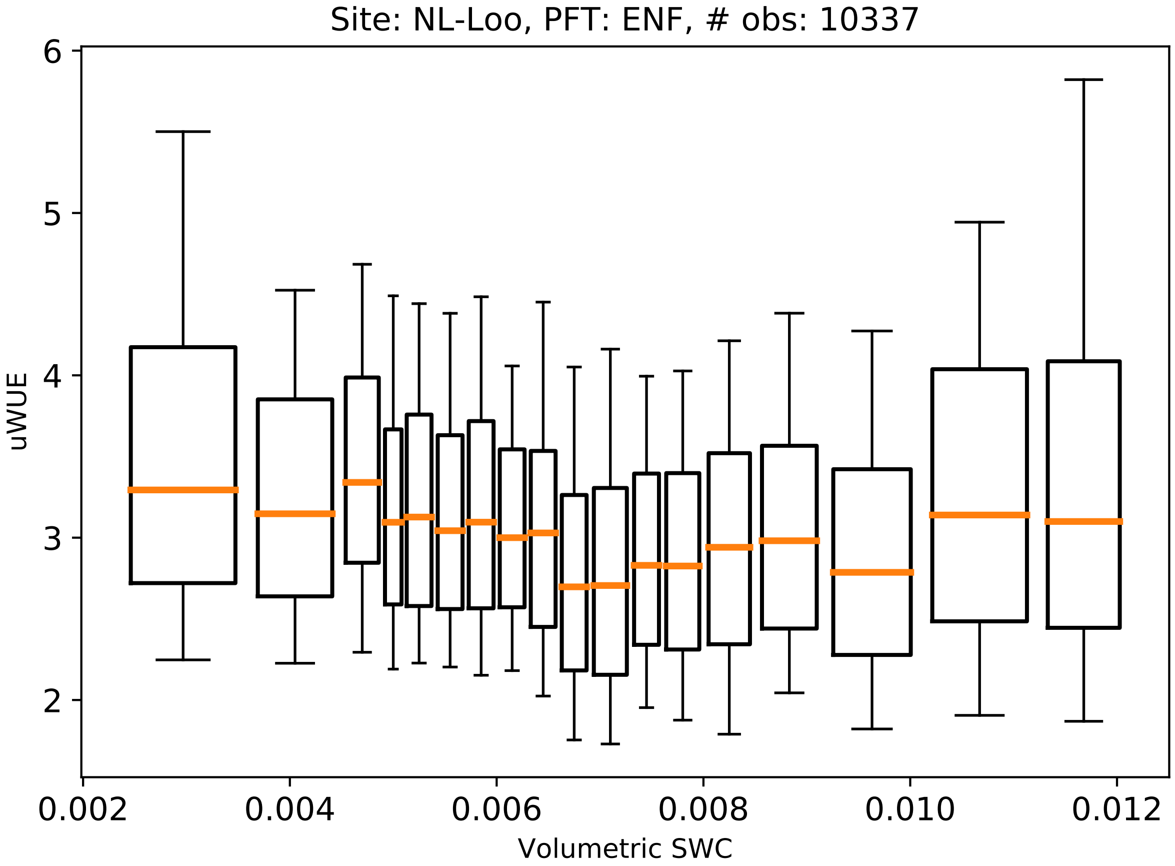

Figure S4. The relationship between uWUE and VPD at the FLUXNET site NL-Loo. Each box plot correspondes to 5% of the data. To aid visualization only the 0%-90% range of SWC bins are included.

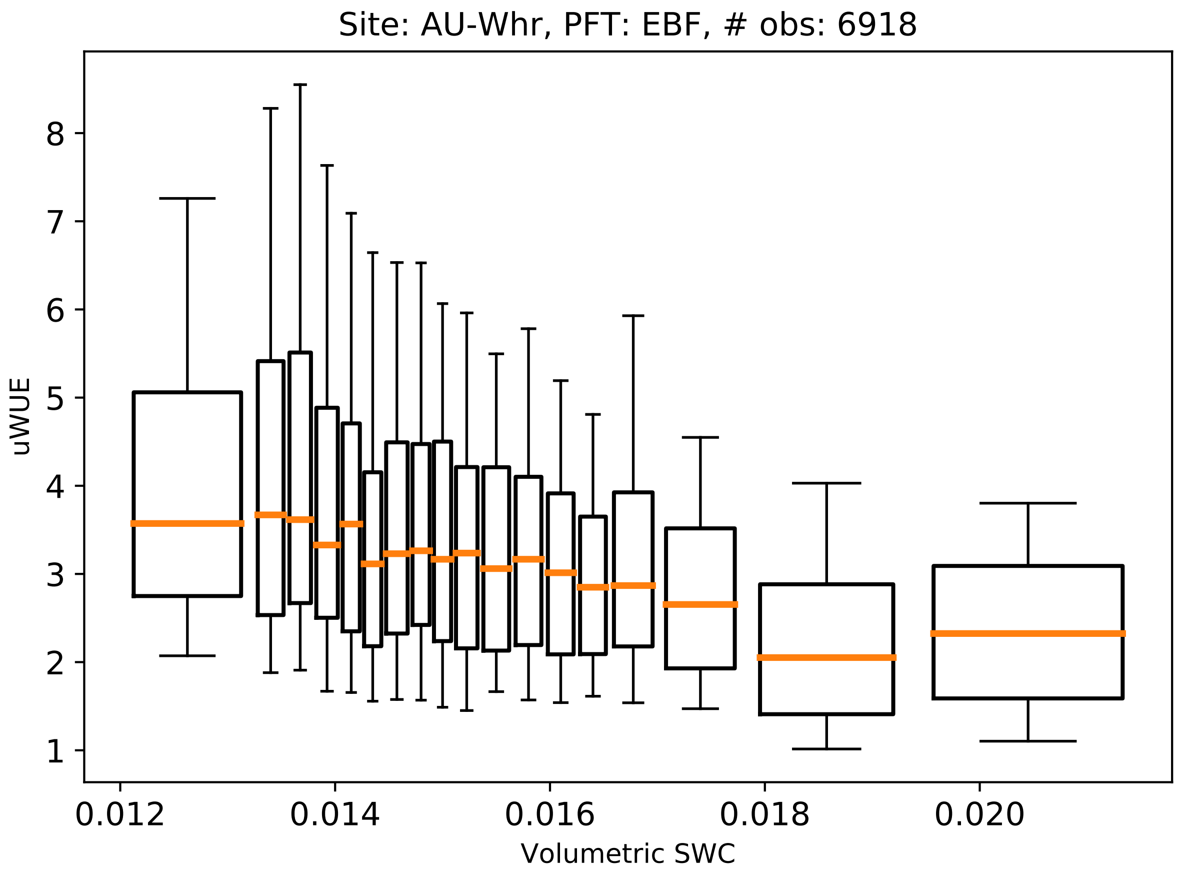

Figure S5. The relationship between uWUE and VPD at the FLUXNET site AU-Whr. Each box plot correspondes to 5% of the data. To aid visualization only the 0%-90% range of SWC bins are included.

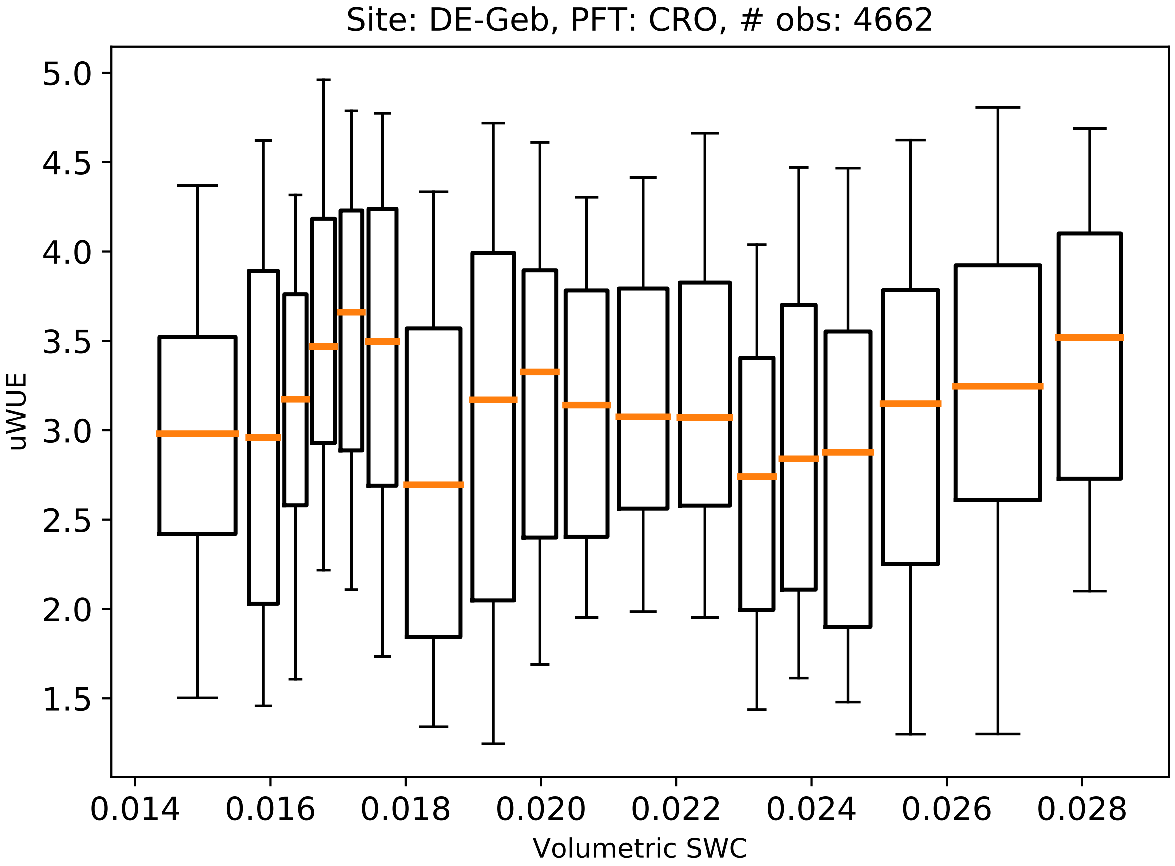

Figure S6. The relationship between uWUE and VPD at the FLUXNET site DE-Geb. Each box plot correspondes to 5% of the data. To aid visualization only the 0%-90% range of SWC bins are included.

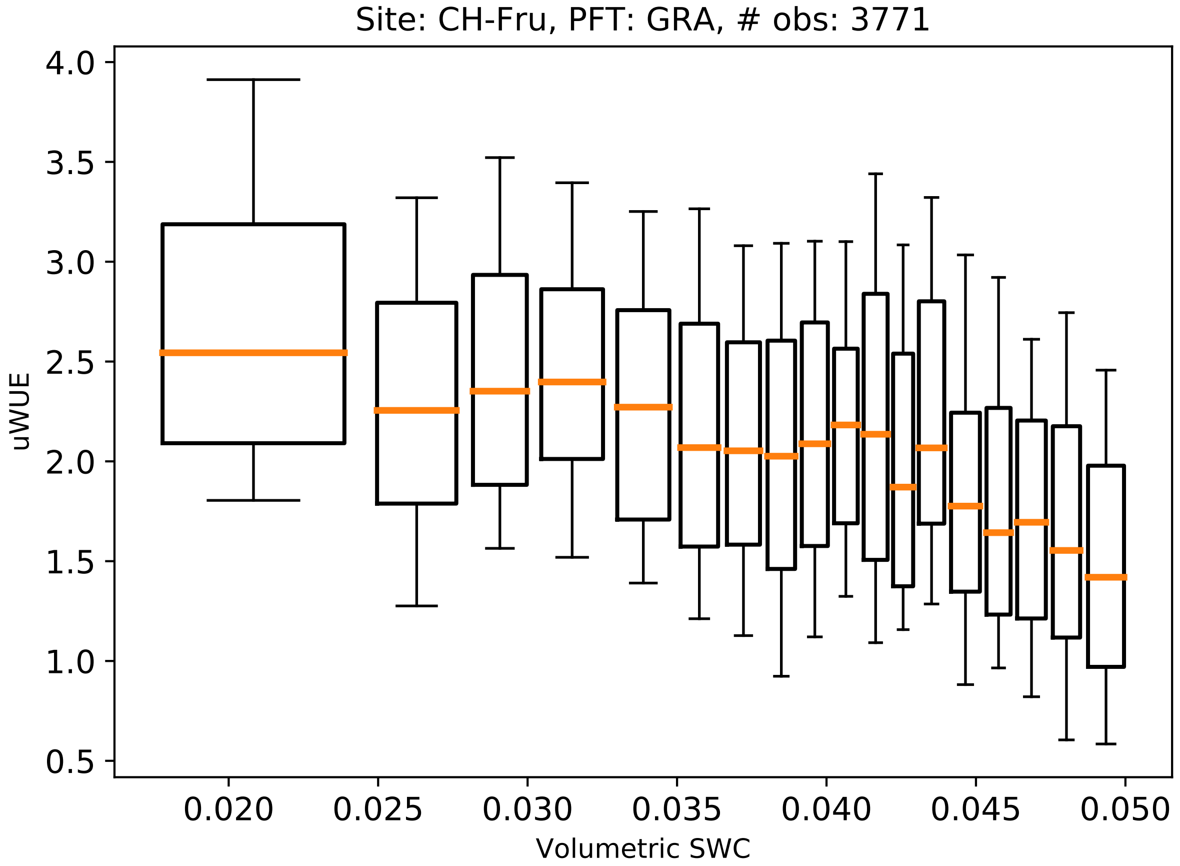

Figure S7. The relationship between uWUE and VPD at the FLUXNET site CH-Fru. Each box plot correspondes to 5% of the data. To aid visualization only the 0%-90% range of SWC bins are included.

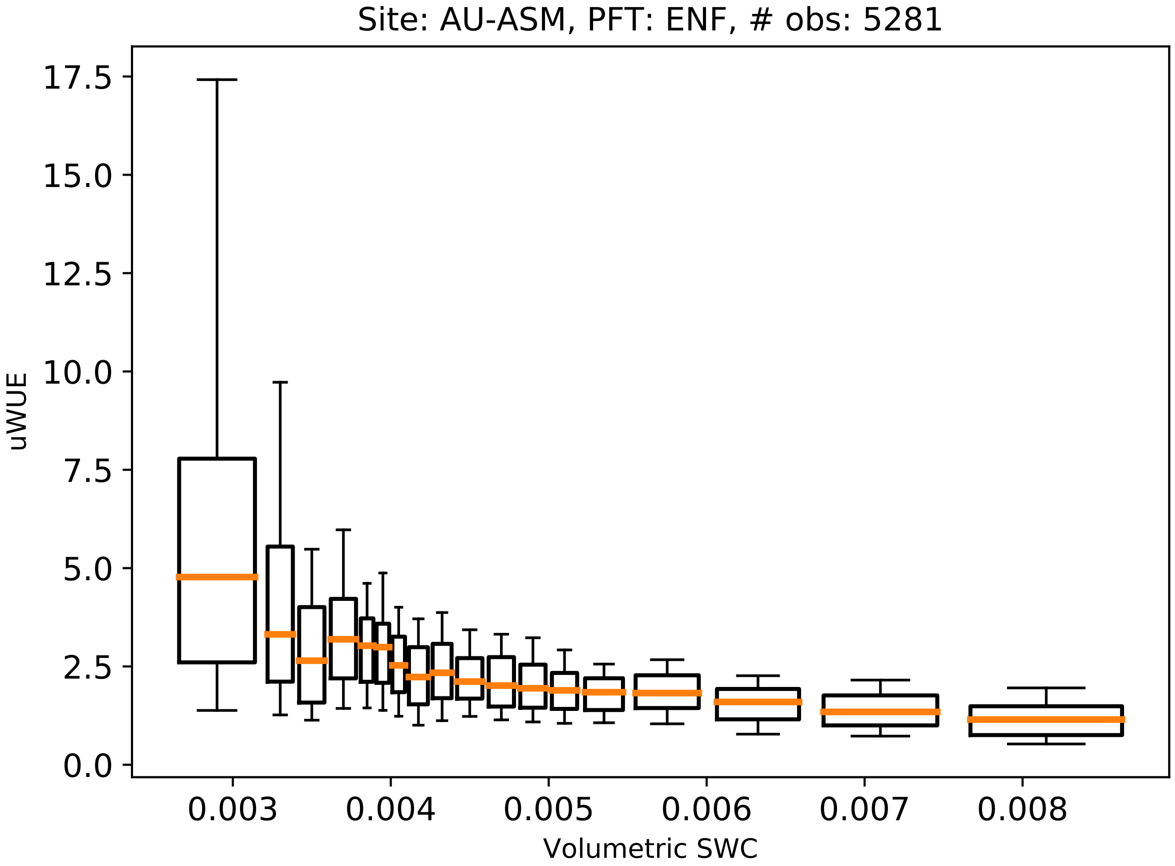

Figure S8. The relationship between uWUE and VPD at the FLUXNET site AU-ASM. Each box plot correspondes to 5% of the data. To aid visualization only the 0%-90% range of SWC bins are included.

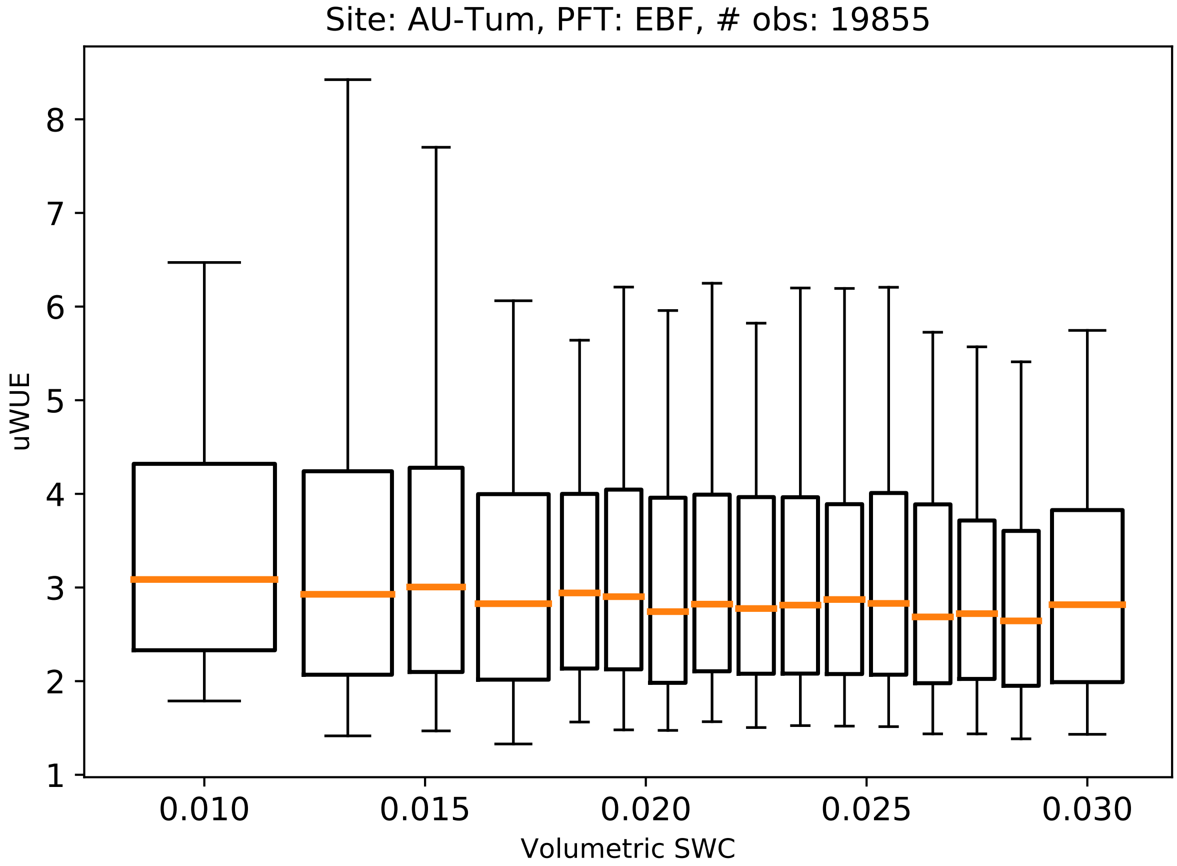

Figure S9. The relationship between uWUE and VPD at the FLUXNET site AU-Tum. Each box plot correspondes to 5% of the data. To aid visualization only the 0%-90% range of SWC bins are included.

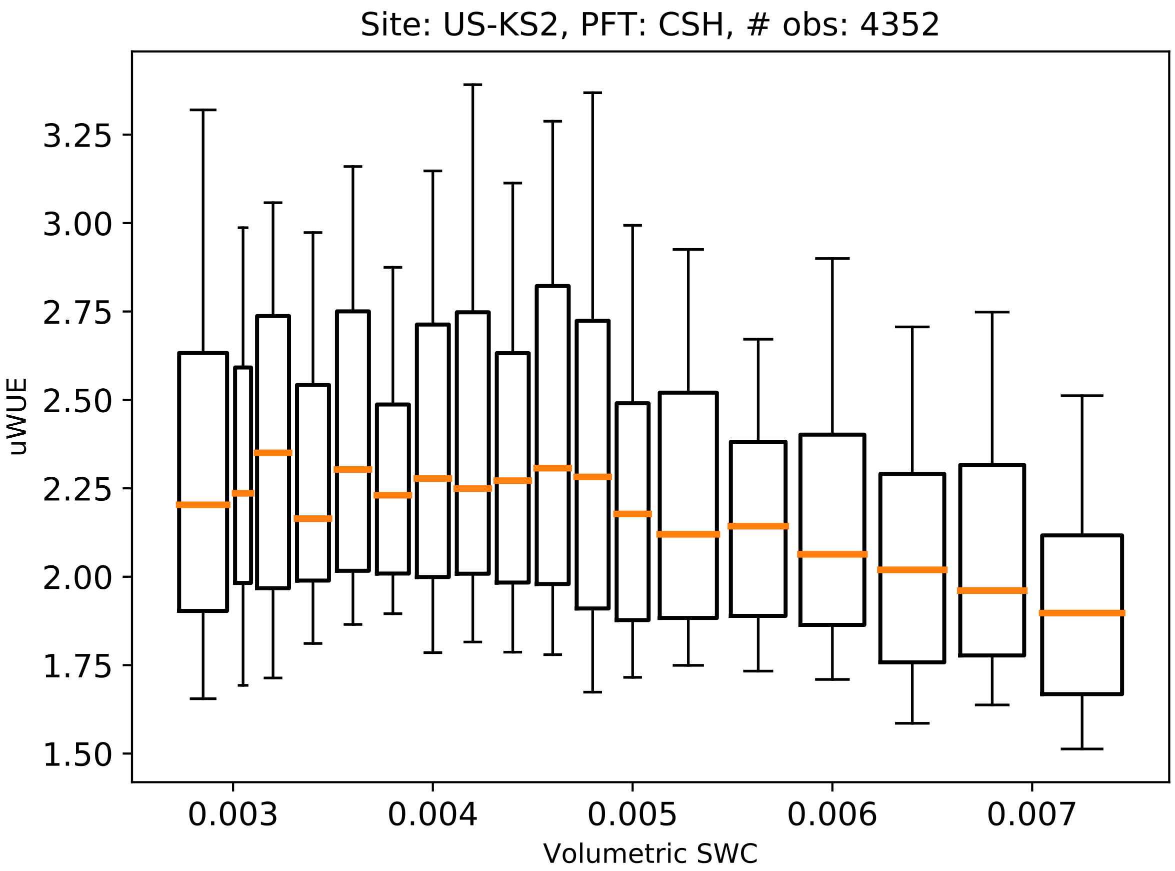

Figure S10. The relationship between uWUE and VPD at the FLUXNET site US-KS2. Each box plot correspondes to 5% of the data. To aid visualization only the 0%-90% range of SWC bins are included.

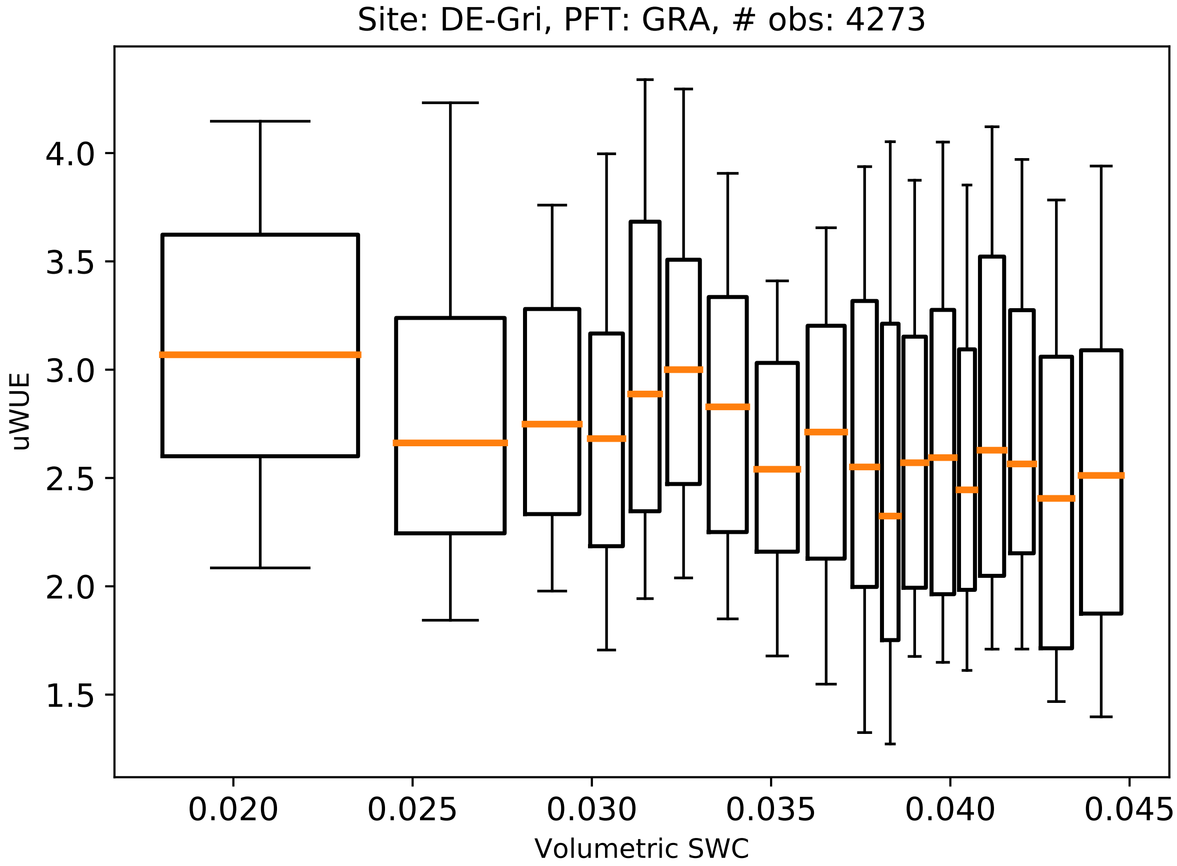

Figure S11. The relationship between uWUE and VPD at the FLUXNET site DE-Gri. Each box plot correspondes to 5% of the data. To aid visualization only the 0%-90% range of SWC bins are included.

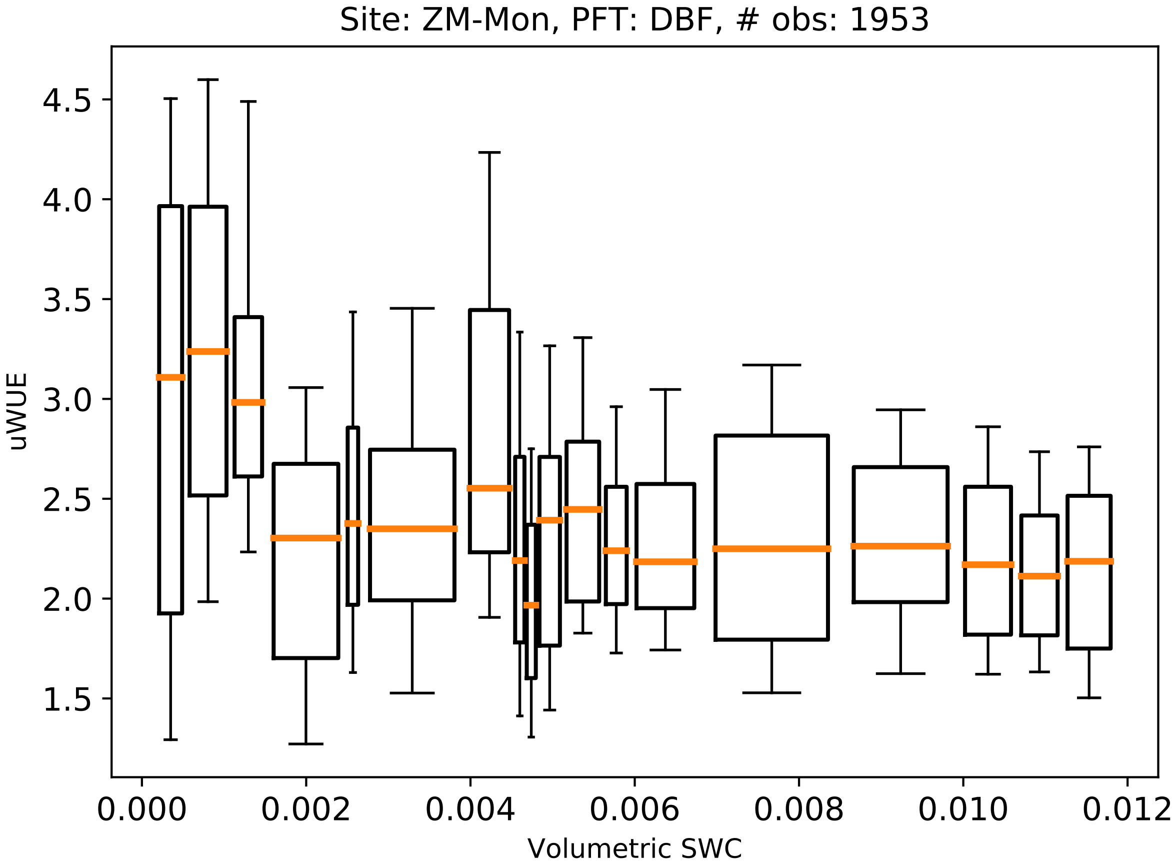

Figure S12. The relationship between uWUE and VPD at the FLUXNET site ZM-Mon. Each box plot correspondes to 5% of the data. To aid visualization only the 0%-90% range of SWC bins are included.

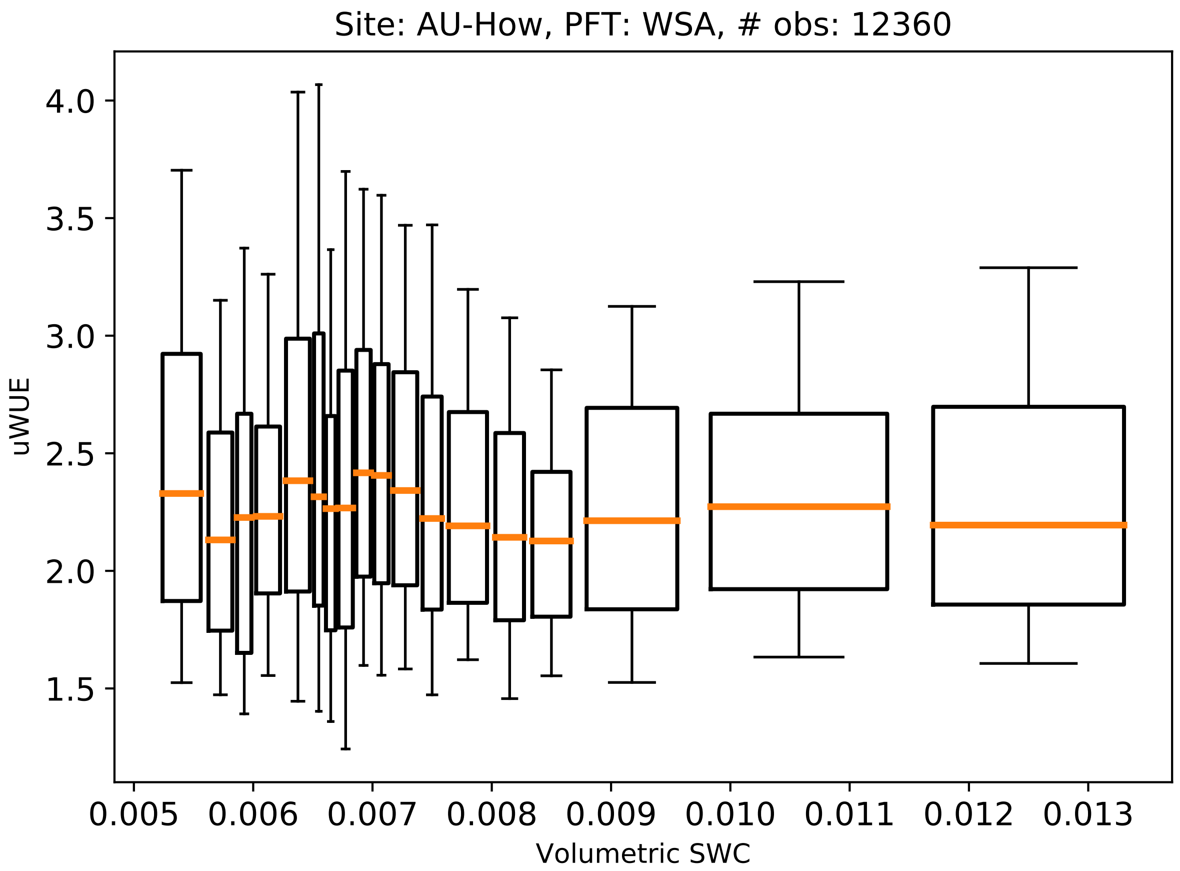

Figure S13. The relationship between uWUE and VPD at the FLUXNET site AU-How. Each box plot correspondes to 5% of the data. To aid visualization only the 0%-90% range of SWC bins are included.

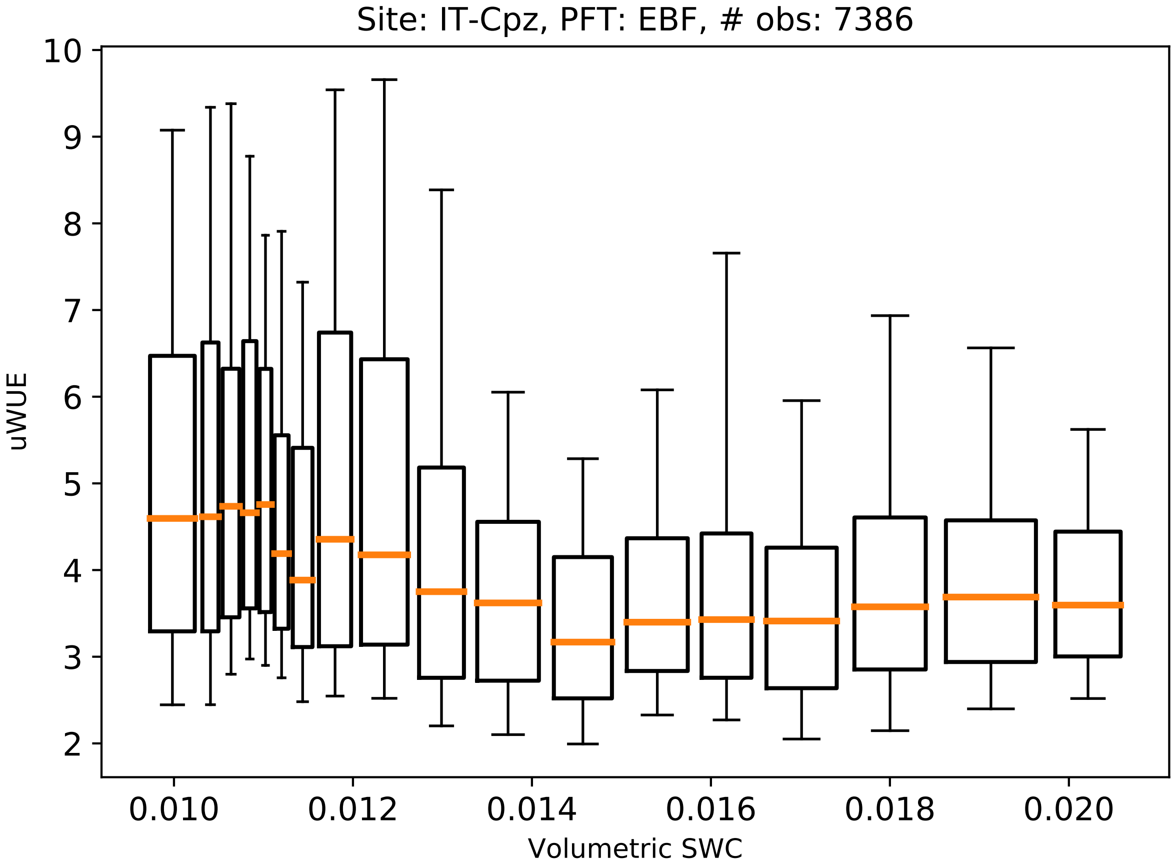

Figure S14. The relationship between uWUE and VPD at the FLUXNET site IT-Cpz. Each box plot correspondes to 5% of the data. To aid visualization only the 0%-90% range of SWC bins are included.

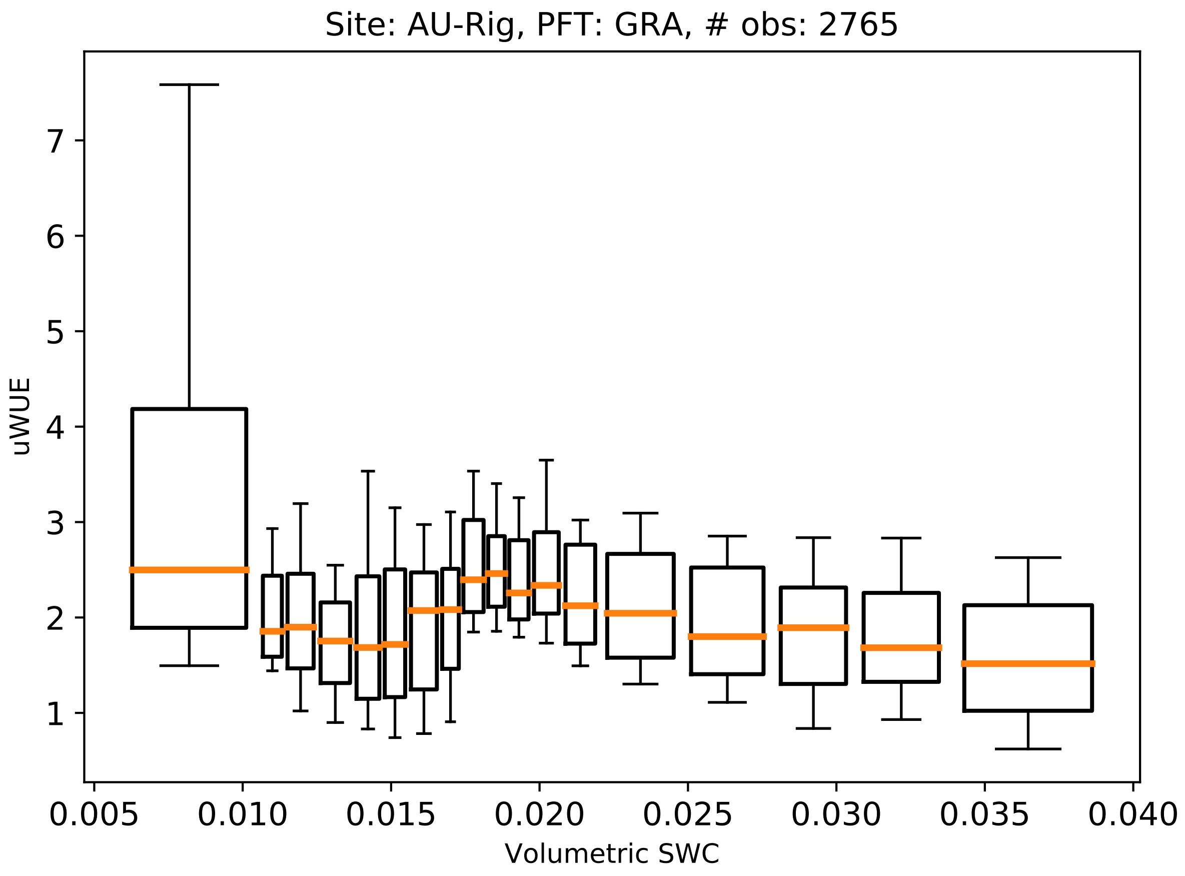

Figure S15. The relationship between uWUE and VPD at the FLUXNET site AU-Rig. Each box plot correspondes to 5% of the data. To aid visualization only the 0%-90% range of SWC bins are included.

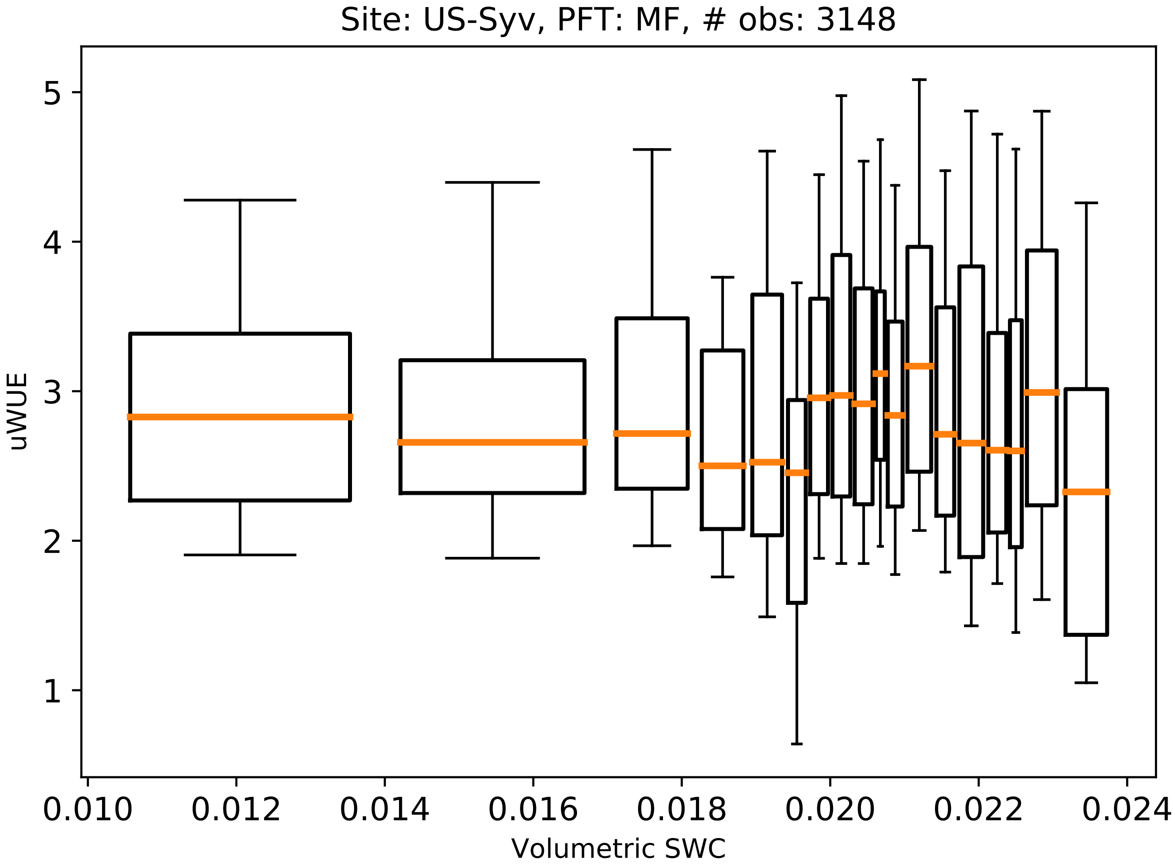

Figure S16. The relationship between uWUE and VPD at the FLUXNET site US-Syv. Each box plot correspondes to 5% of the data. To aid visualization only the 0%-90% range of SWC bins are included.

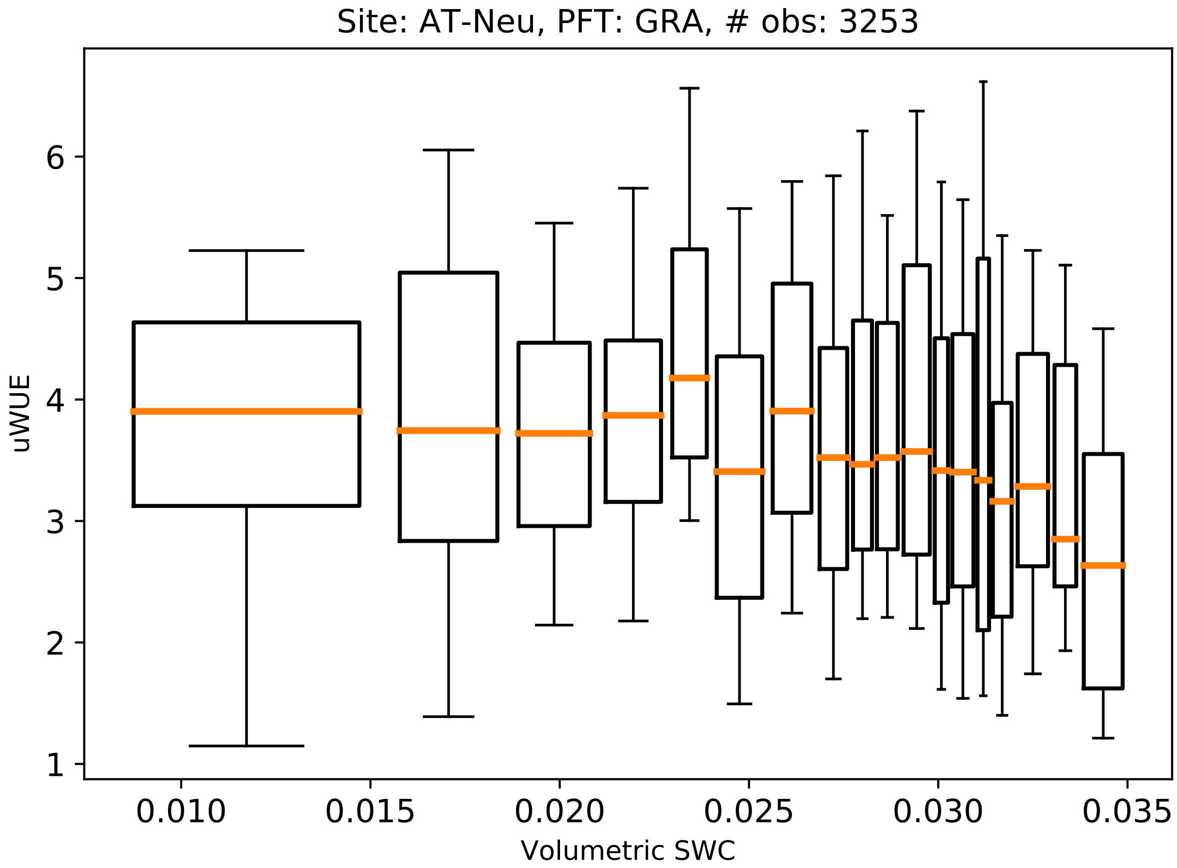

Figure S17. The relationship between uWUE and VPD at the FLUXNET site AT-Neu. Each box plot correspondes to 5% of the data. To aid visualization only the 0%-90% range of SWC bins are included.

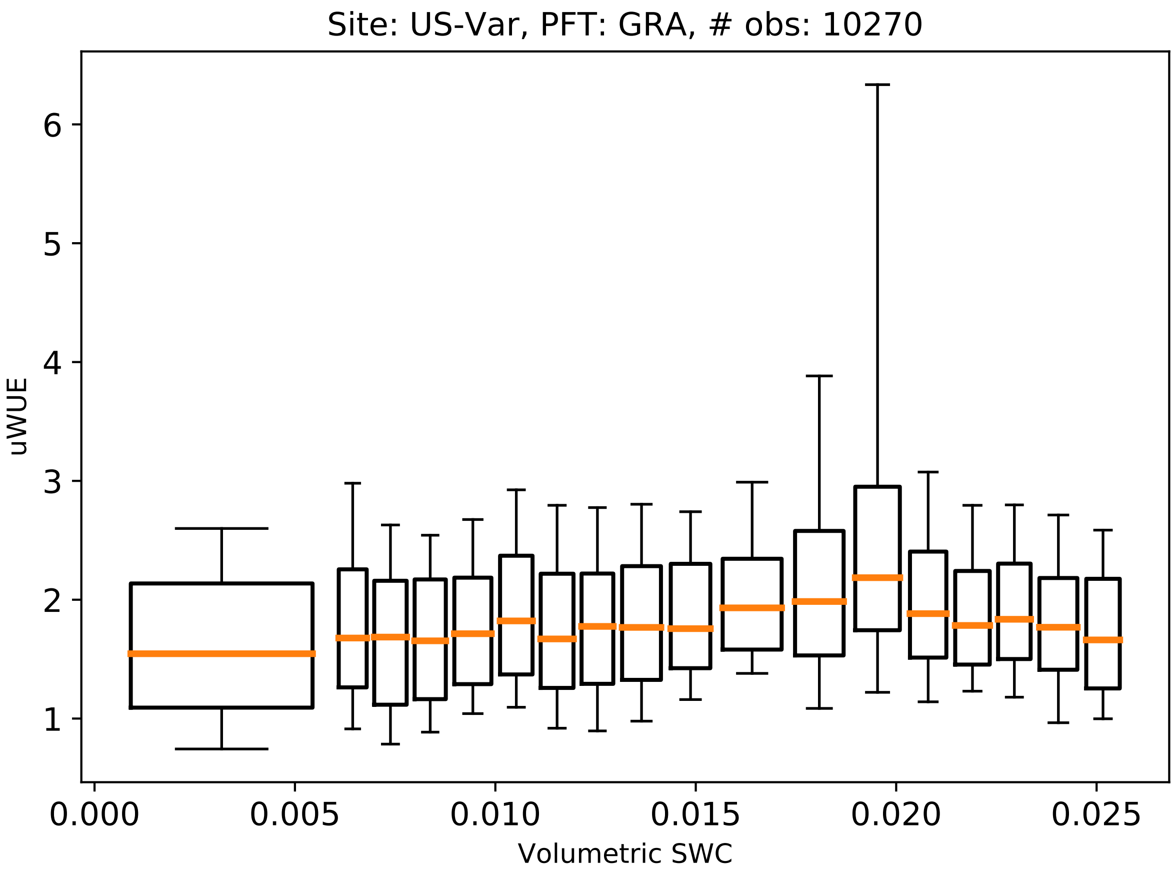

Figure S18. The relationship between uWUE and VPD at the FLUXNET site US-Var. Each box plot correspondes to 5% of the data. To aid visualization only the 0%-90% range of SWC bins are included.

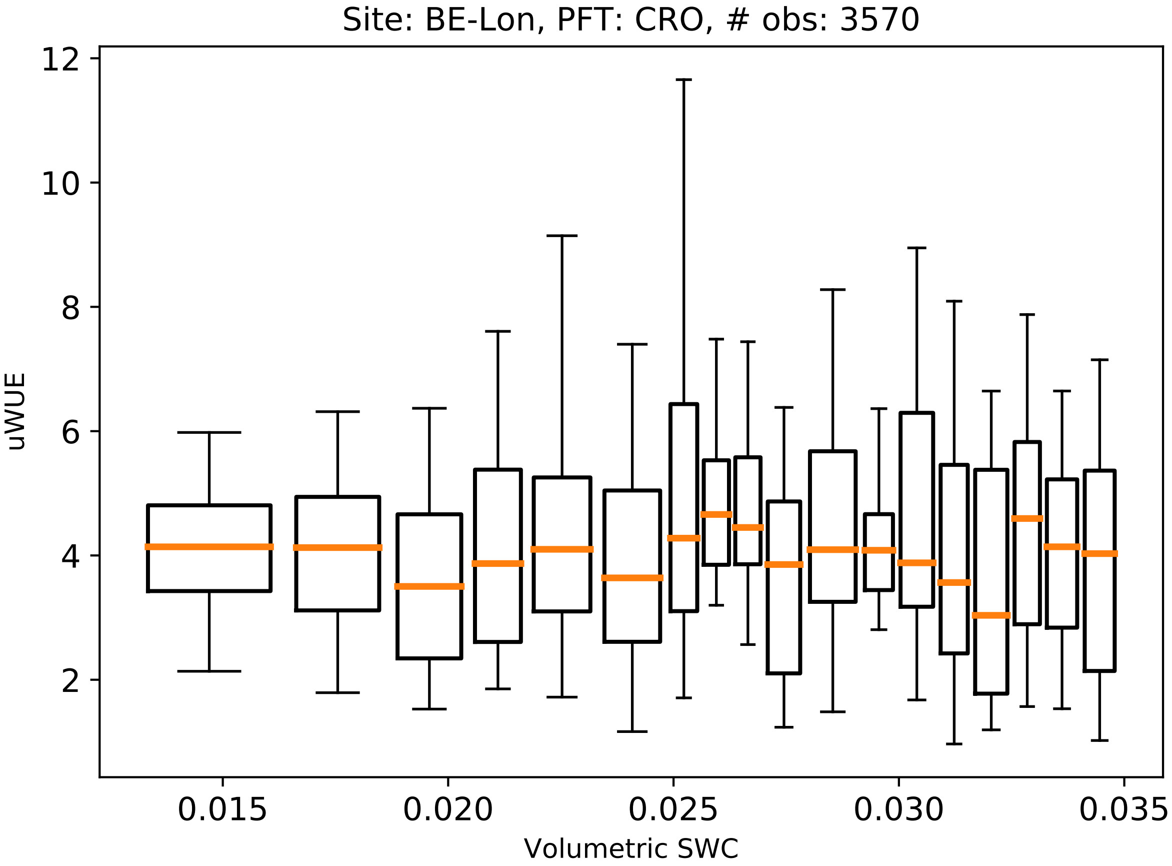

Figure S19. The relationship between uWUE and VPD at the FLUXNET site BE-Lon. Each box plot correspondes to 5% of the data. To aid visualization only the 0%-90% range of SWC bins are included.

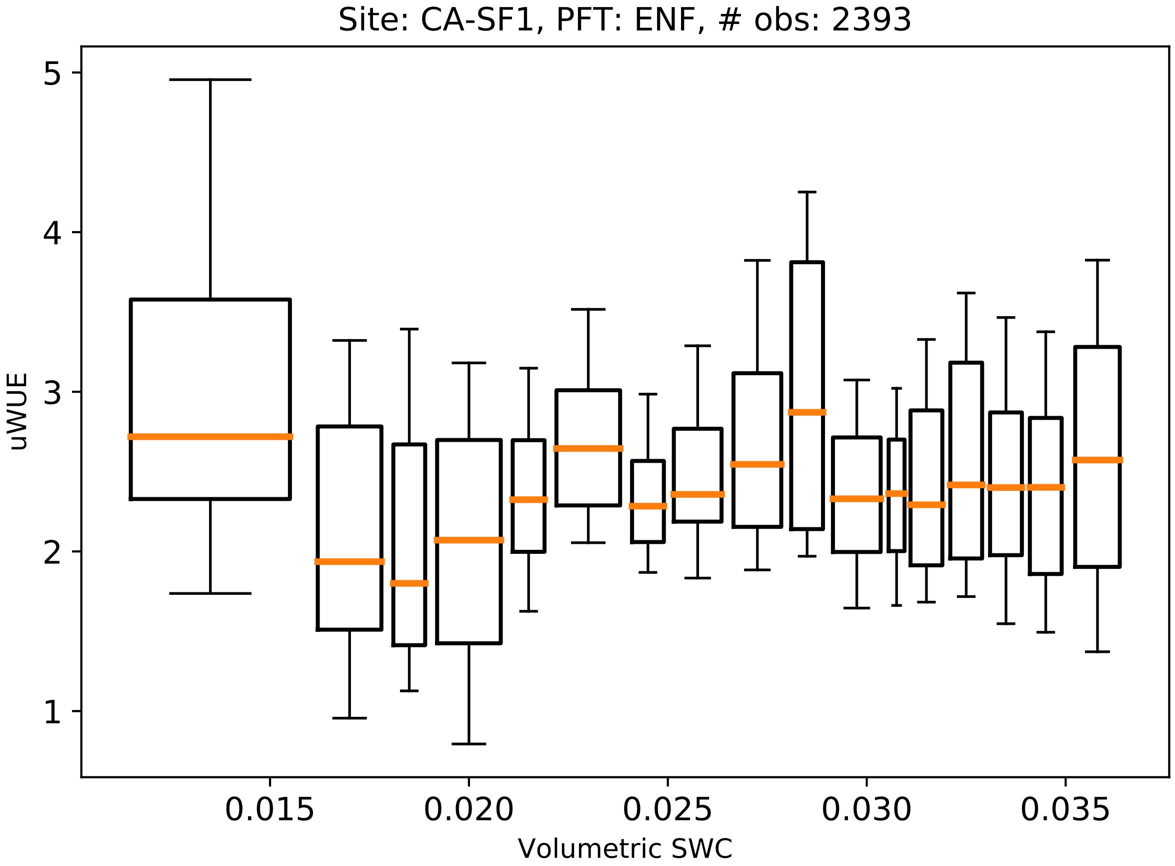

Figure S20. The relationship between uWUE and VPD at the FLUXNET site CA-SF1. Each box plot correspondes to 5% of the data. To aid visualization only the 0%-90% range of SWC bins are included.

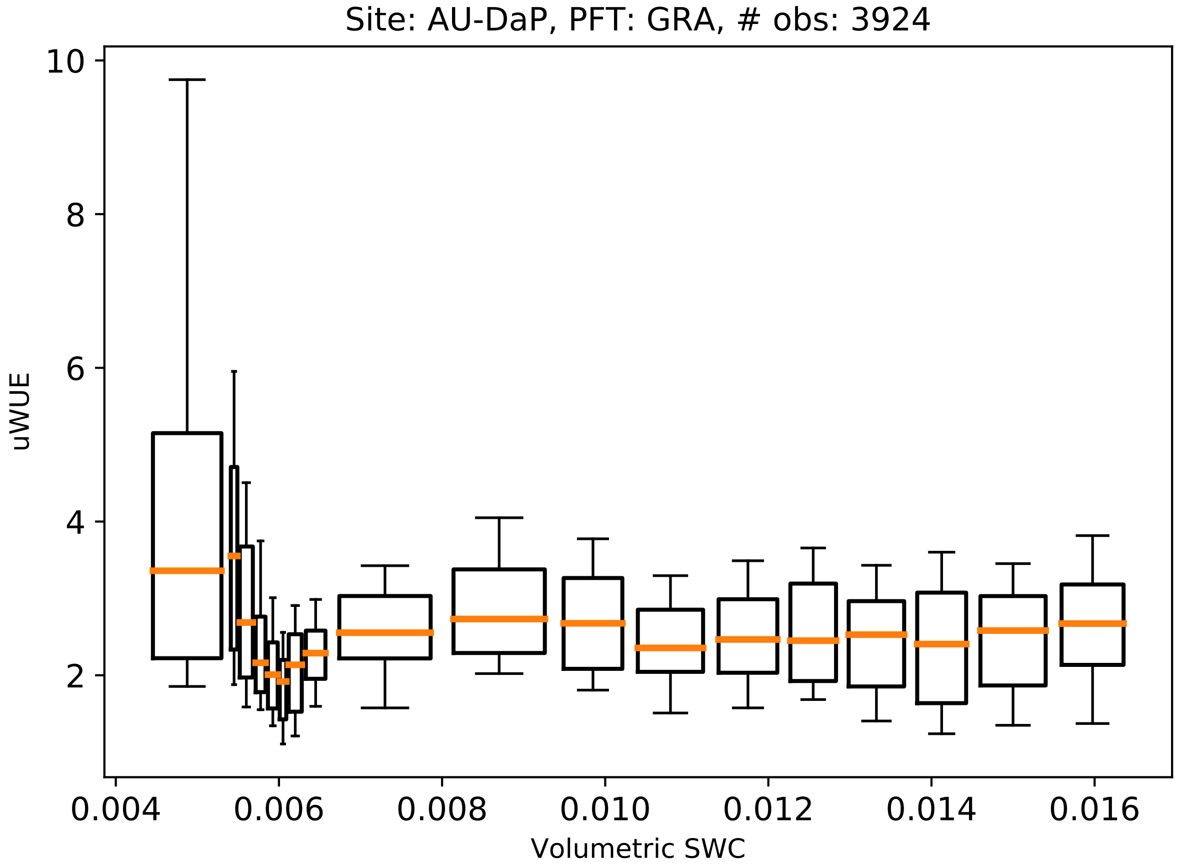

Figure S21. The relationship between uWUE and VPD at the FLUXNET site AU-DaP. Each box plot correspondes to 5% of the data. To aid visualization only the 0%-90% range of SWC bins are included.

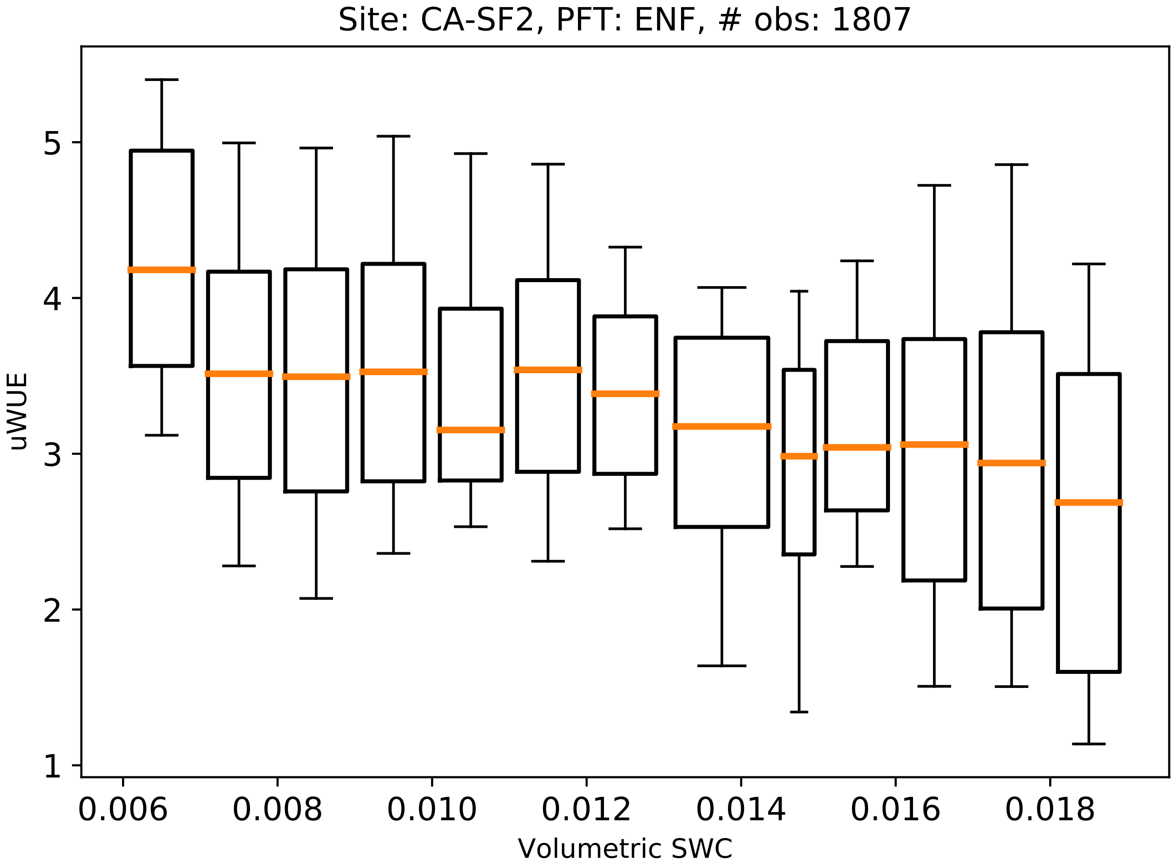

Figure S22. The relationship between uWUE and VPD at the FLUXNET site CA-SF2. Each box plot correspondes to 5% of the data. To aid visualization only the 0%-90% range of SWC bins are included.

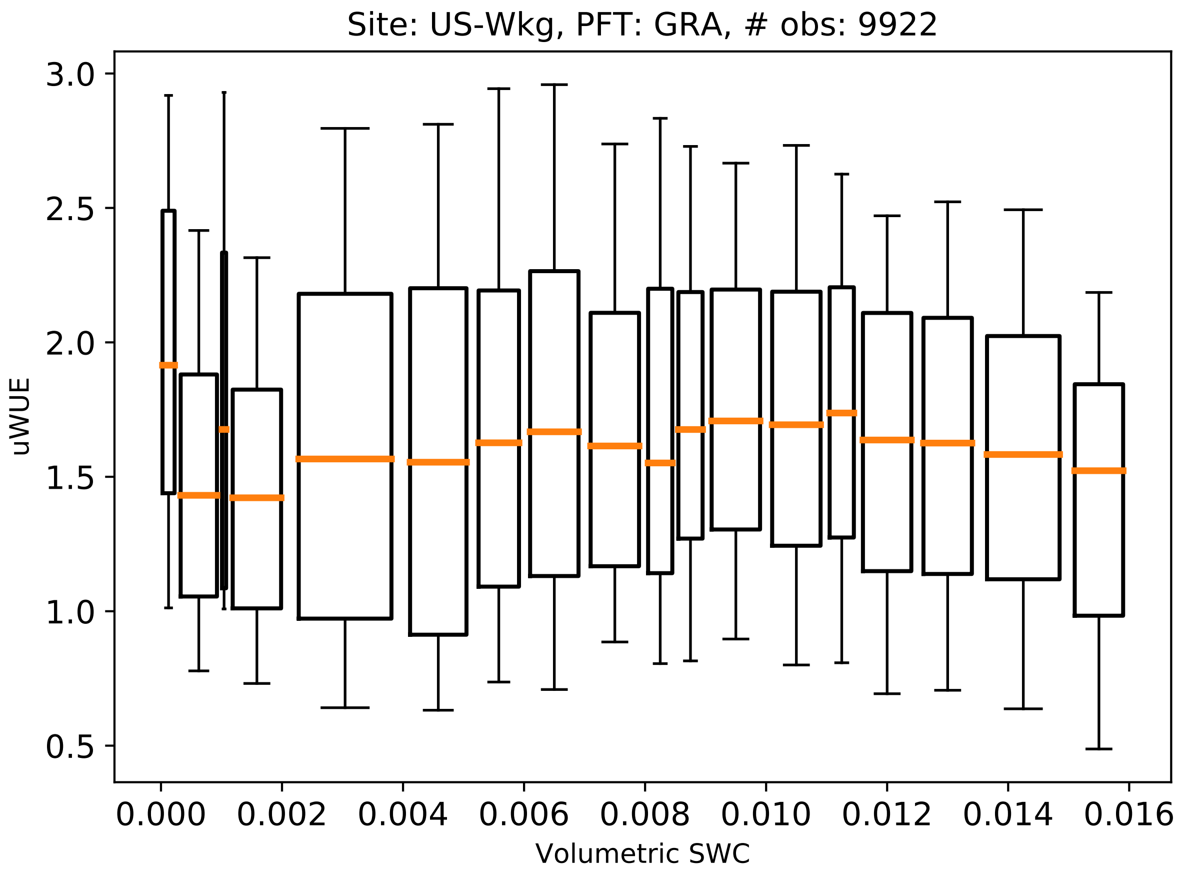

Figure S23. The relationship between uWUE and VPD at the FLUXNET site US-Wkg. Each box plot correspondes to 5% of the data. To aid visualization only the 0%-90% range of SWC bins are included.

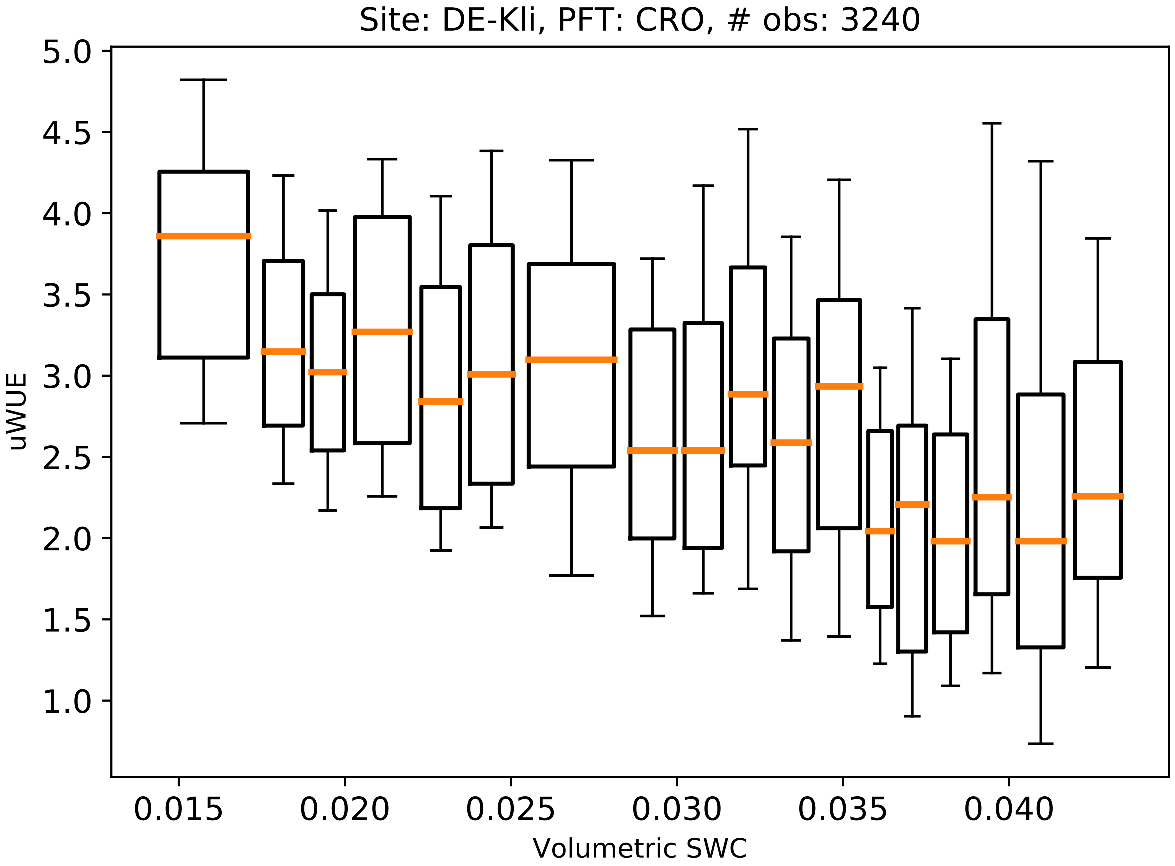

Figure S24. The relationship between uWUE and VPD at the FLUXNET site DE-Kli. Each box plot correspondes to 5% of the data. To aid visualization only the 0%-90% range of SWC bins are included.

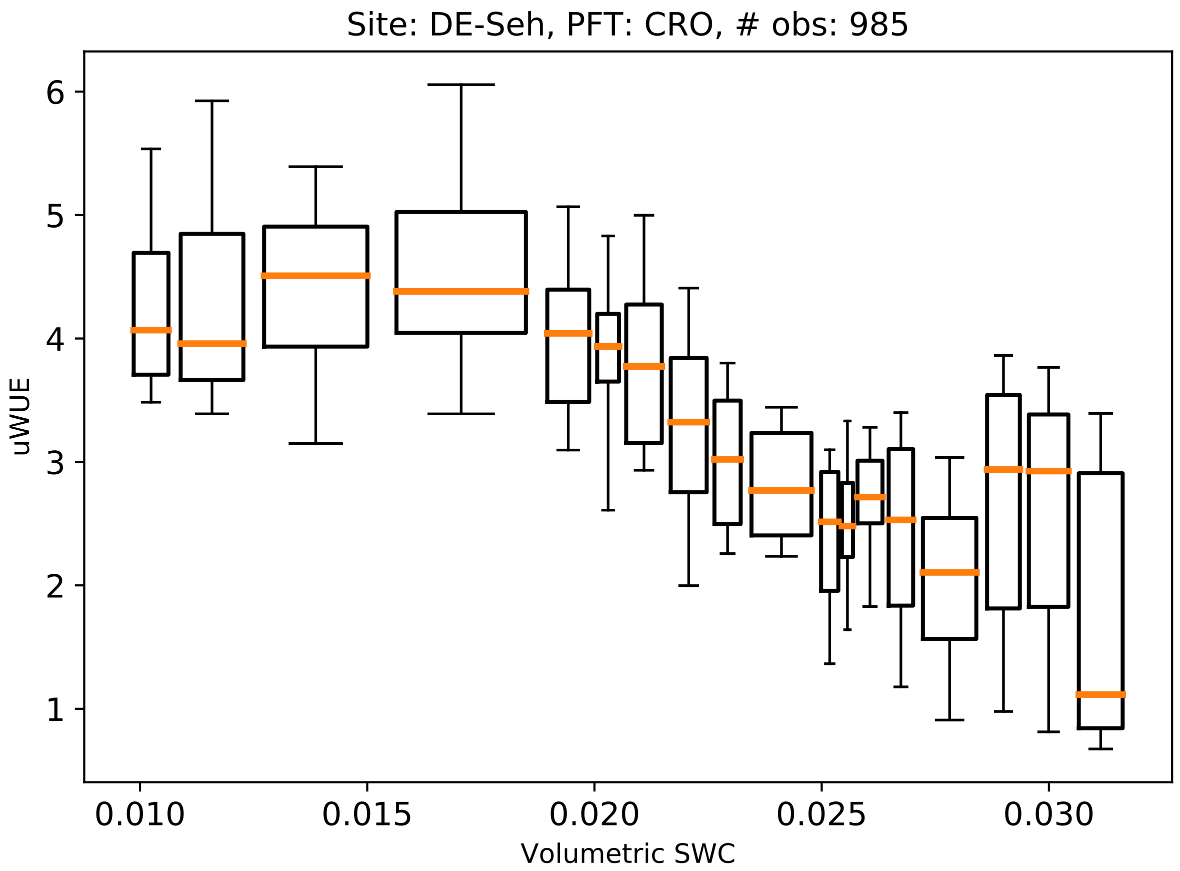

Figure S25. The relationship between uWUE and VPD at the FLUXNET site DE-Seh. Each box plot correspondes to 5% of the data. To aid visualization only the 0%-90% range of SWC bins are included.

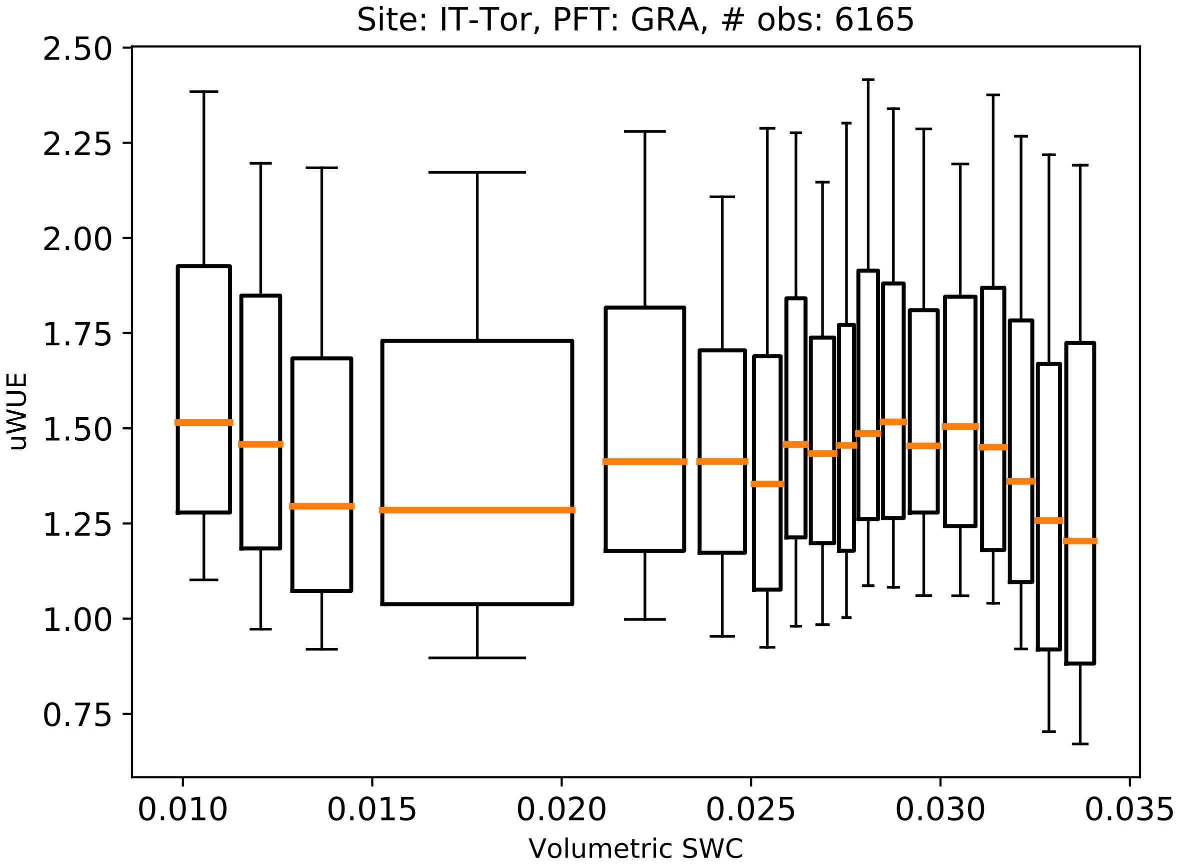

Figure S26. The relationship between uWUE and VPD at the FLUXNET site IT-Tor. Each box plot correspondes to 5% of the data. To aid visualization only the 0%-90% range of SWC bins are included.

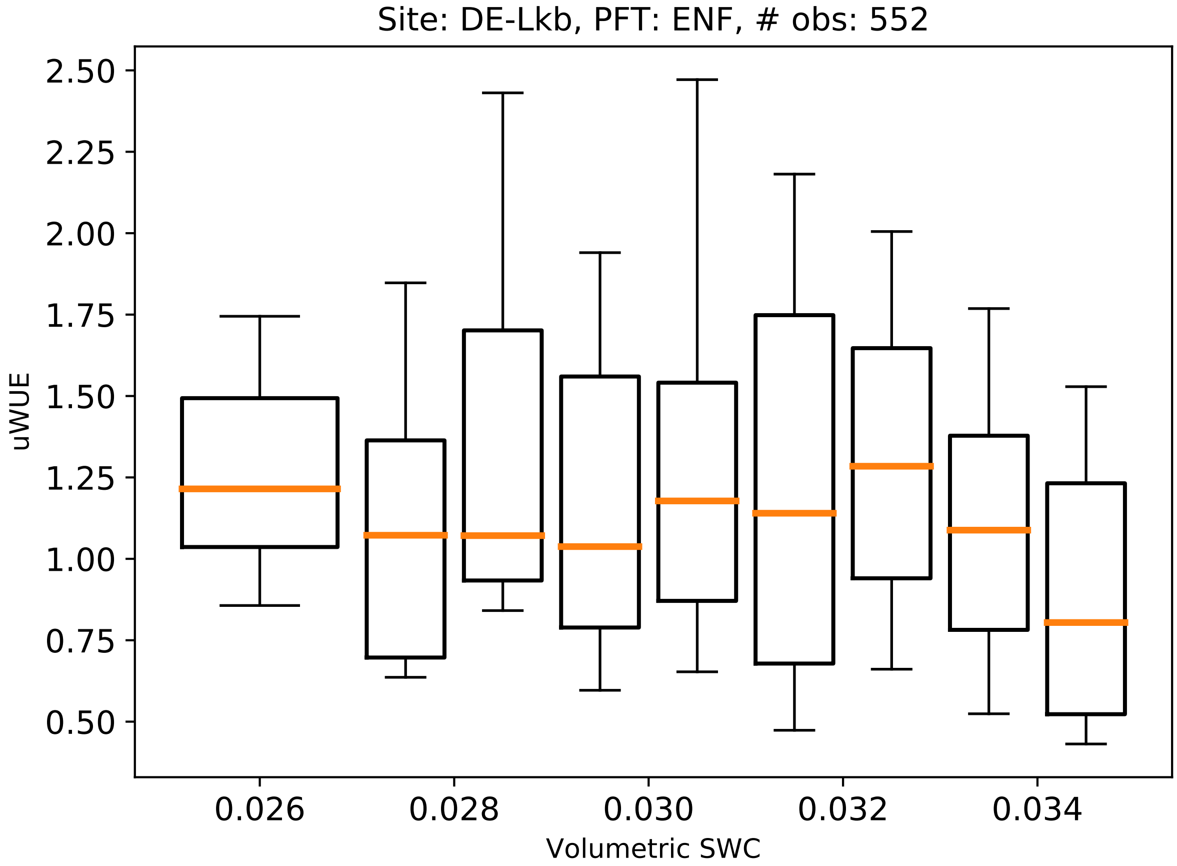

Figure S27. The relationship between uWUE and VPD at the FLUXNET site DE-Lkb. Each box plot correspondes to 5% of the data. To aid visualization only the 0%-90% range of SWC bins are included.

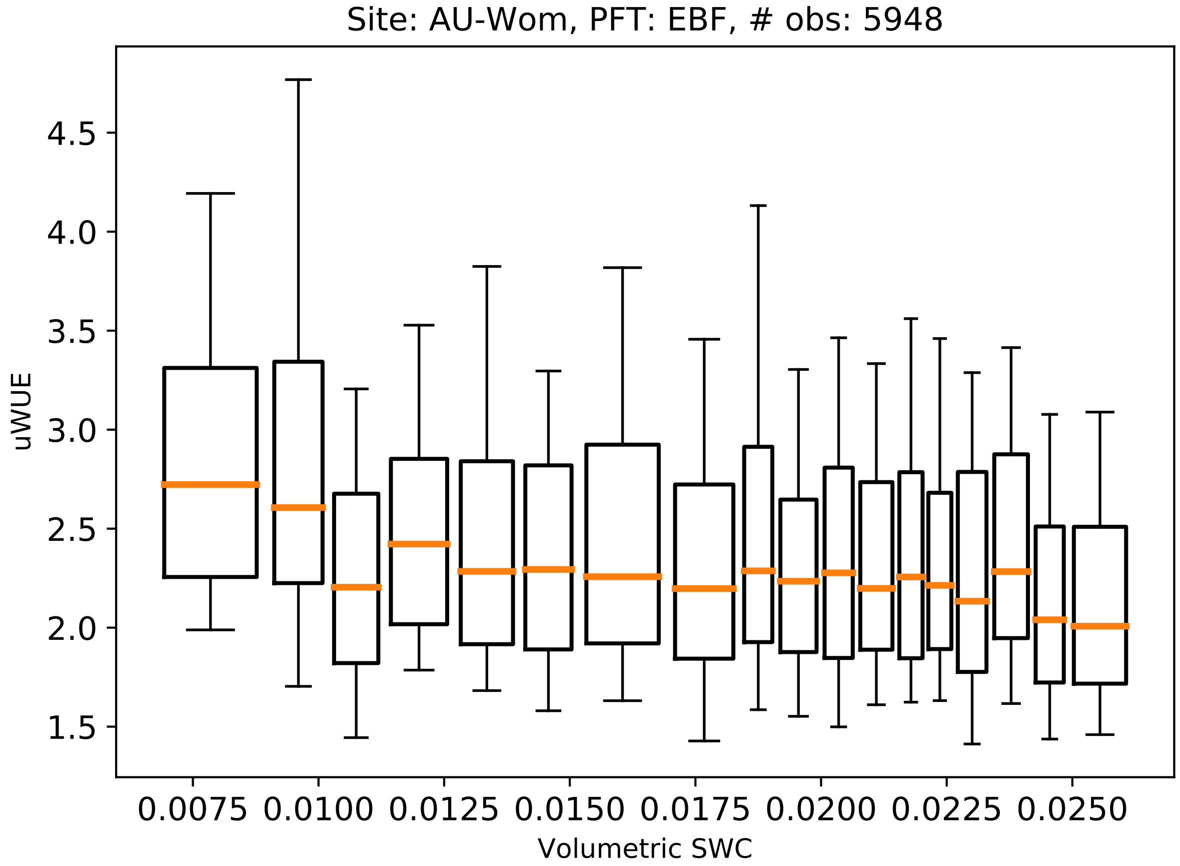

Figure S28. The relationship between uWUE and VPD at the FLUXNET site AU-Wom. Each box plot correspondes to 5% of the data. To aid visualization only the 0%-90% range of SWC bins are included.

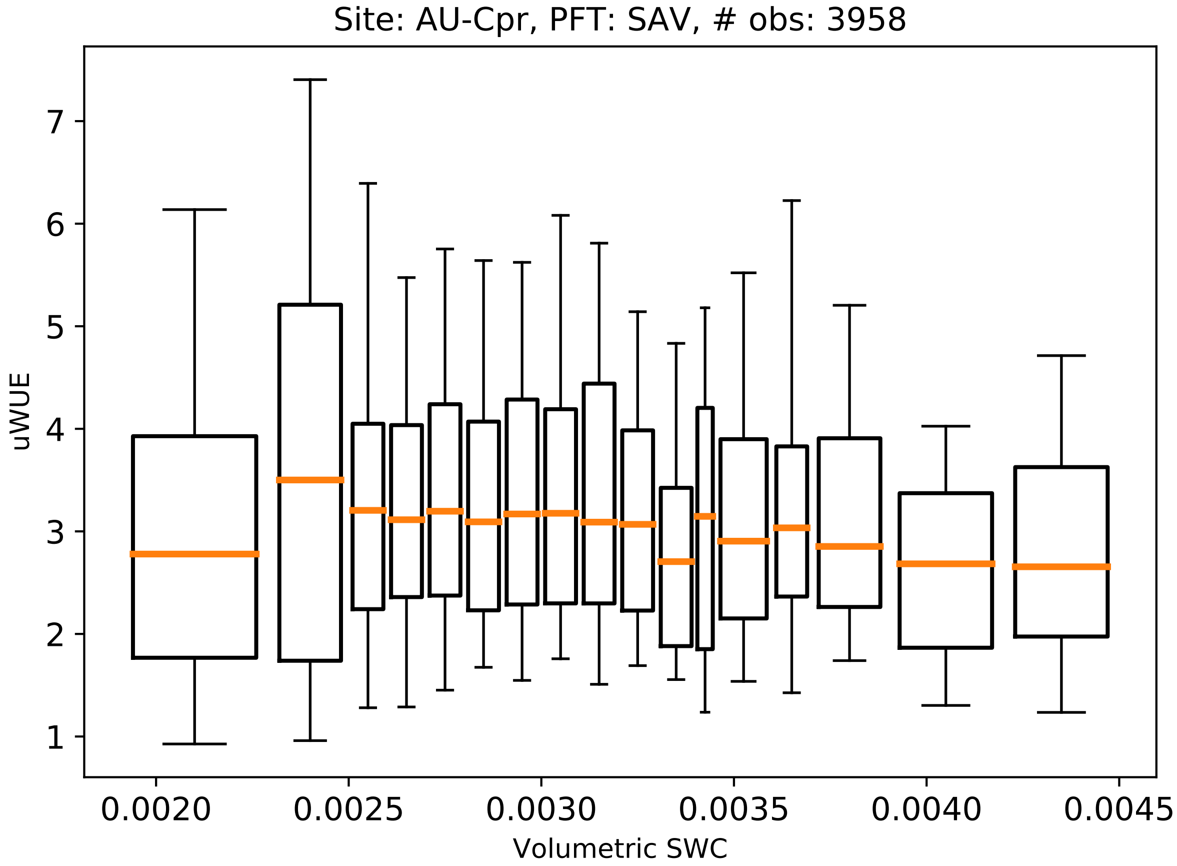

Figure S29. The relationship between uWUE and VPD at the FLUXNET site AU-Cpr. Each box plot correspondes to 5% of the data. To aid visualization only the 0%-90% range of SWC bins are included.

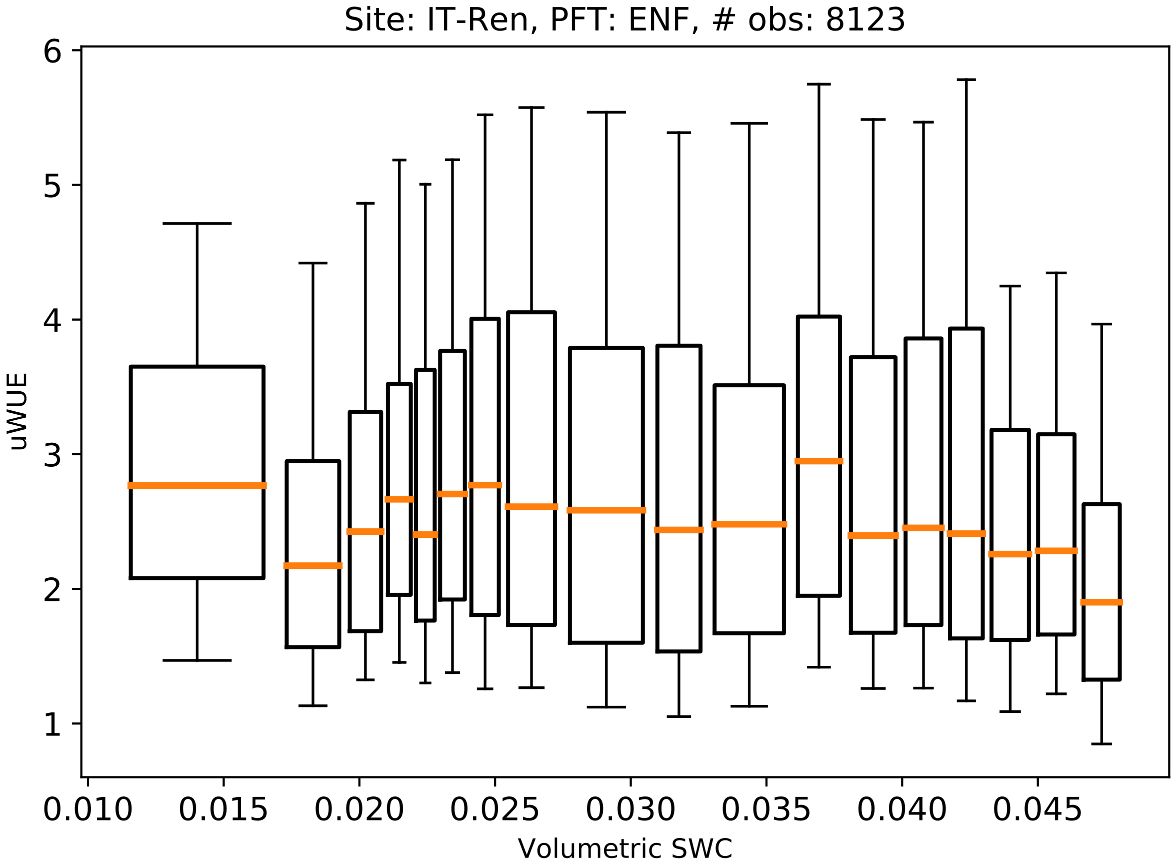

Figure S30. The relationship between uWUE and VPD at the FLUXNET site IT-Ren. Each box plot correspondes to 5% of the data. To aid visualization only the 0%-90% range of SWC bins are included.

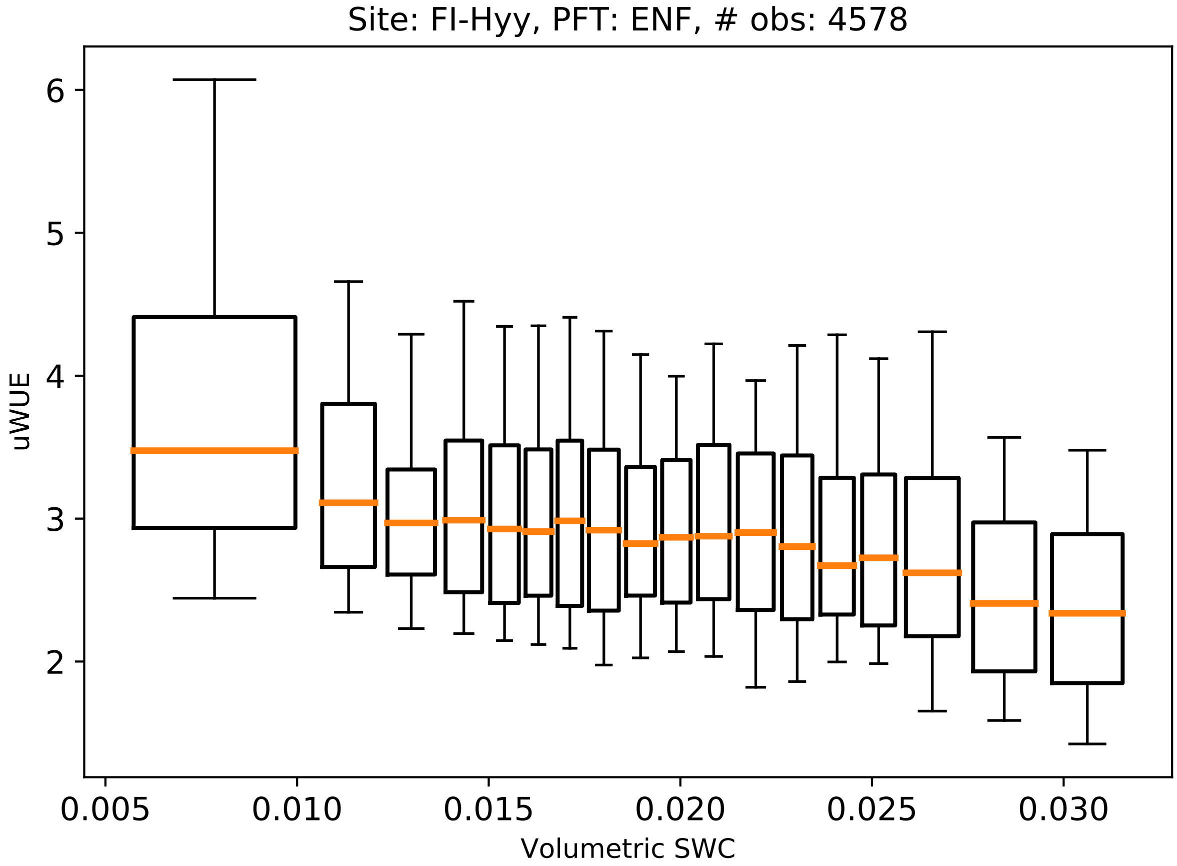

Figure S31. The relationship between uWUE and VPD at the FLUXNET site FI-Hyy. Each box plot correspondes to 5% of the data. To aid visualization only the 0%-90% range of SWC bins are included.

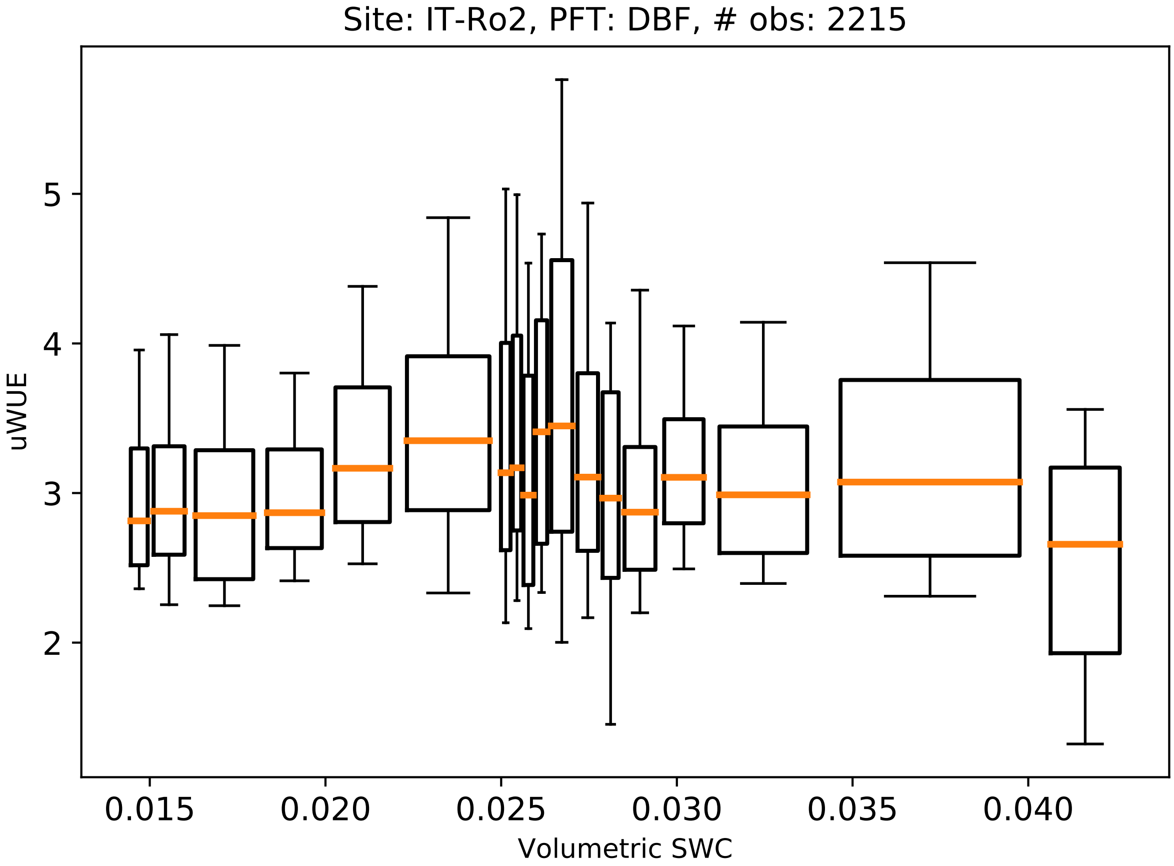

Figure S32. The relationship between uWUE and VPD at the FLUXNET site IT-Ro2. Each box plot correspondes to 5% of the data. To aid visualization only the 0%-90% range of SWC bins are included.

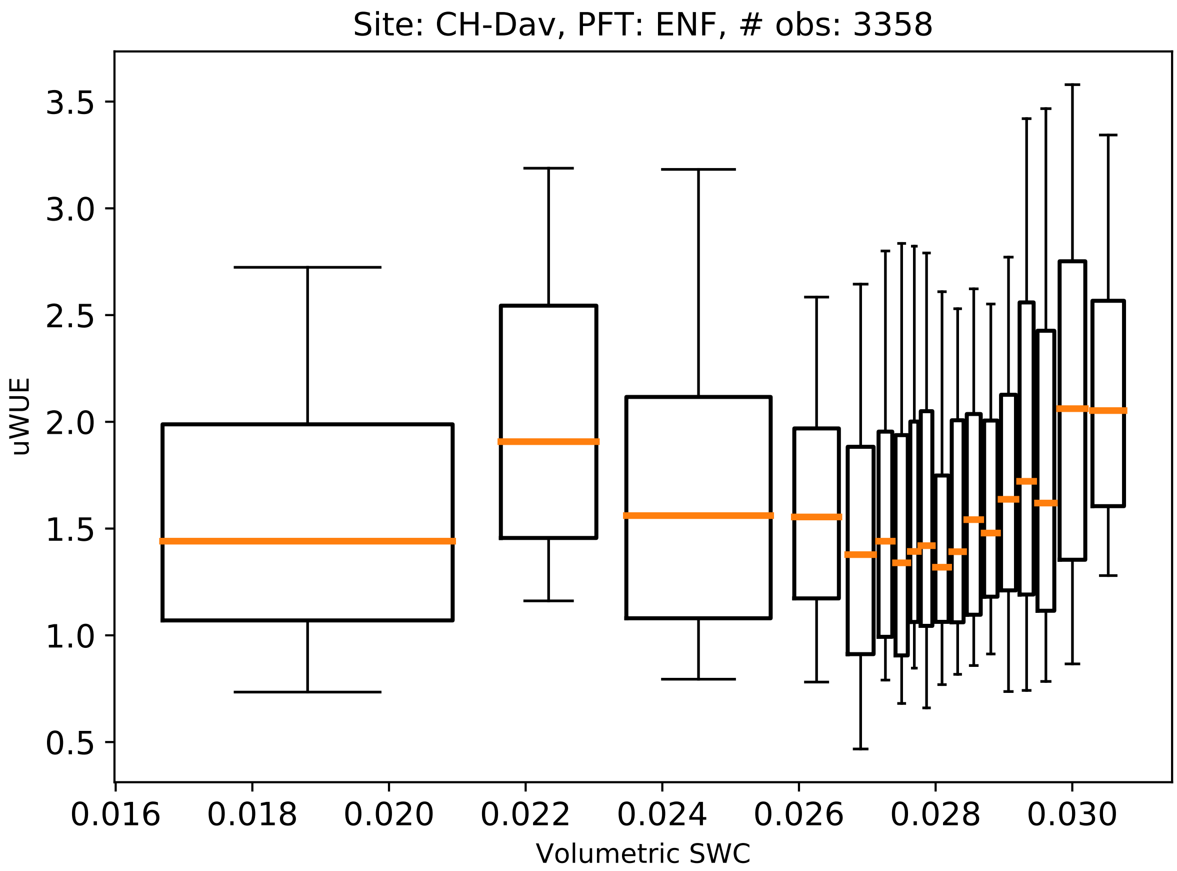

Figure S33. The relationship between uWUE and VPD at the FLUXNET site CH-Dav. Each box plot correspondes to 5% of the data. To aid visualization only the 0%-90% range of SWC bins are included.

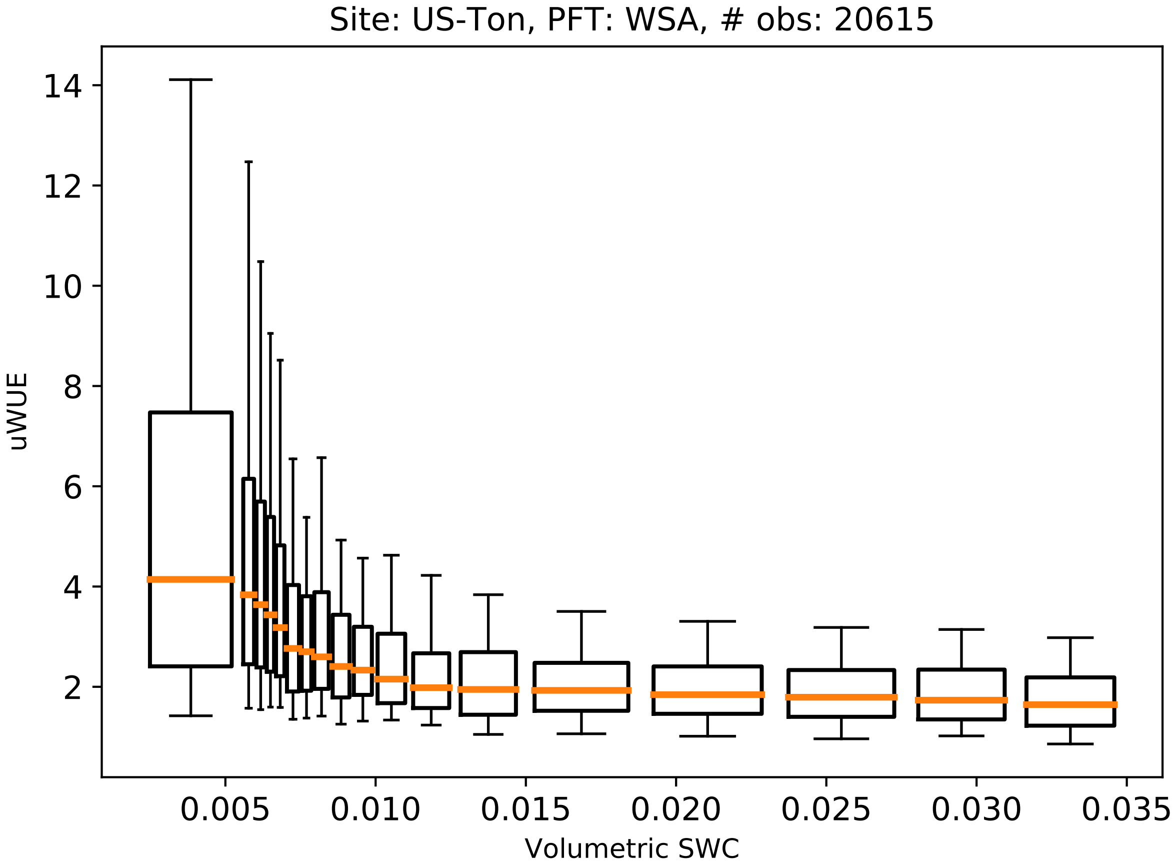

Figure S34. The relationship between uWUE and VPD at the FLUXNET site US-Ton. Each box plot correspondes to 5% of the data. To aid visualization only the 0%-90% range of SWC bins are included.

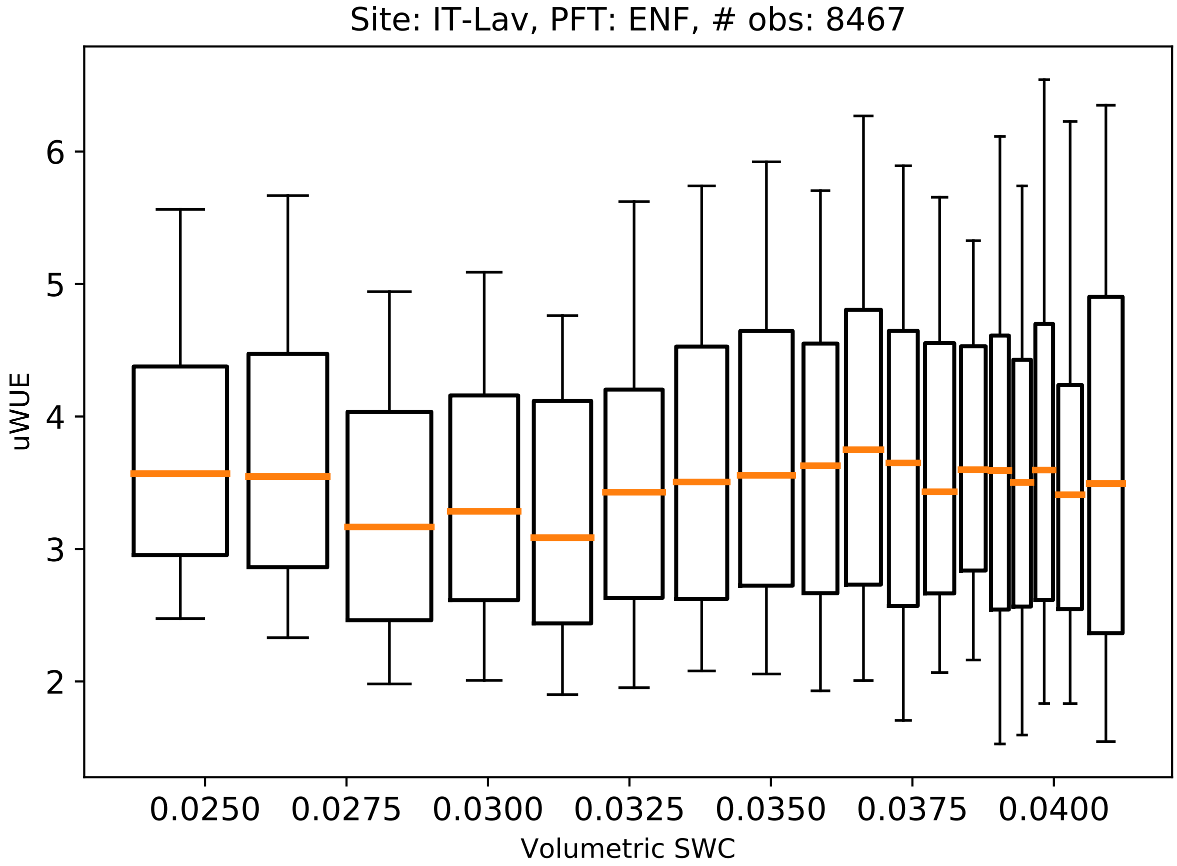

Figure S35. The relationship between uWUE and VPD at the FLUXNET site IT-Lav. Each box plot correspondes to 5% of the data. To aid visualization only the 0%-90% range of SWC bins are included.

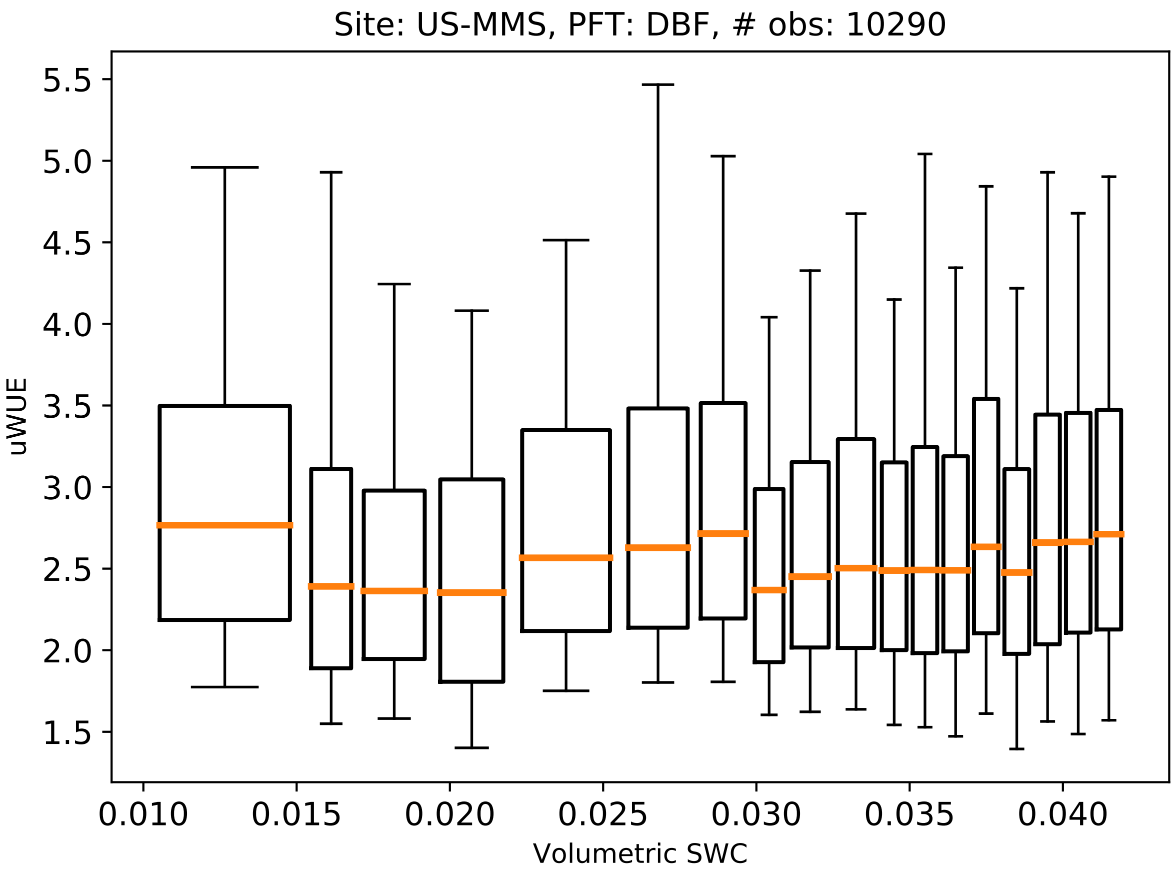

Figure S36. The relationship between uWUE and VPD at the FLUXNET site US-MMS. Each box plot correspondes to 5% of the data. To aid visualization only the 0%-90% range of SWC bins are included.

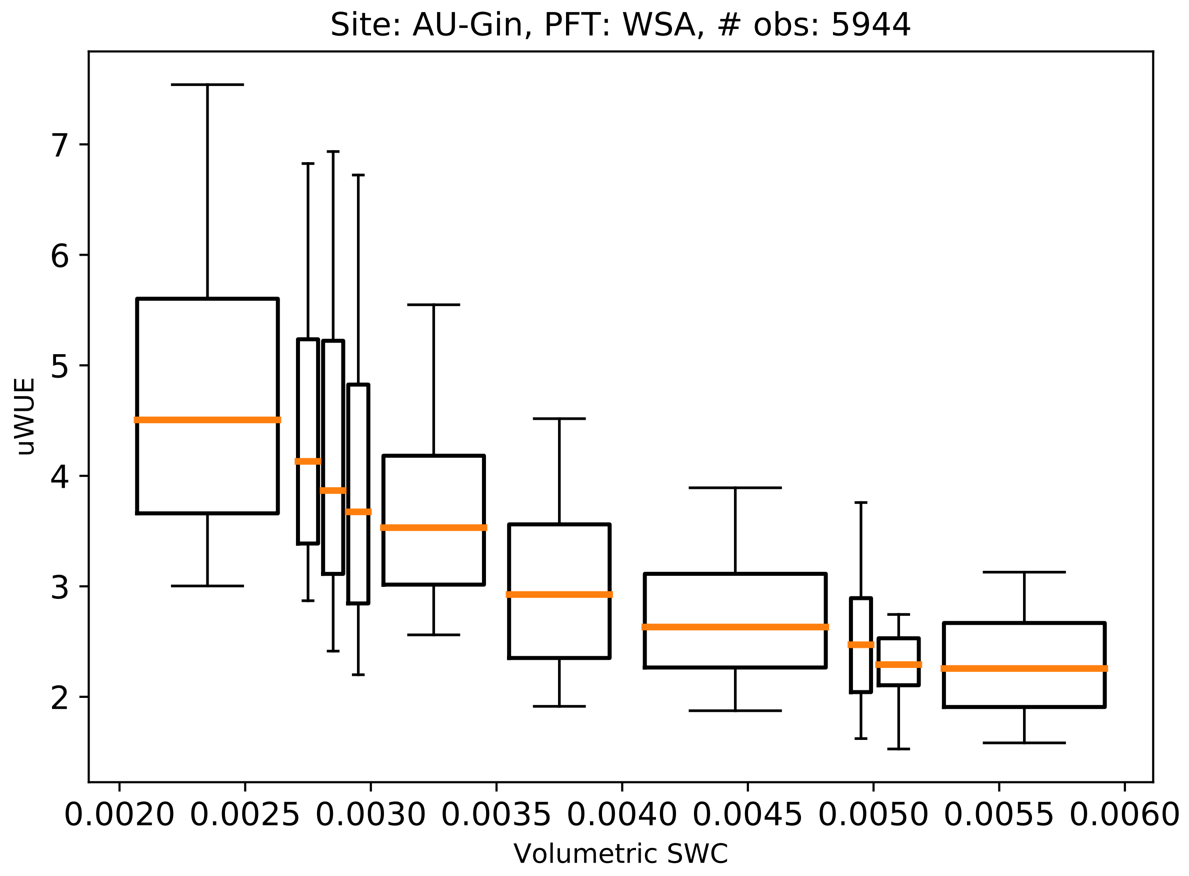

Figure S37. The relationship between uWUE and VPD at the FLUXNET site AU-Gin. Each box plot correspondes to 5% of the data. To aid visualization only the 0%-90% range of SWC bins are included.

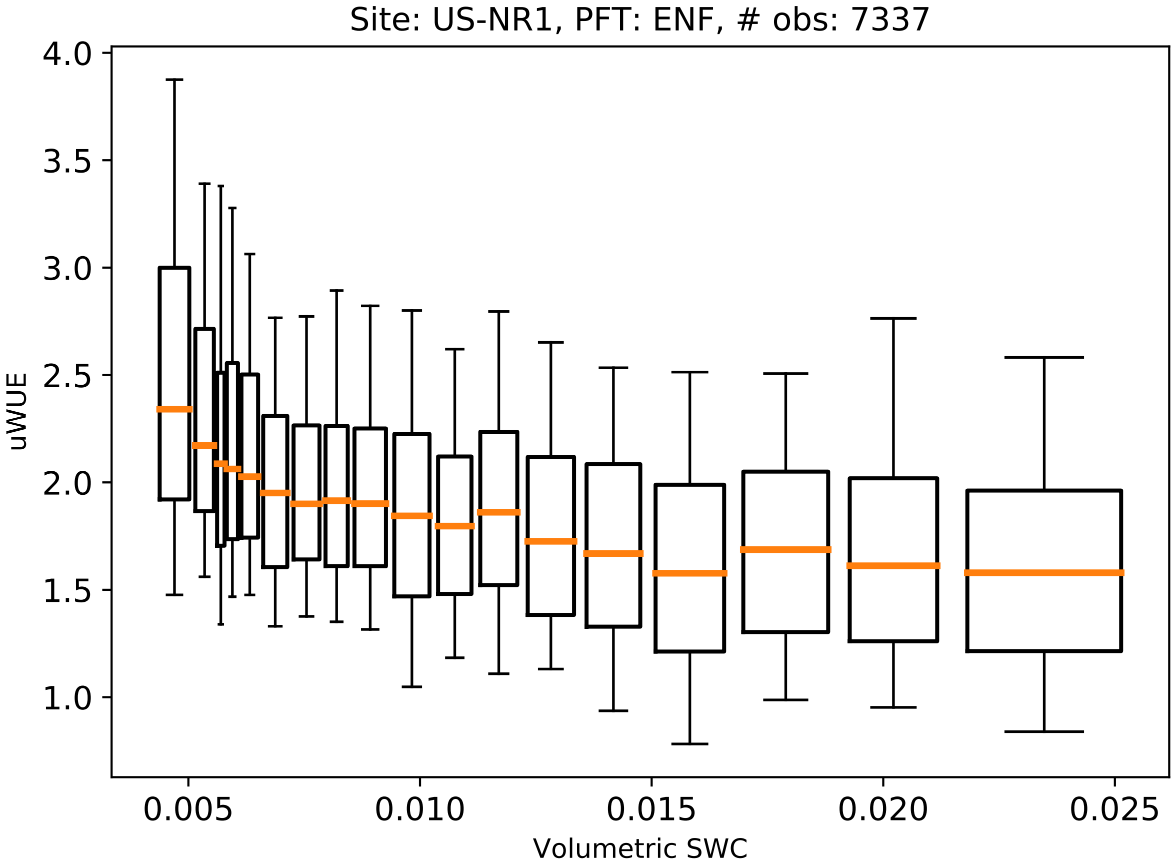

Figure S38. The relationship between uWUE and VPD at the FLUXNET site US-NR1. Each box plot correspondes to 5% of the data. To aid visualization only the 0%-90% range of SWC bins are included.

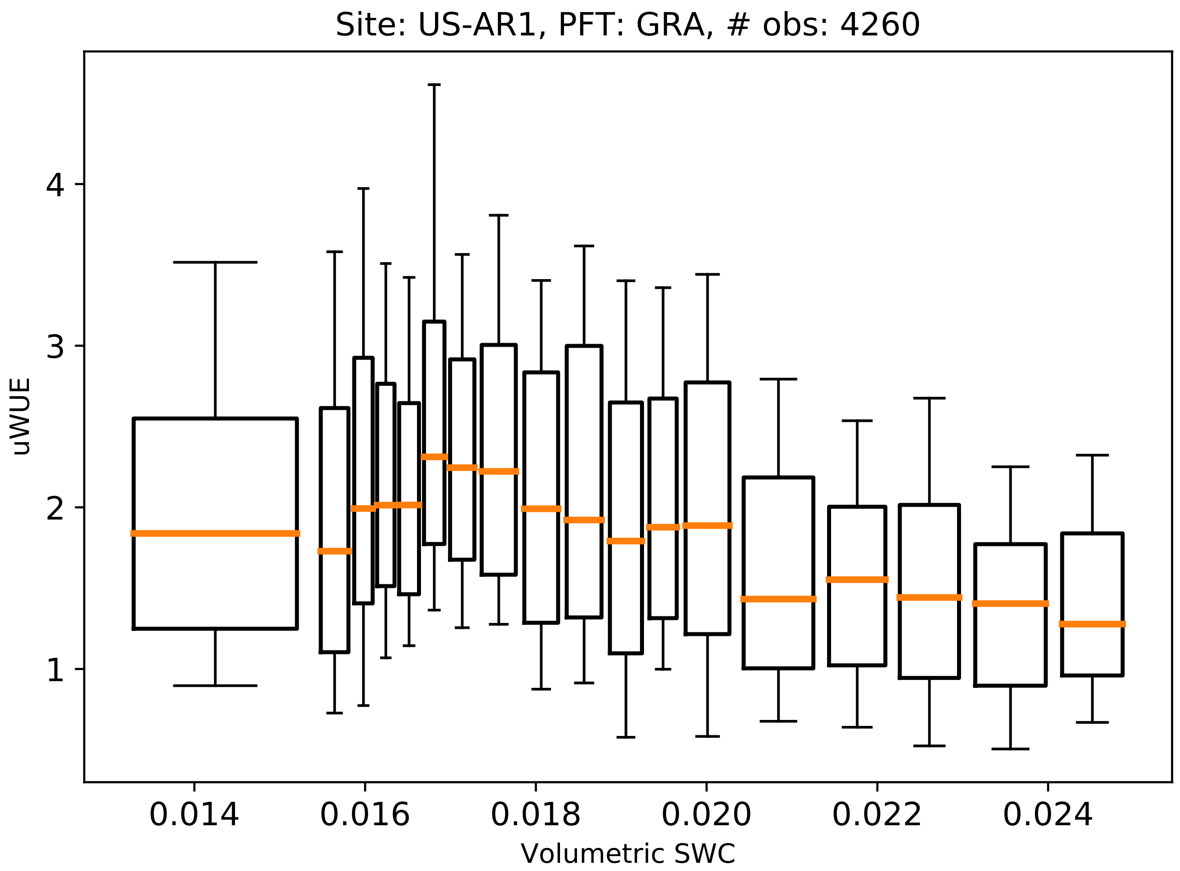

Figure S39. The relationship between uWUE and VPD at the FLUXNET site US-AR1. Each box plot correspondes to 5% of the data. To aid visualization only the 0%-90% range of SWC bins are included.

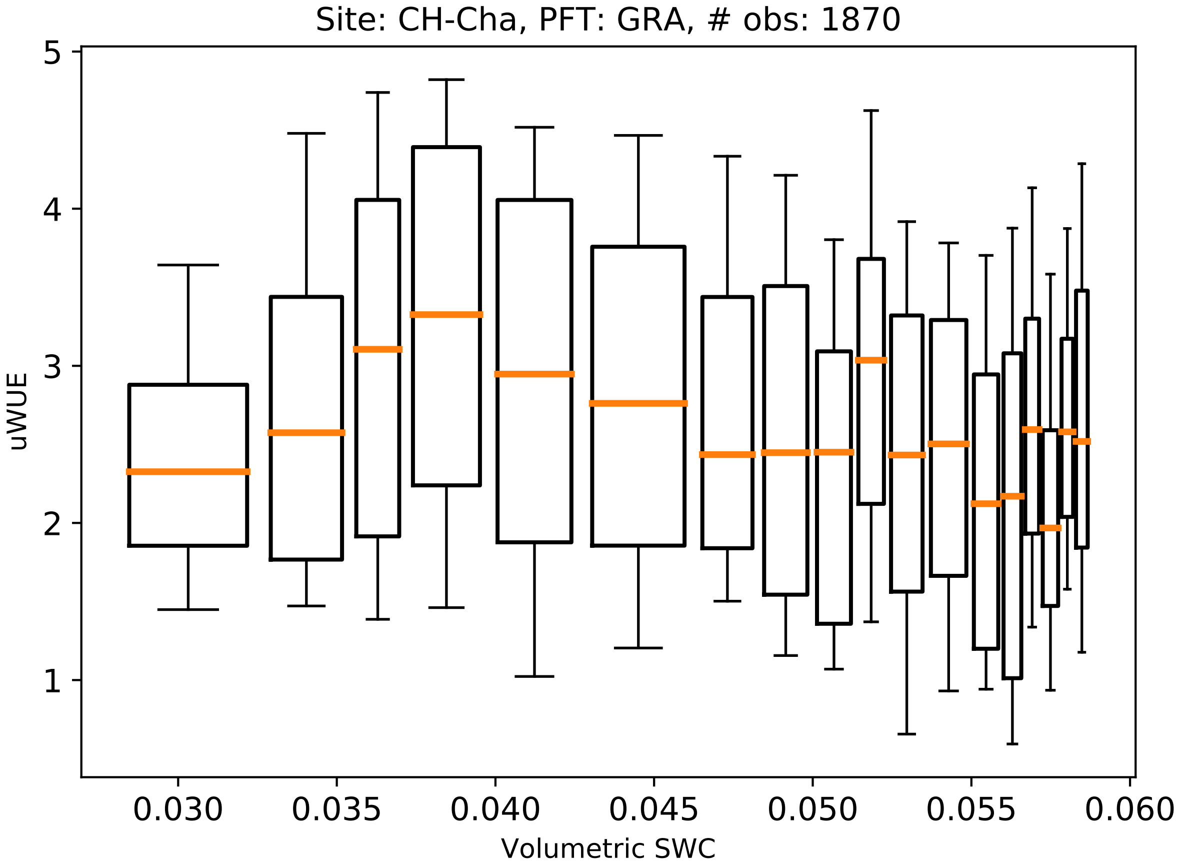

Figure S40. The relationship between uWUE and VPD at the FLUXNET site CH-Cha. Each box plot correspondes to 5% of the data. To aid visualization only the 0%-90% range of SWC bins are included.

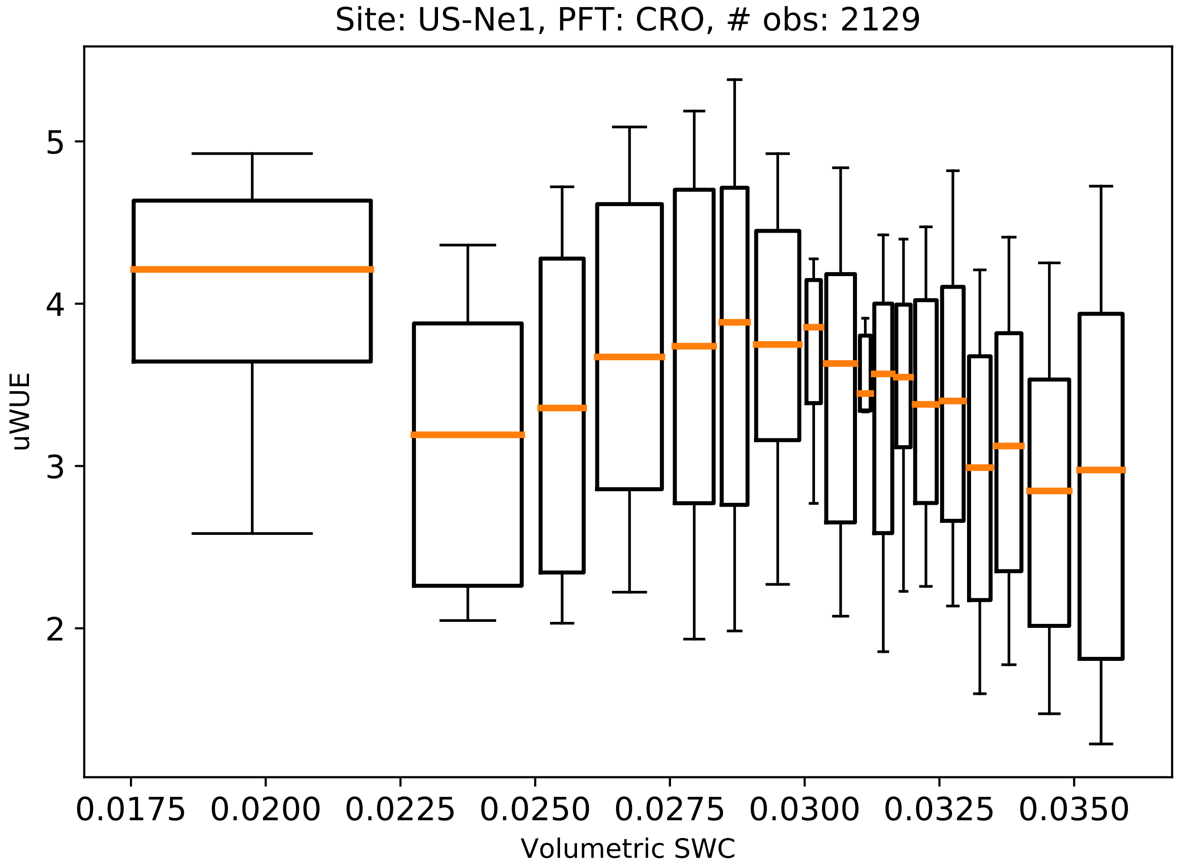

Figure S41. The relationship between uWUE and VPD at the FLUXNET site US-Ne1. Each box plot correspondes to 5% of the data. To aid visualization only the 0%-90% range of SWC bins are included.

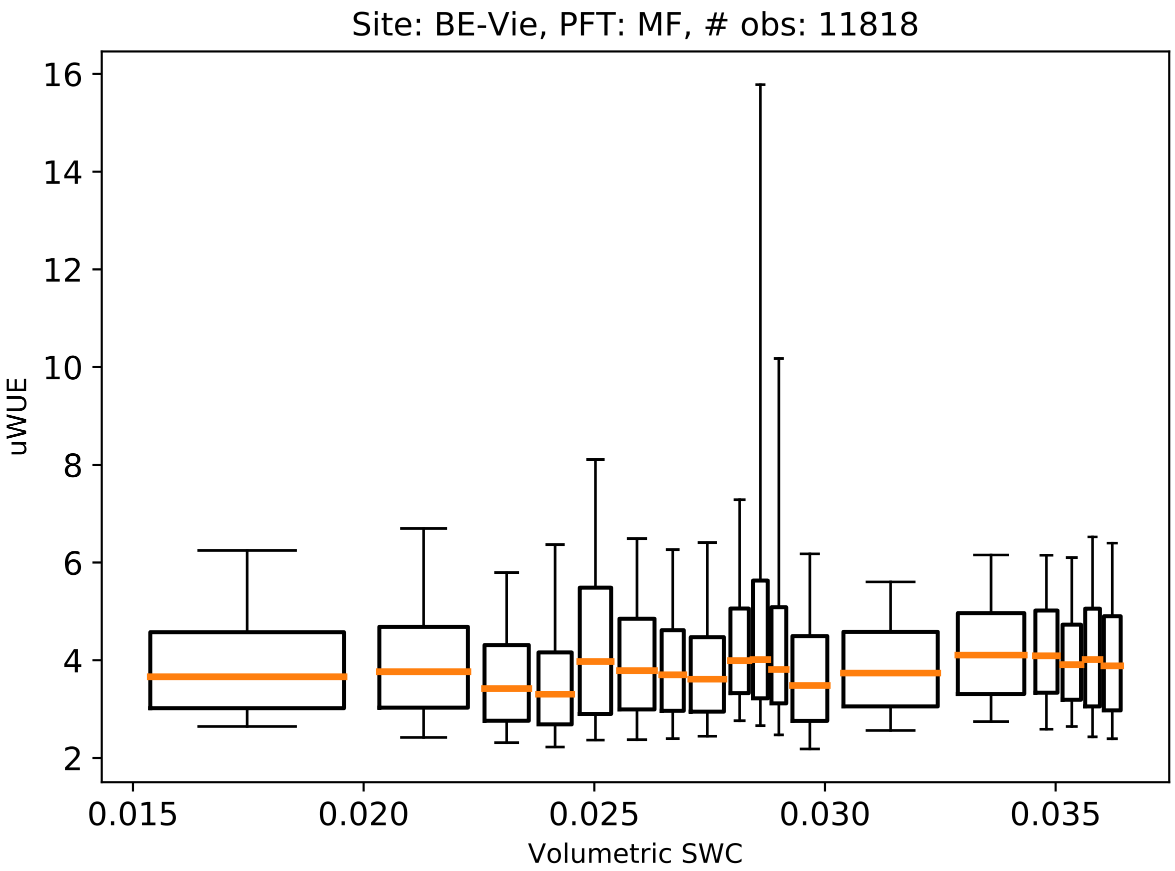

Figure S42. The relationship between uWUE and VPD at the FLUXNET site BE-Vie. Each box plot correspondes to 5% of the data. To aid visualization only the 0%-90% range of SWC bins are included.

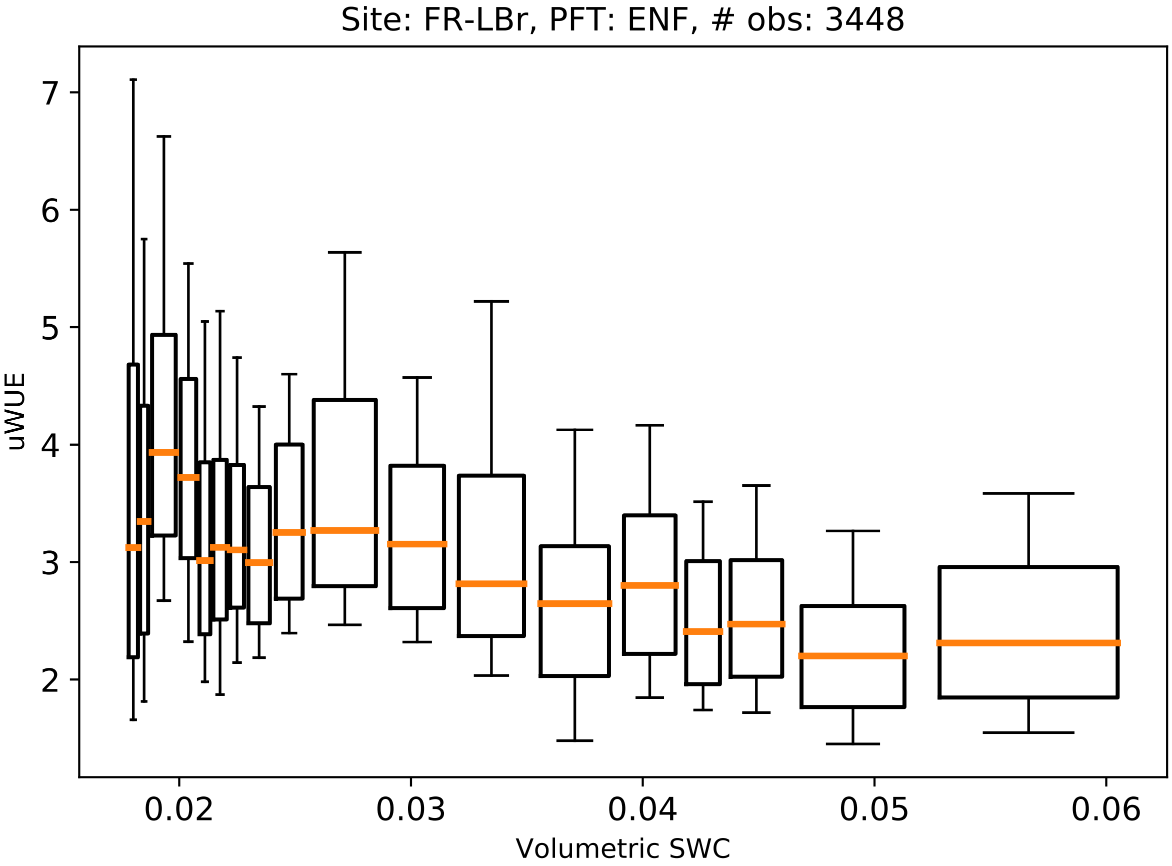

Figure S43. The relationship between uWUE and VPD at the FLUXNET site FR-LBr. Each box plot correspondes to 5% of the data. To aid visualization only the 0%-90% range of SWC bins are included.

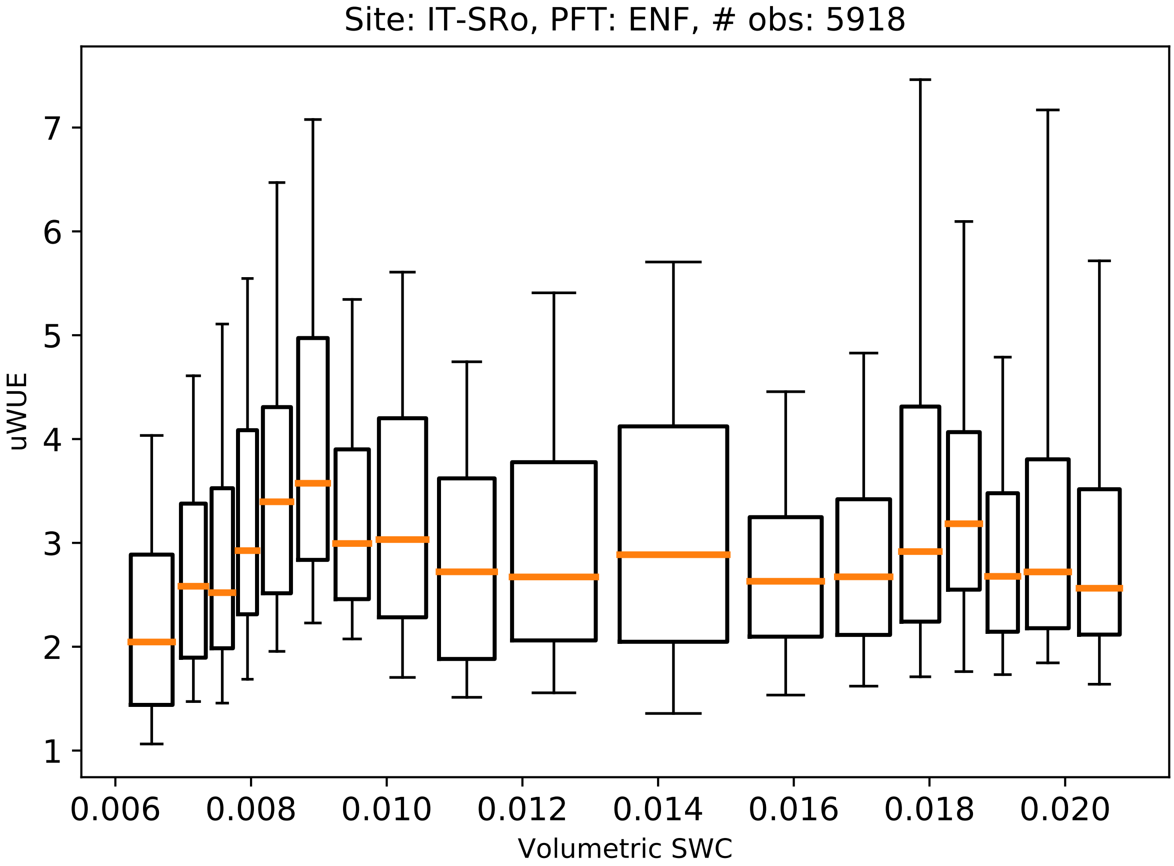

Figure S44. The relationship between uWUE and VPD at the FLUXNET site IT-SRo. Each box plot correspondes to 5% of the data. To aid visualization only the 0%-90% range of SWC bins are included.

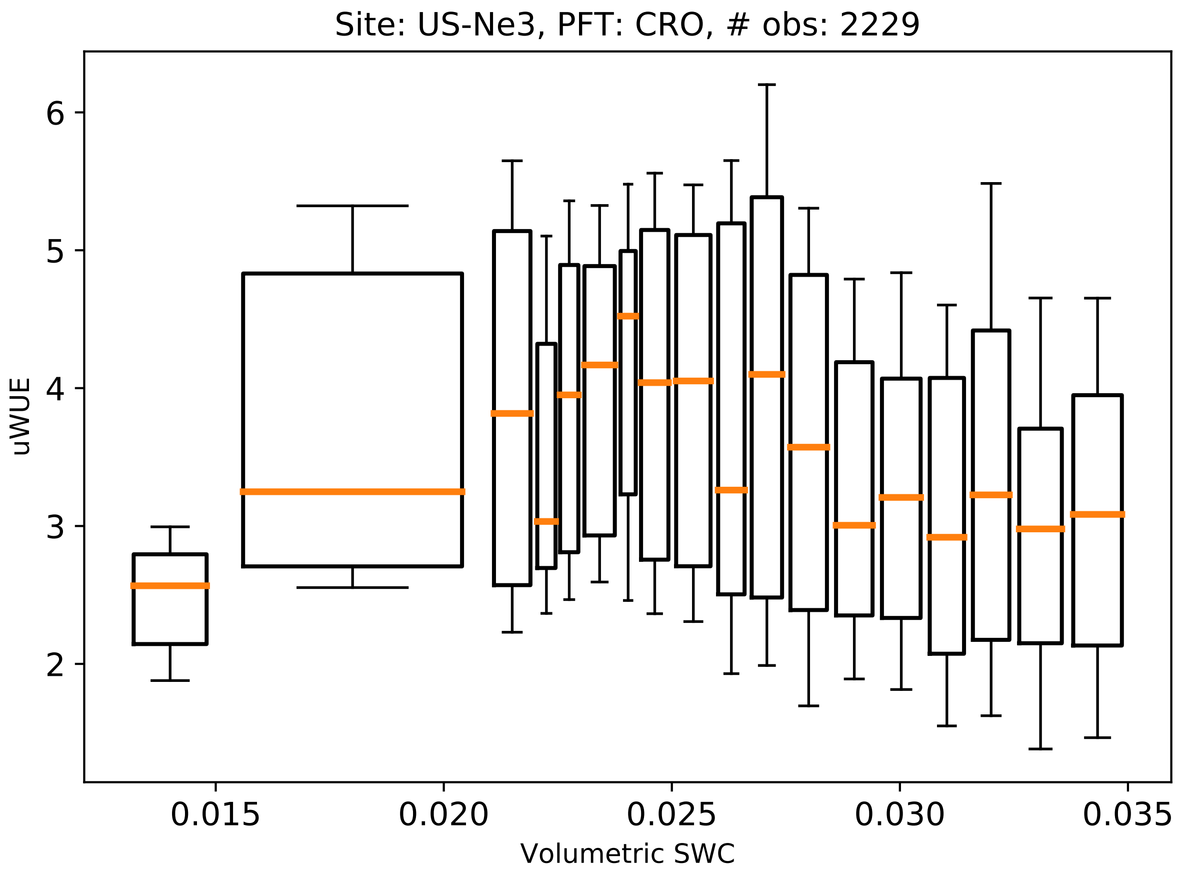

Figure S45. The relationship between uWUE and VPD at the FLUXNET site US-Ne3. Each box plot correspondes to 5% of the data. To aid visualization only the 0%-90% range of SWC bins are included.

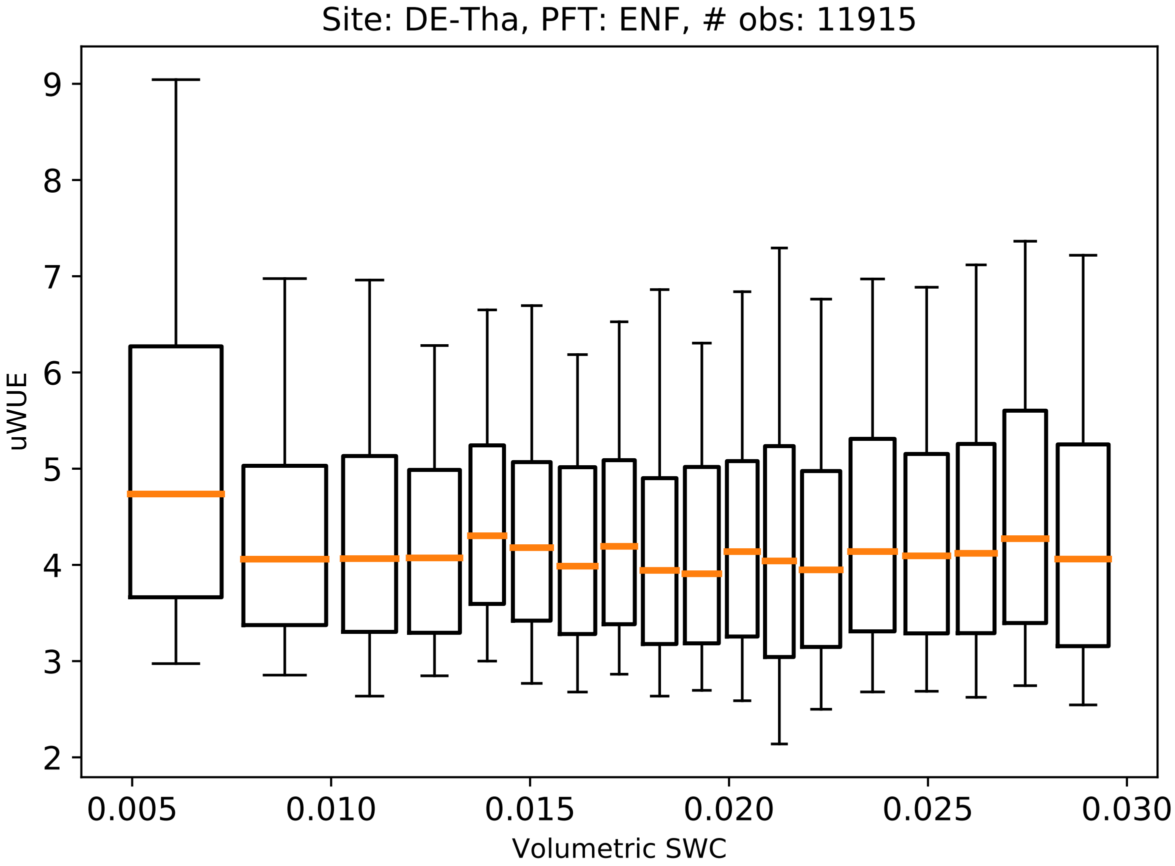

Figure S46. The relationship between uWUE and VPD at the FLUXNET site DE-Tha. Each box plot correspondes to 5% of the data. To aid visualization only the 0%-90% range of SWC bins are included.

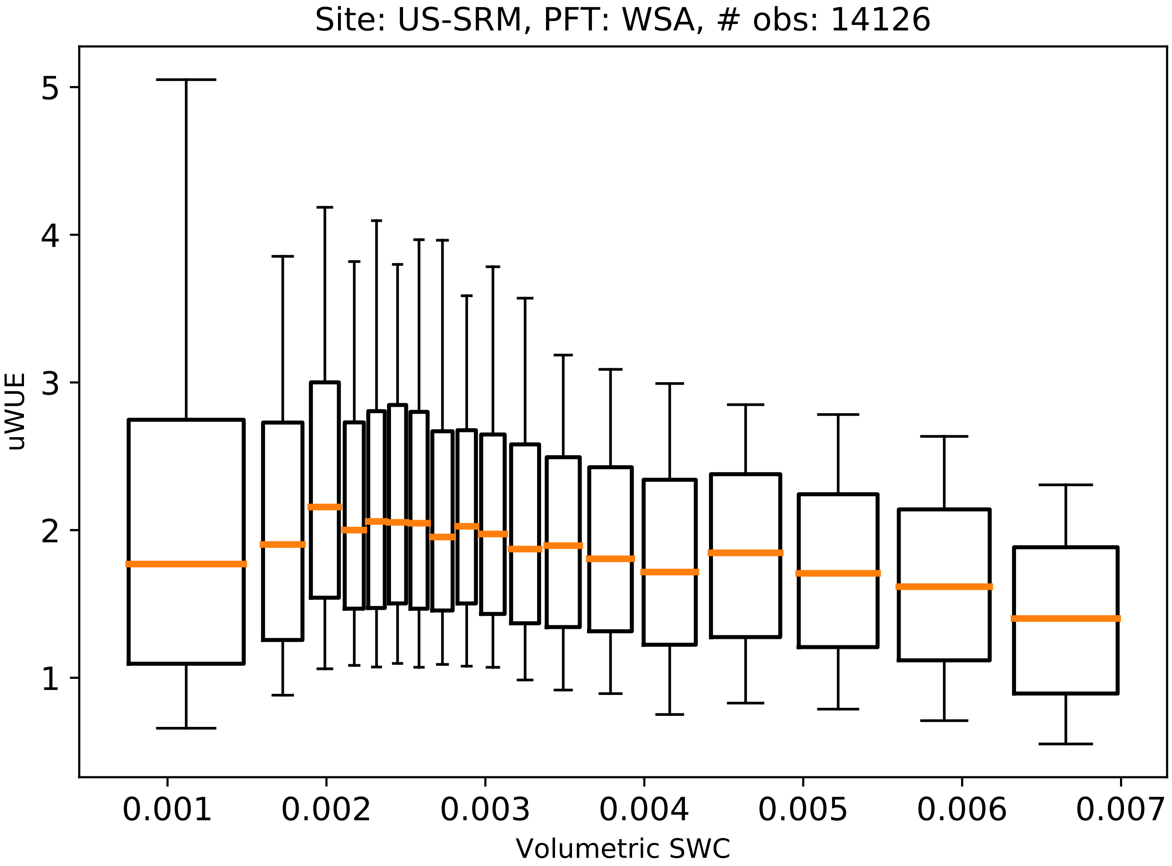

Figure S47. The relationship between uWUE and VPD at the FLUXNET site US-SRM. Each box plot correspondes to 5% of the data. To aid visualization only the 0%-90% range of SWC bins are included.

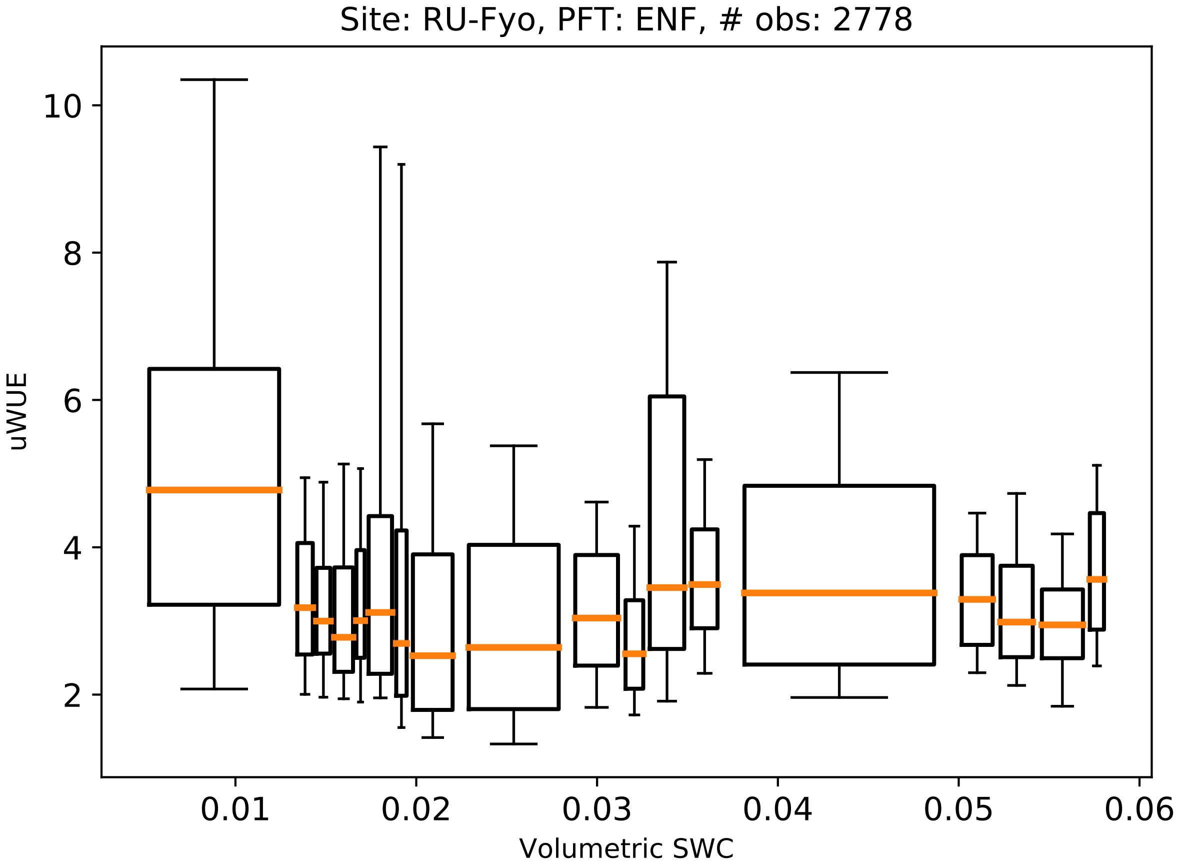

Figure S48. The relationship between uWUE and VPD at the FLUXNET site RU-Fyo. Each box plot correspondes to 5% of the data. To aid visualization only the 0%-90% range of SWC bins are included.

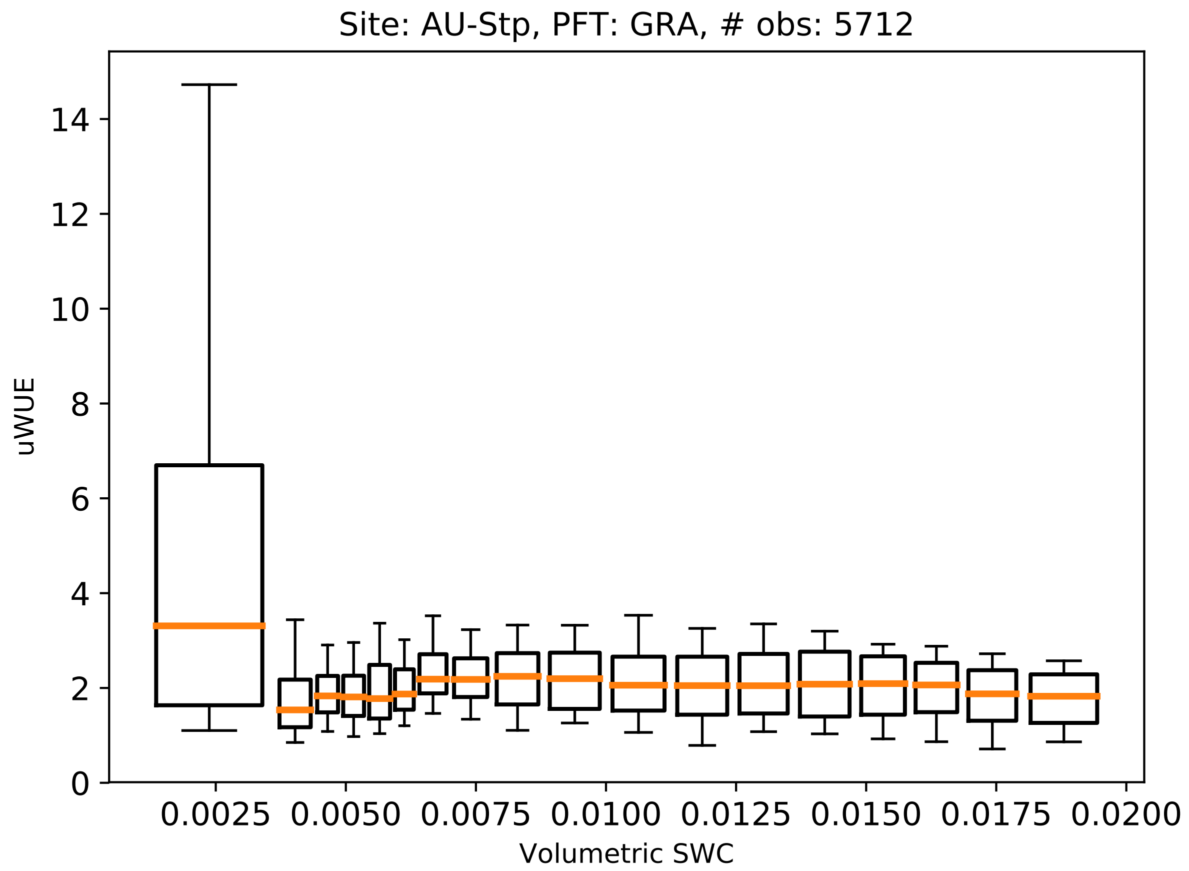

Figure S49. The relationship between uWUE and VPD at the FLUXNET site AU-Stp. Each box plot correspondes to 5% of the data. To aid visualization only the 0%-90% range of SWC bins are included.

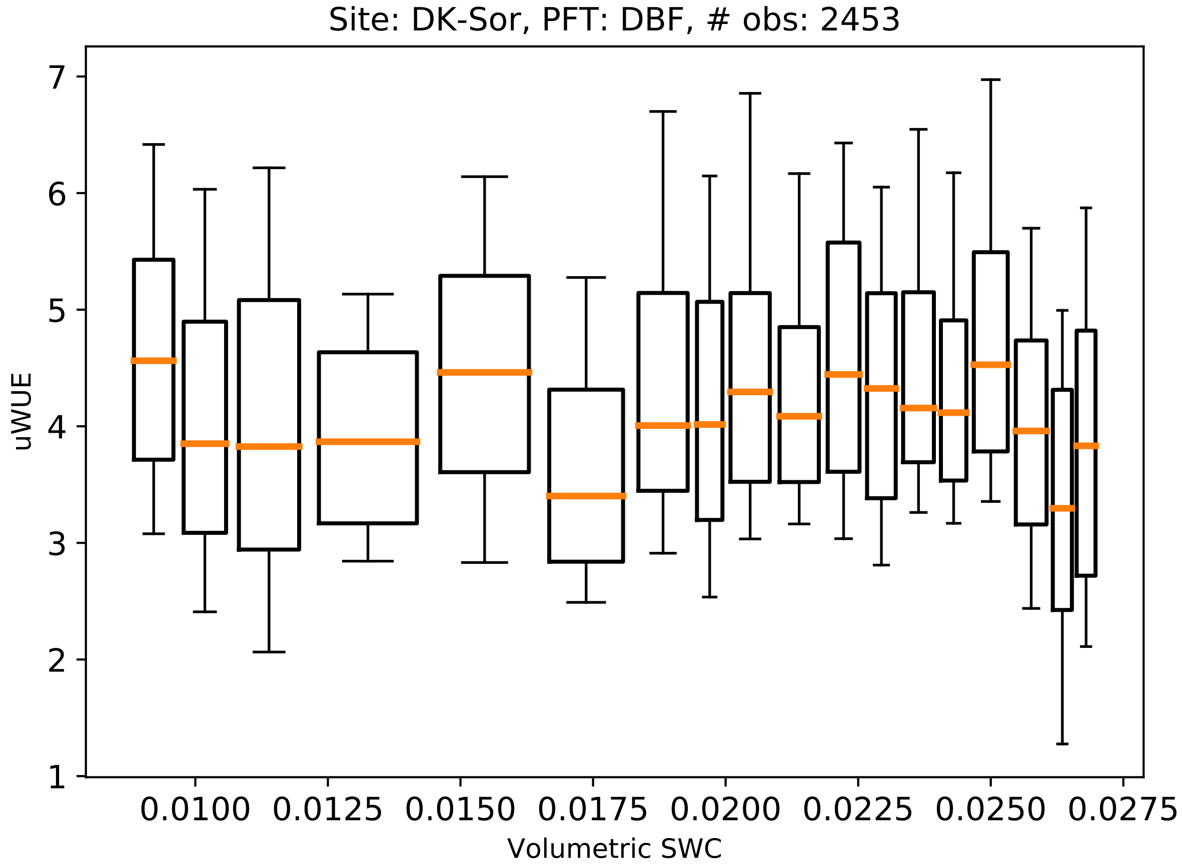

Figure S50. The relationship between uWUE and VPD at the FLUXNET site DK-Sor. Each box plot correspondes to 5% of the data. To aid visualization only the 0%-90% range of SWC bins are included.

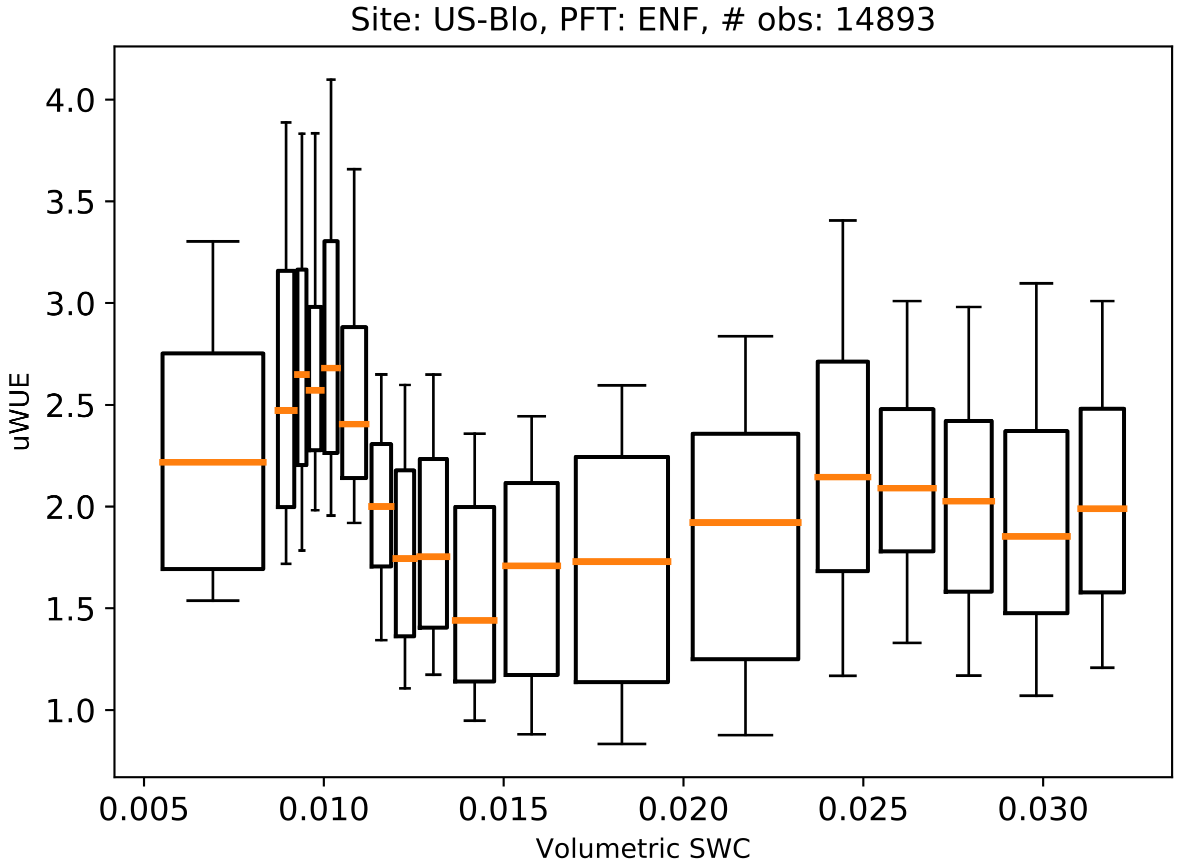

Figure S51. The relationship between uWUE and VPD at the FLUXNET site US-Blo. Each box plot correspondes to 5% of the data. To aid visualization only the 0%-90% range of SWC bins are included.

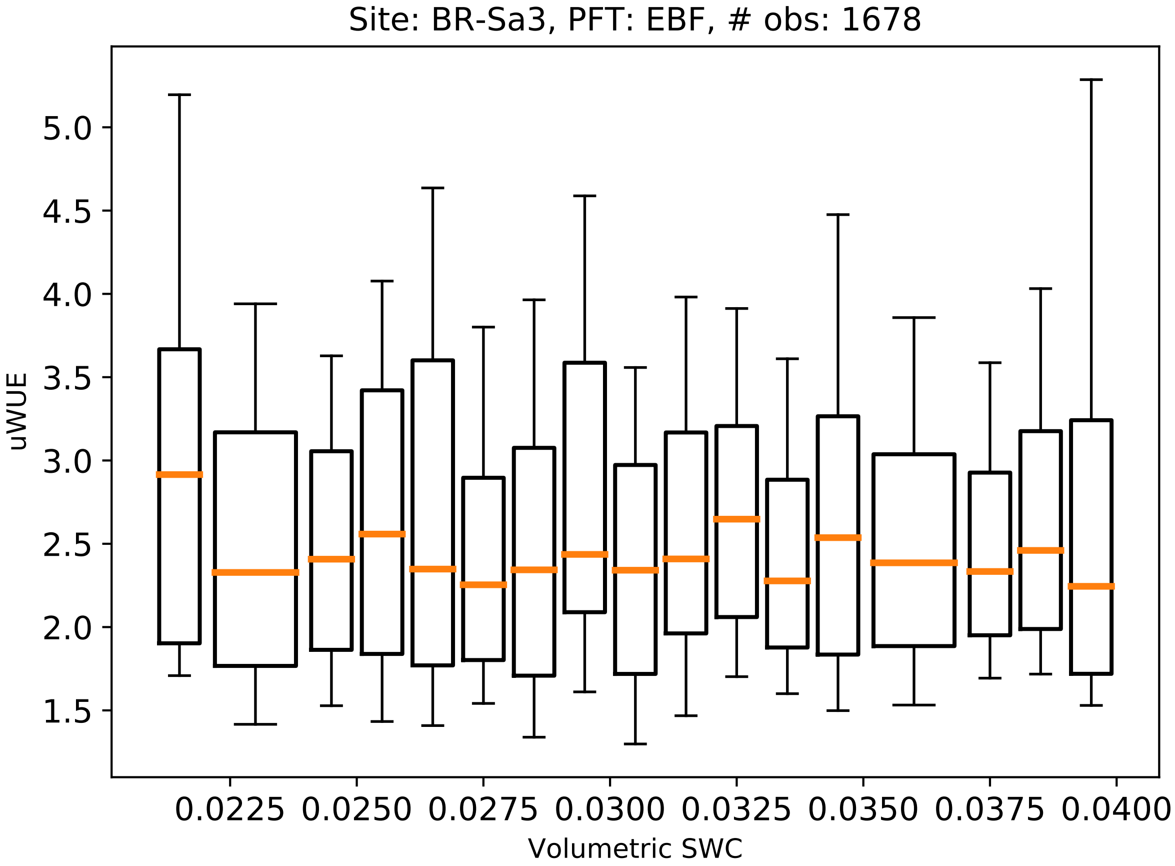

Figure S52. The relationship between uWUE and VPD at the FLUXNET site BR-Sa3. Each box plot correspondes to 5% of the data. To aid visualization only the 0%-90% range of SWC bins are included.

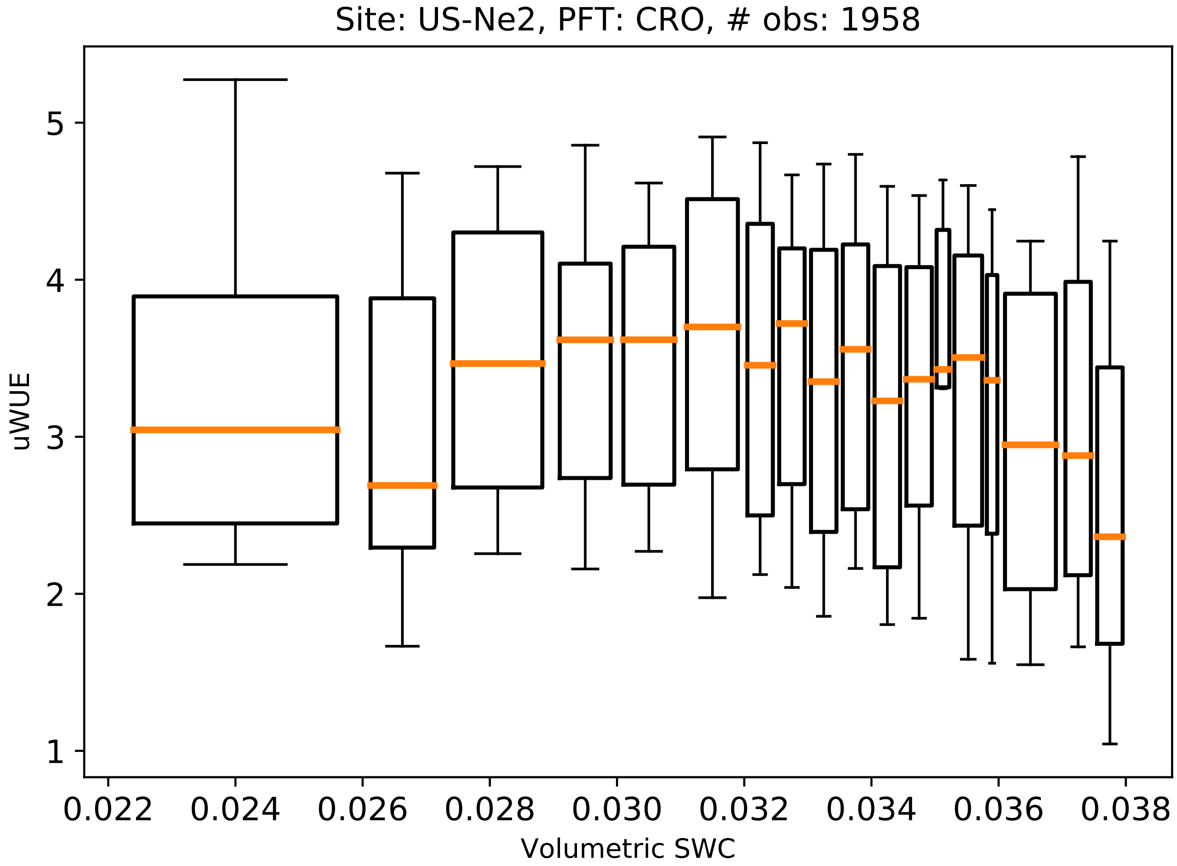

Figure S53. The relationship between uWUE and VPD at the FLUXNET site US-Ne2. Each box plot correspondes to 5% of the data. To aid visualization only the 0%-90% range of SWC bins are included.

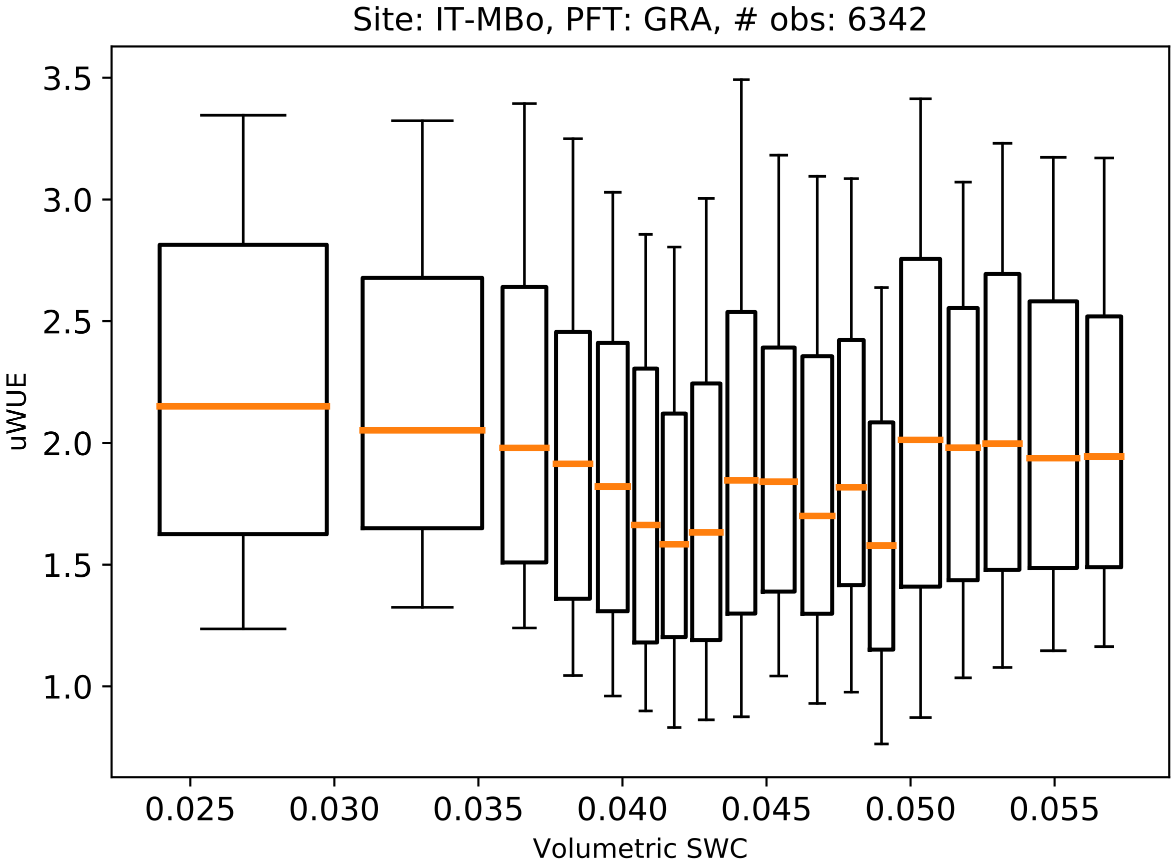

Figure S54. The relationship between uWUE and VPD at the FLUXNET site IT-MBo. Each box plot correspondes to 5% of the data. To aid visualization only the 0%-90% range of SWC bins are included.

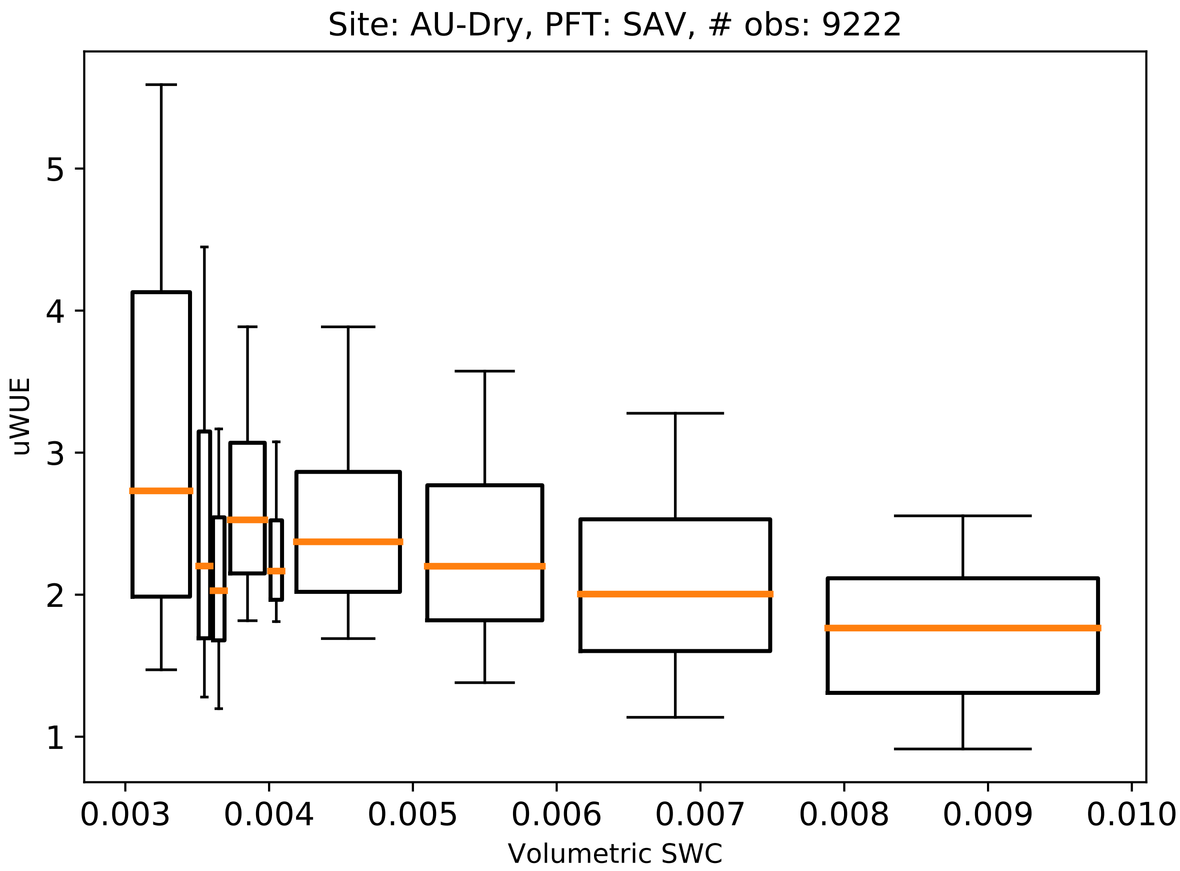

Figure S55. The relationship between uWUE and VPD at the FLUXNET site AU-Dry. Each box plot correspondes to 5% of the data. To aid visualization only the 0%-90% range of SWC bins are included.

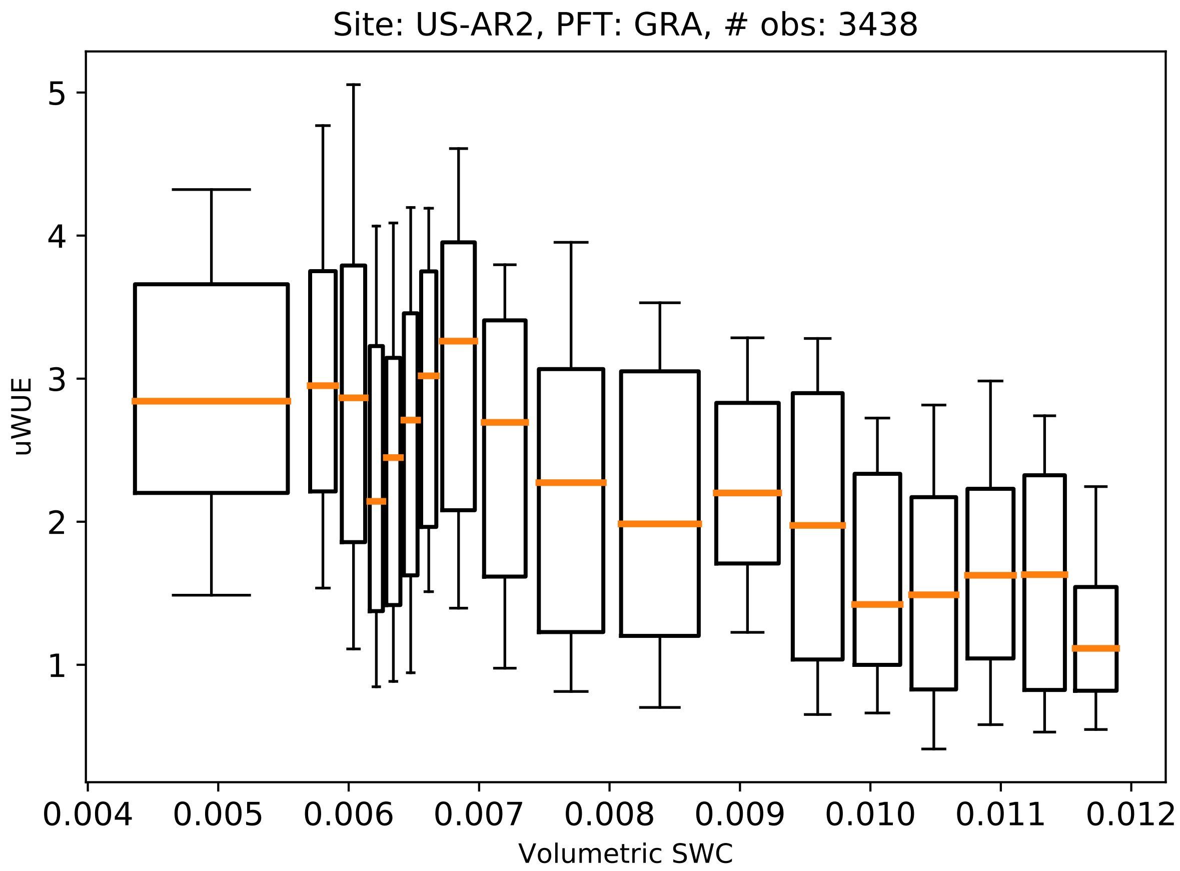

Figure S56. The relationship between uWUE and VPD at the FLUXNET site US-AR2. Each box plot correspondes to 5% of the data. To aid visualization only the 0%-90% range of SWC bins are included.

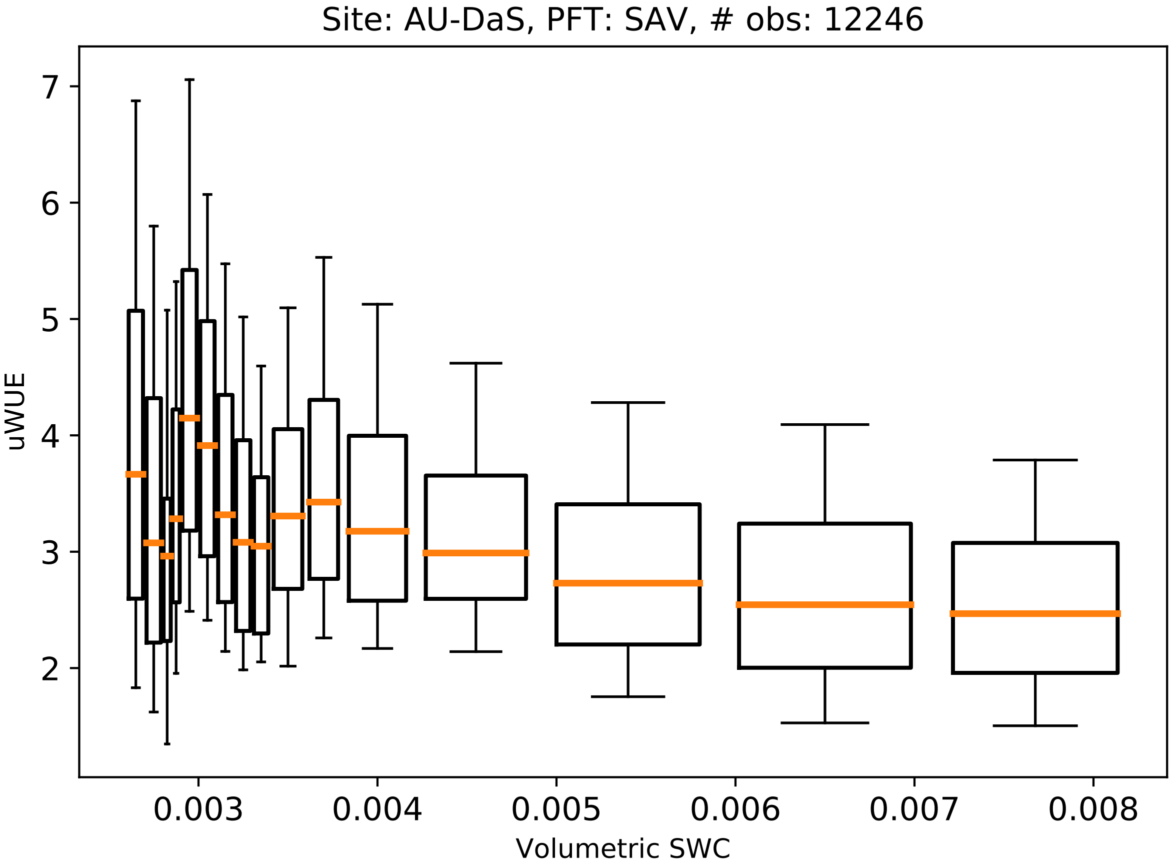

Figure S57. The relationship between uWUE and VPD at the FLUXNET site AU-DaS. Each box plot correspondes to 5% of the data. To aid visualization only the 0%-90% range of SWC bins are included.

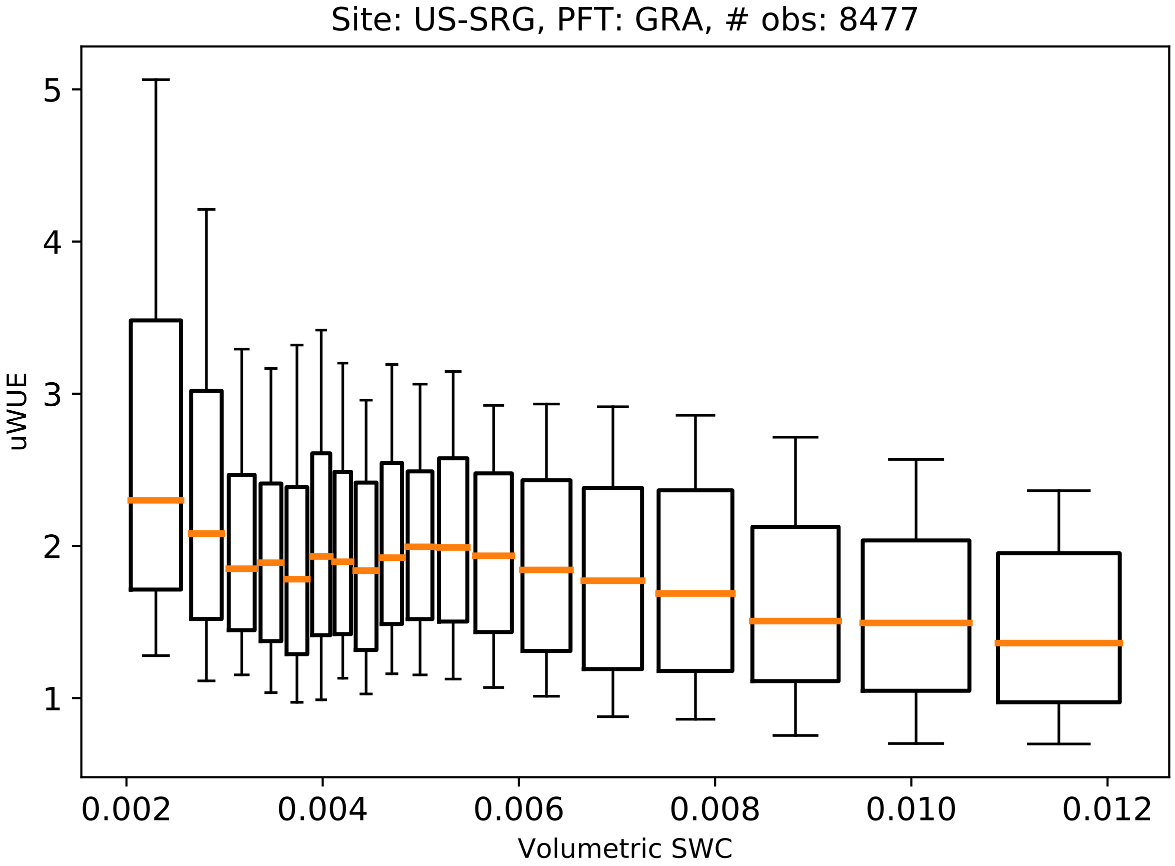

Figure S58. The relationship between uWUE and VPD at the FLUXNET site US-SRG. Each box plot correspondes to 5% of the data. To aid visualization only the 0%-90% range of SWC bins are included.

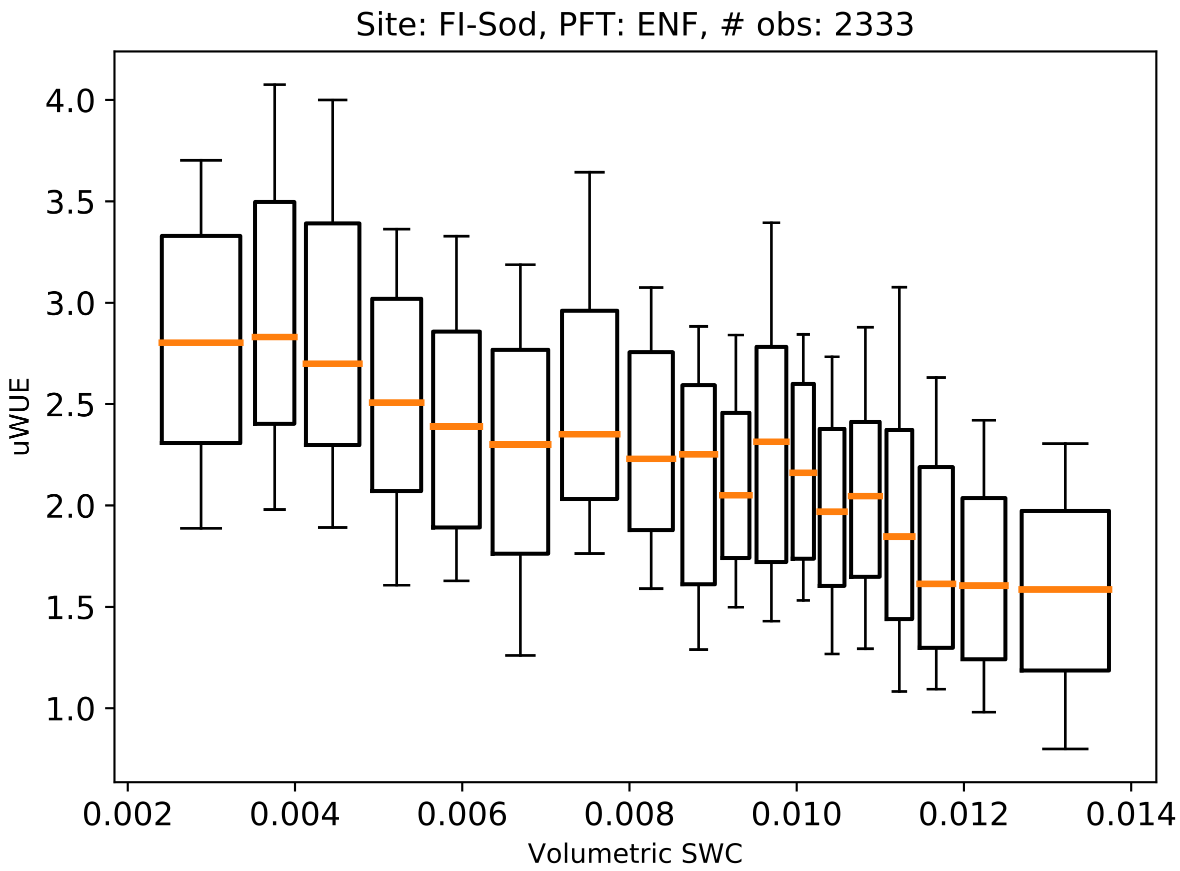

Figure S59. The relationship between uWUE and VPD at the FLUXNET site FI-Sod. Each box plot correspondes to 5% of the data. To aid visualization only the 0%-90% range of SWC bins are included.

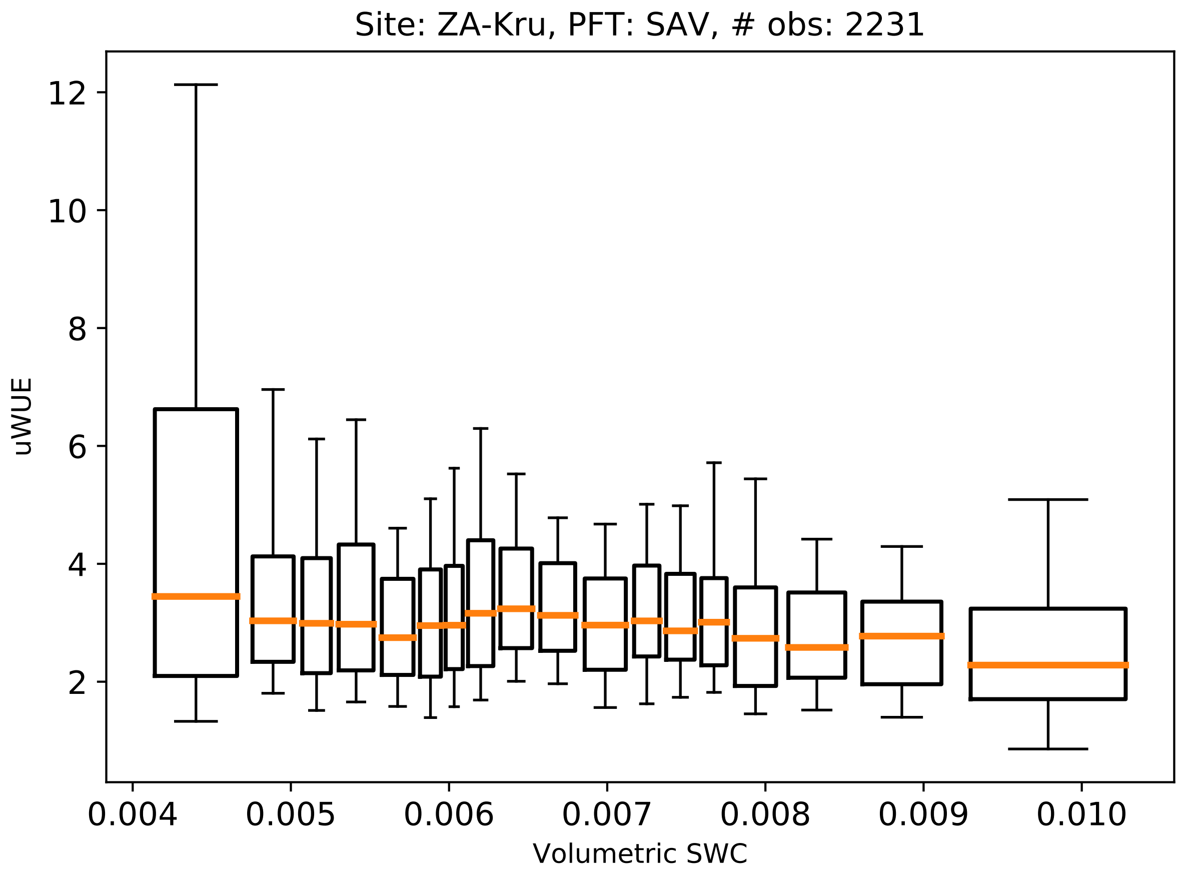

Figure S60. The relationship between uWUE and VPD at the FLUXNET site ZA-Kru. Each box plot correspondes to 5% of the data. To aid visualization only the 0%-90% range of SWC bins are included.

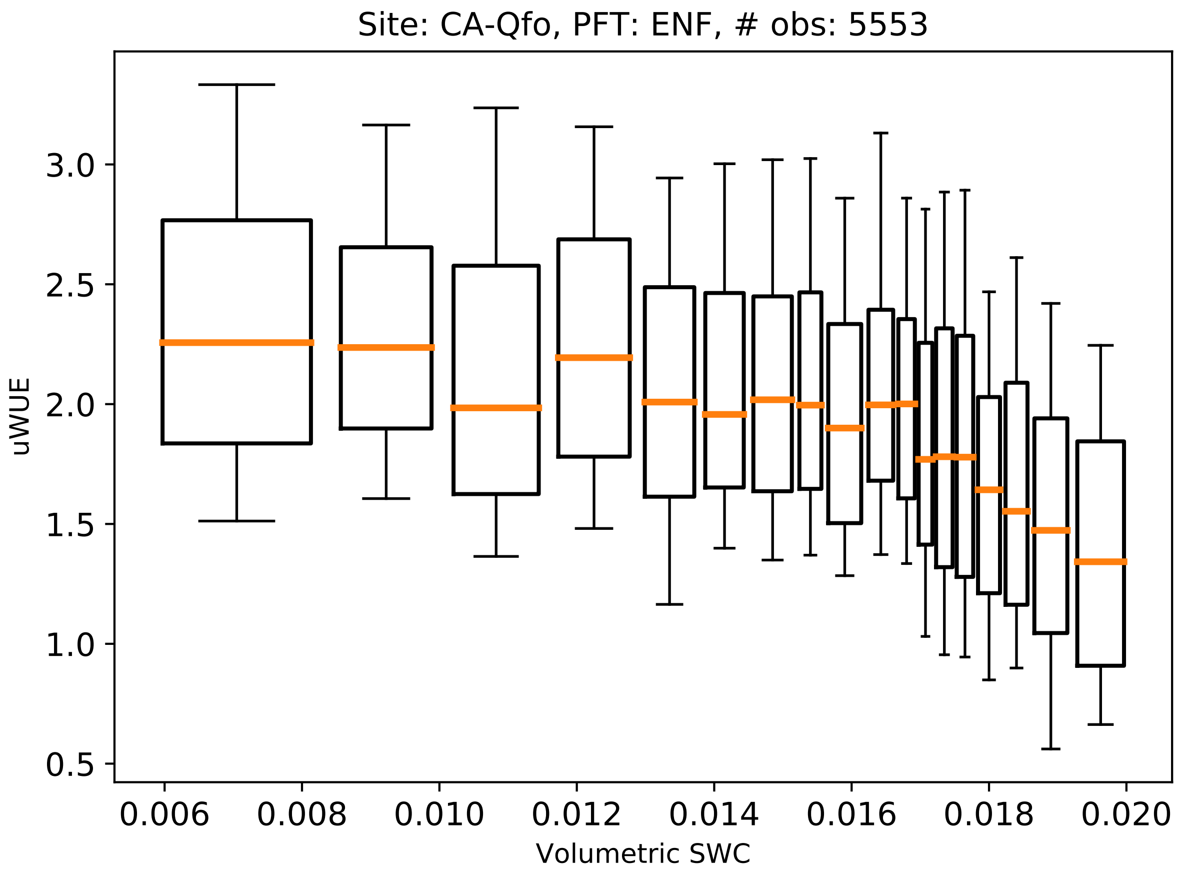

Figure S61. The relationship between uWUE and VPD at the FLUXNET site CA-Qfo. Each box plot correspondes to 5% of the data. To aid visualization only the 0%-90% range of SWC bins are included.

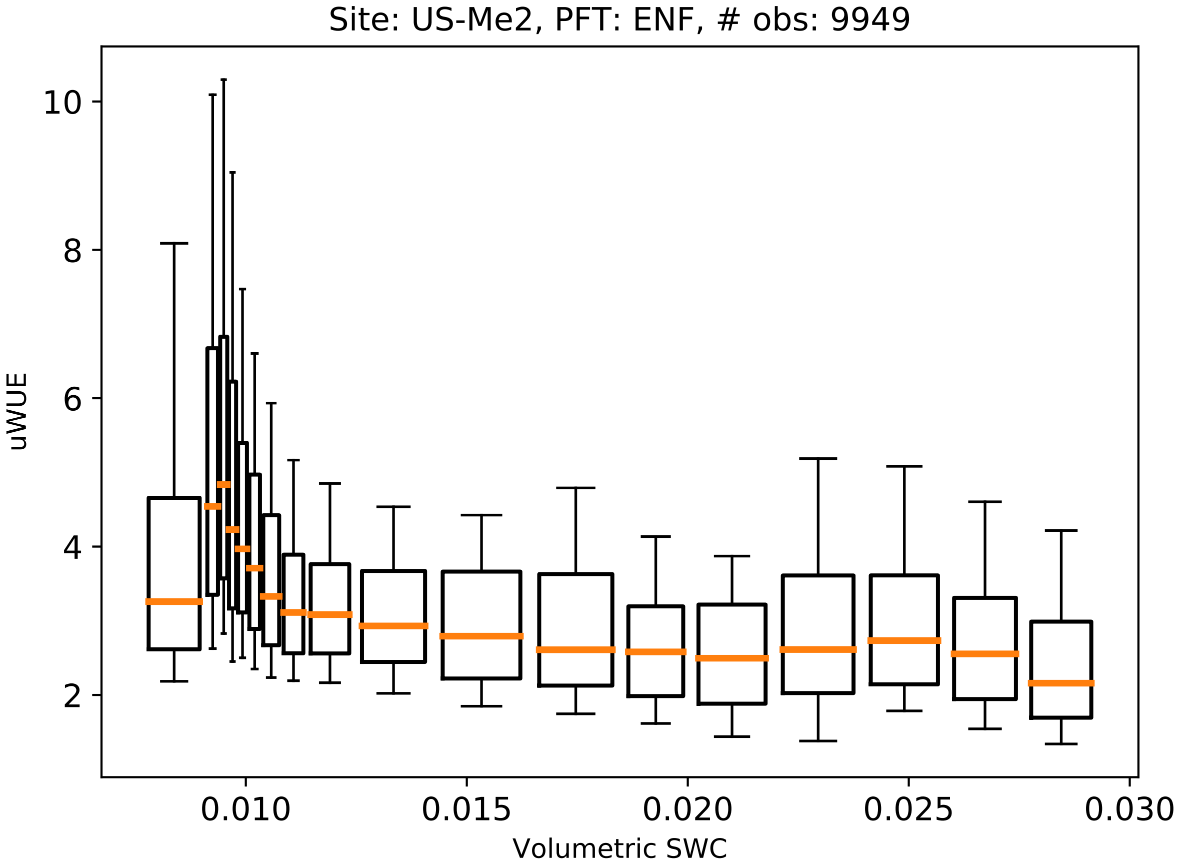

Figure S62. The relationship between uWUE and VPD at the FLUXNET site US-Me2. Each box plot correspondes to 5% of the data. To aid visualization only the 0%-90% range of SWC bins are included.

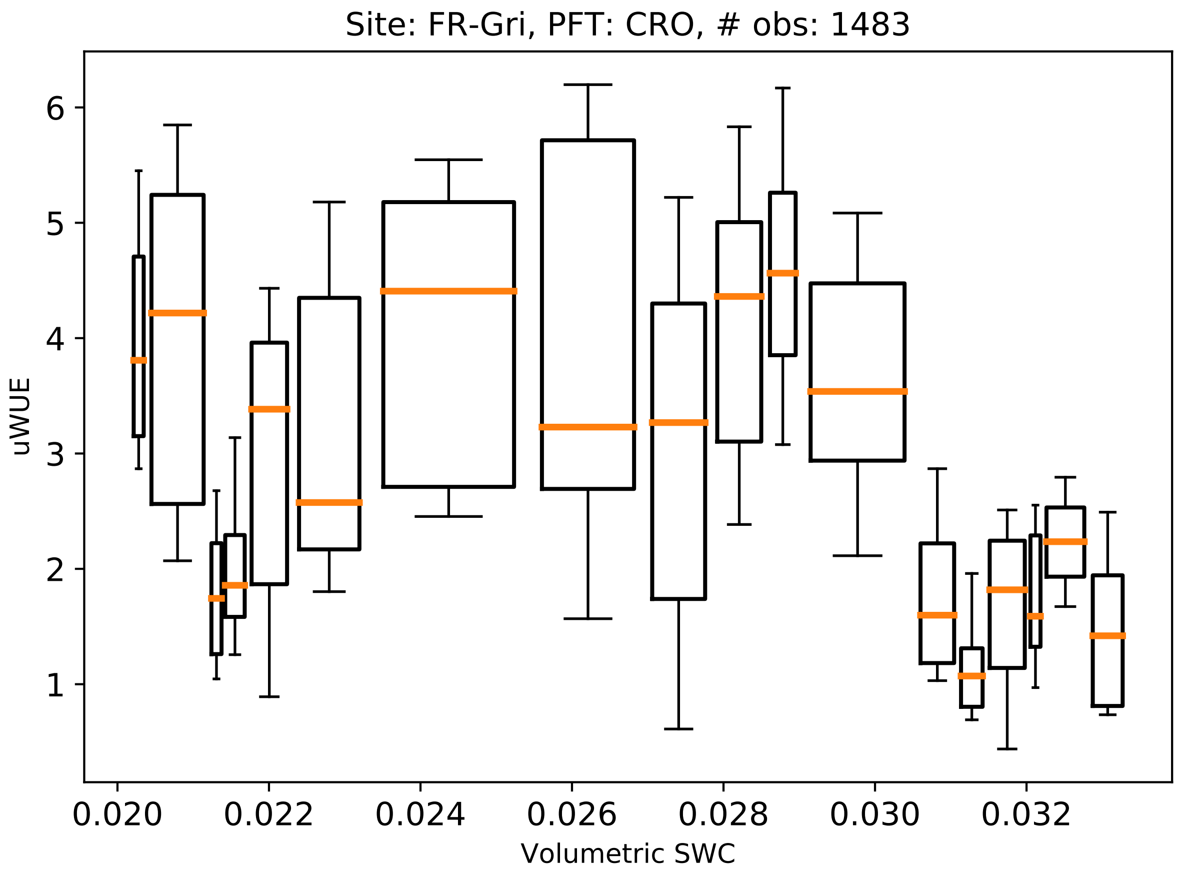

Figure S63. The relationship between uWUE and VPD at the FLUXNET site FR-Gri. Each box plot correspondes to 5% of the data. To aid visualization only the 0%-90% range of SWC bins are included.

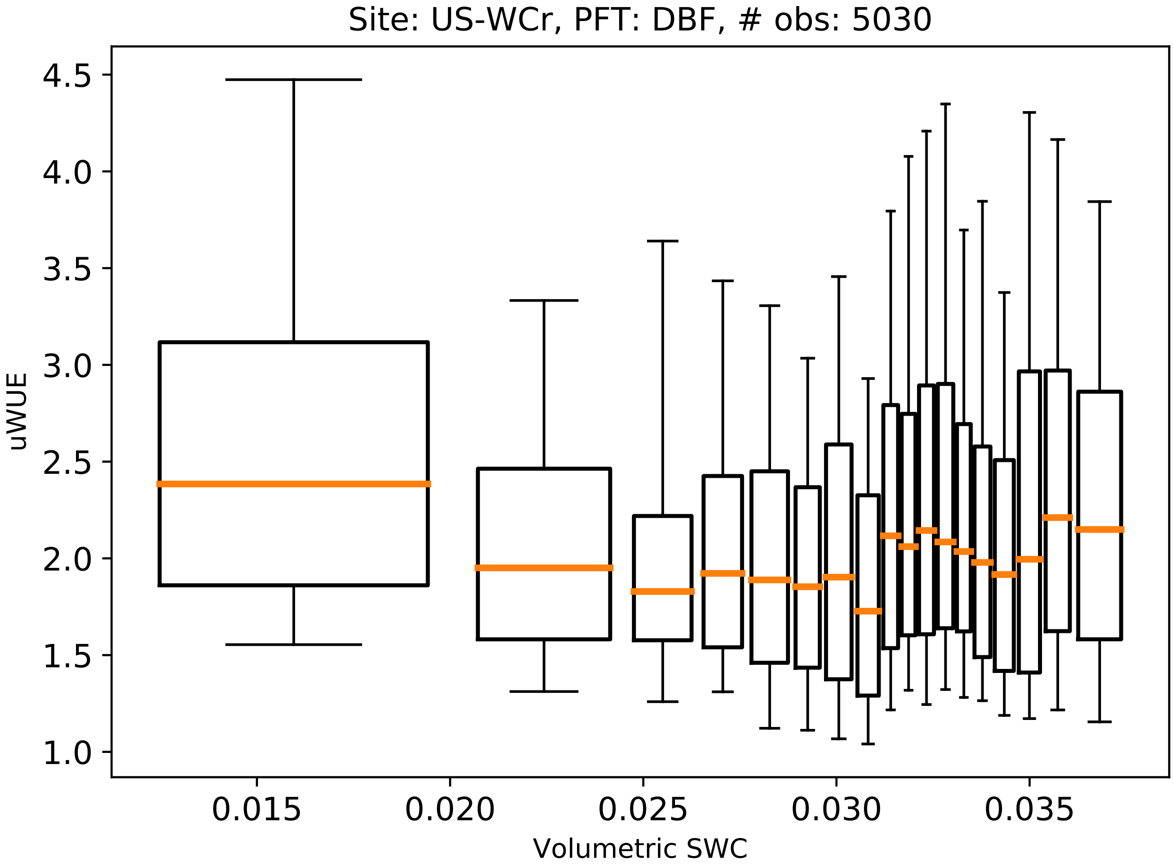

Figure S64. The relationship between uWUE and VPD at the FLUXNET site US-WCr. Each box plot correspondes to 5% of the data. To aid visualization only the 0%-90% range of SWC bins are included.

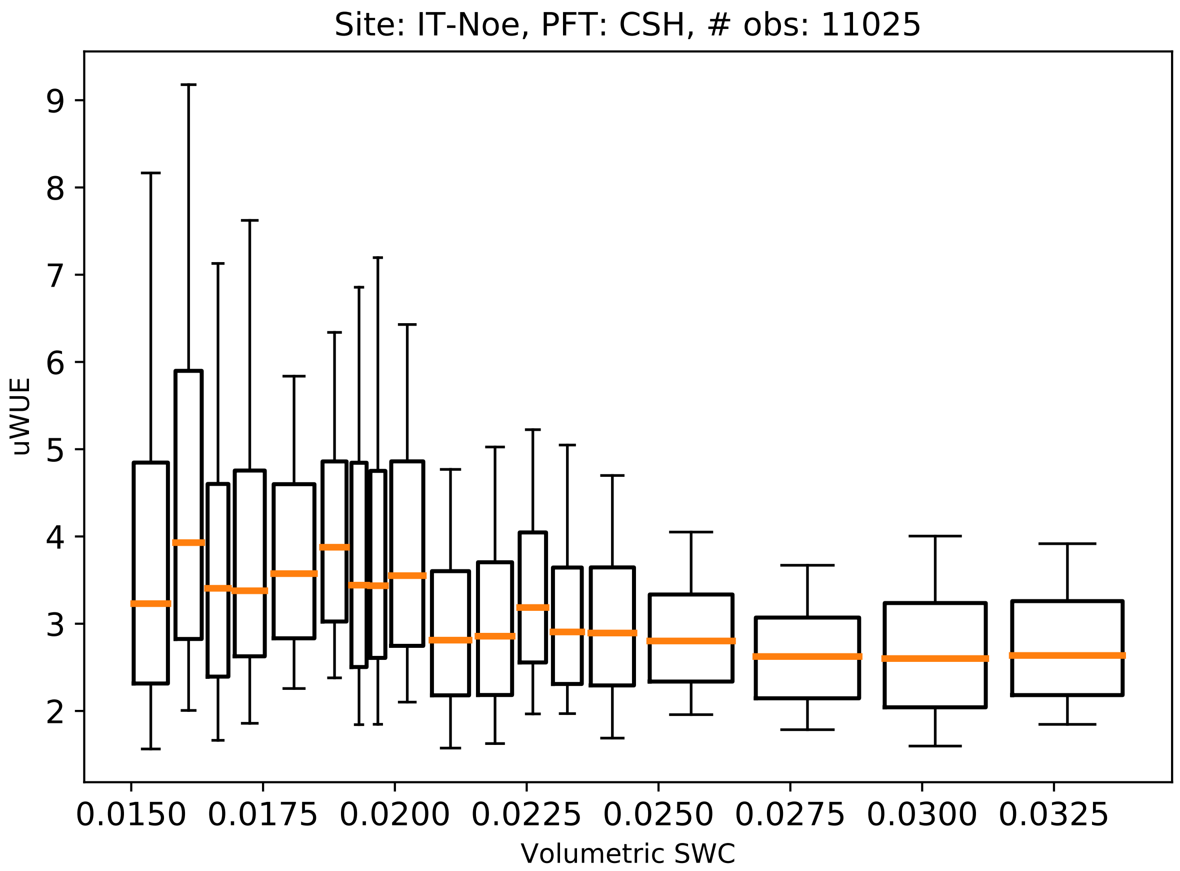

Figure S65. The relationship between uWUE and VPD at the FLUXNET site IT-Noe. Each box plot correspondes to 5% of the data. To aid visualization only the 0%-90% range of SWC bins are included.

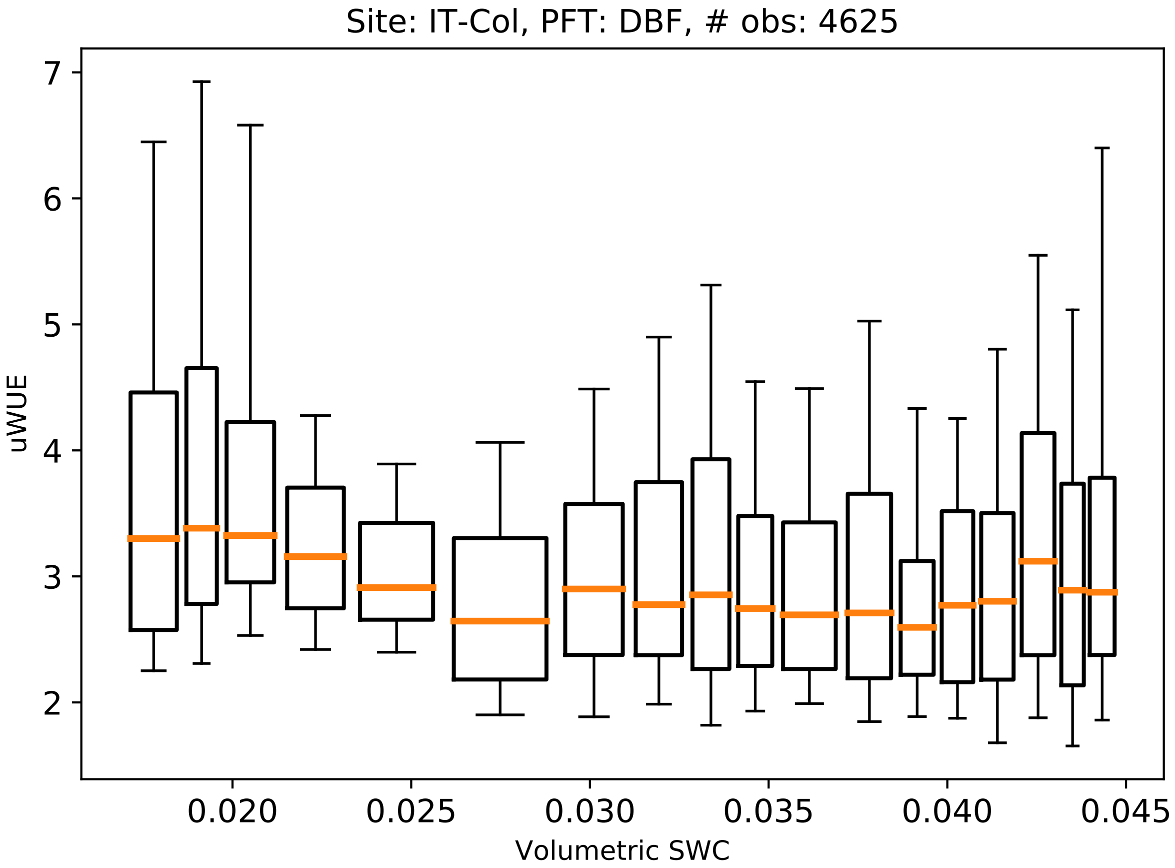

Figure S66. The relationship between uWUE and VPD at the FLUXNET site IT-Col. Each box plot correspondes to 5% of the data. To aid visualization only the 0%-90% range of SWC bins are included.

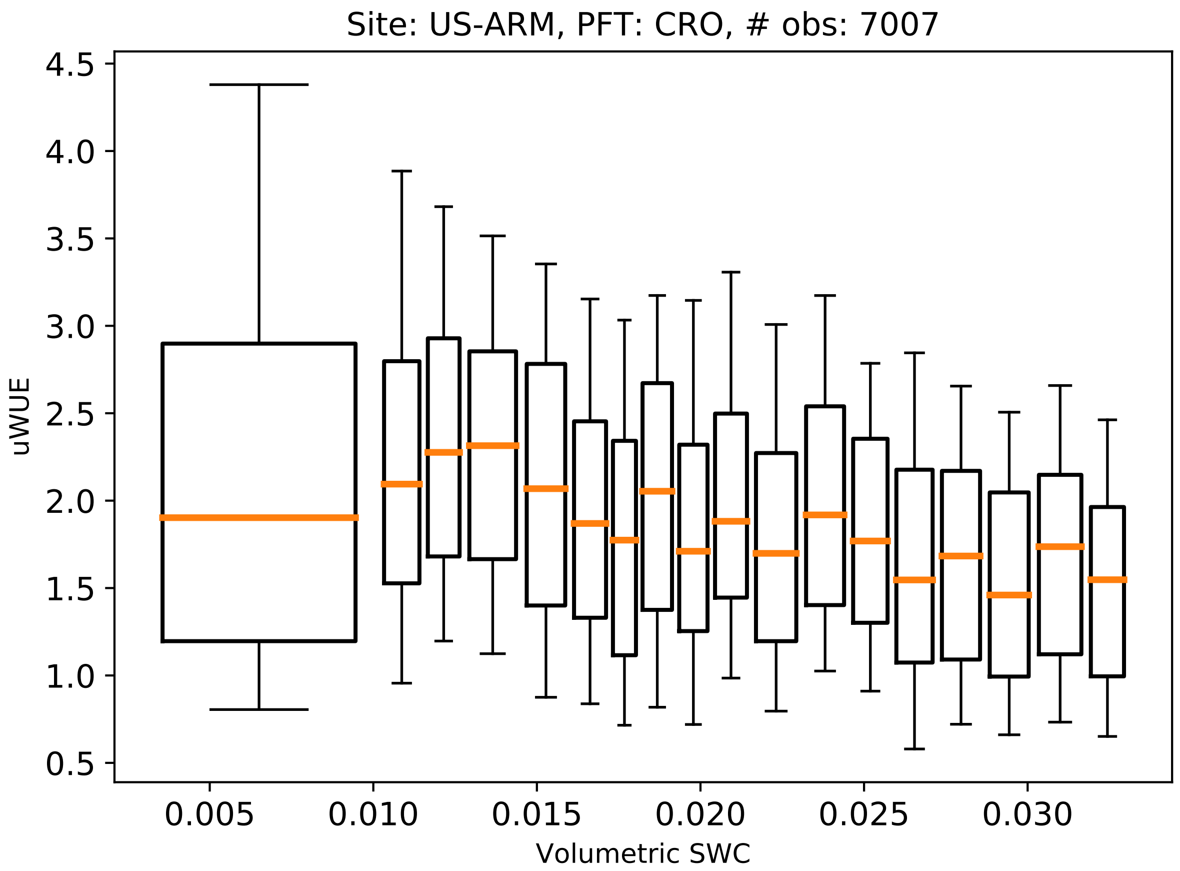

Figure S67. The relationship between uWUE and VPD at the FLUXNET site US-ARM. Each box plot correspondes to 5% of the data. To aid visualization only the 0%-90% range of SWC bins are included.

Table S1: Metadata and citations for flux sites used in this analysis. All data are gathered from `www.fluxdata.org`, and citations are aggregated using tools available at `https://github.com/trevorkeenan/FLUXNET_citations`.

Site	PFT	Lat	Lon	Clim[1]	Period	References
AT-Neu	GRA	47.1167	11.3175	Unk	2002-2012	Wohlfahrt et al. (2008)
AU-ASM	ENF	-22.2830	133.2490	Unk	2010-2013	Cleverly et al. (2013)
AU-Cpr	SAV	-34.0021	140.5891	Unk	2010-2014	Meyer et al. (2015)
AU-DaP	GRA	-14.0633	131.3181	Aw	2007-2013	Beringer et al. (2011)
AU-DaS	SAV	-14.1593	131.3881	Aw	2008-2014	Hutley et al. (2011)
AU-Dry	SAV	-15.2588	132.3706	Unk	2008-2014	Cernusak et al. (2011)
AU-Gin	WSA	-31.3764	115.7138	Unk	2011-2014	Beringer et al. (2016)
AU-How	WSA	-12.4943	131.1523	Aw	2001-2014	Beringer et al. (2007)
AU-Rig	GRA	-36.6499	145.5759	Unk	2011-2014	Beringer et al. (2016)
AU-Stp	GRA	-17.1507	133.3502	Unk	2008-2014	Beringer et al. (2011)
AU-Tum	EBF	-35.6566	148.1517	Cfb	2001-2014	Leuning et al. (2005)
AU-Whr	EBF	-36.6732	145.0294	Unk	2011-2014	McHugh et al. (2017)
AU-Wom	EBF	-37.4222	144.0944	Unk	2010-2012	Hinko-Najera et al. (2017)
BE-Lon	CRO	50.5516	4.7461	Cfb	2004-2014	Moureaux et al. (2006)
BE-Vie	MF	50.3051	5.9981	Cfb	1996-2014	Aubinet et al. (2001)
BR-Sa3	EBF	-3.0180	-54.9714	Am	2000-2004	Wick et al. (2005)
CA-Qfo	ENF	49.6925	-74.3421	Dfc	2003-2010	Bergeron et al. (2007)
CA-SF1	ENF	54.4850	-105.8176	Dfc	2003-2006	Mkhabela et al. (2009)
CA-SF2	ENF	54.2539	-105.8775	Dfc	2001-2005	Mkhabela et al. (2009)
CH-Cha	GRA	47.2102	8.4104	Unk	2005-2014	Merbold et al. (2014)
CH-Dav	ENF	46.8153	9.8559	Unk	1997-2014	Zielis et al. (2014)
CH-Fru	GRA	47.1158	8.5378	Unk	2005-2014	Imer et al. (2013)
DE-Geb	CRO	51.1001	10.9143	Unk	2001-2014	Anthoni et al. (2004)
DE-Gri	GRA	50.9500	13.5126	Cfb	2004-2014	Prescher et al. (2010)
DE-Hai	DBF	51.0792	10.4530	Unk	2000-2012	Knohl et al. (2003)
DE-Kli	CRO	50.8931	13.5224	Cfb	2004-2014	Prescher et al. (2010)
DE-Lkb	ENF	49.0996	13.3047	Unk	2009-2013	Lindauer et al. (2014)
DE-Obe	ENF	50.7867	13.7213	Cfb	2008-2014	–
DE-Seh	CRO	50.8706	6.4497	Unk	2007-2010	Schmidt et al. (2012)
DE-Tha	ENF	50.9624	13.5652	Cfb	1996-2014	Grünwald & Bernhofer (2007)
DK-Sor	DBF	55.4859	11.6446	Unk	1996-2014	Pilegaard et al. (2011)
FI-Hyy	ENF	61.8474	24.2948	Unk	1996-2014	Suni et al. (2003)
FI-Sod	ENF	67.3619	26.6378	Unk	2001-2014	Thum et al. (2007)
FR-Gri	CRO	48.8442	1.9519	Cfb	2004-2013	Loubet et al. (2011)
FR-LBr	ENF	44.7171	-0.7693	Unk	1996-2008	Berbigier et al. (2001)
IT-Col	DBF	41.8494	13.5881	Unk	1996-2014	Valentini et al. (1996)
IT-Cpz	EBF	41.7052	12.3761	Unk	1997-2009	Garbulsky et al. (2008)
IT-Lav	ENF	45.9562	11.2813	Unk	2003-2014	Marcolla et al. (2003)
IT-MBo	GRA	46.0147	11.0458	Unk	2003-2013	Marcolla et al. (2011)

IT-Noe	CSH	40.6061	8.1515	Unk	2004-2014	Papale et al. (2014)
IT-Ren	ENF	46.5869	11.4337	Unk	1998-2013	Montagnani et al. (2009)
IT-Ro2	DBF	42.3903	11.9209	Unk	2002-2012	Tedeschi et al. (2006)
IT-SRo	ENF	43.7279	10.2844	Unk	1999-2012	Chiesi et al. (2005)
IT-Tor	GRA	45.8444	7.5781	Unk	2008-2014	Galvagno et al. (2013)
NL-Loo	ENF	52.1666	5.7436	Unk	1996-2013	Moors (2012)
RU-Fyo	ENF	56.4615	32.9221	Unk	1998-2014	Kurbatova et al. (2008)
US-AR1	GRA	36.4267	-99.4200	Dsa	2009-2012	Raz-Yaseef et al. (2015)
US-AR2	GRA	36.6358	-99.5975	Dsa	2009-2012	Raz-Yaseef et al. (2015)
US-ARM	CRO	36.6058	-97.4888	Cfa	2003-2012	Fischer et al. (2007)
US-Blo	ENF	38.8953	-120.6328	Csa	1997-2007	Goldstein et al. (2000)
US-KS2	CSH	28.6086	-80.6715	Cwa	2003-2006	Powell et al. (2006)
US-MMS	DBF	39.3232	-86.4131	Cfa	1999-2014	Dragoni et al. (2011)
US-Me2	ENF	44.4523	-121.5574	Csb	2002-2014	Irvine et al. (2008)
US-NR1	ENF	40.0329	-105.5464	Dfc	1998-2014	Monson et al. (2002)
US-Ne1	CRO	41.1651	-96.4766	Dfa	2001-2013	Verma et al. (2005)
US-Ne2	CRO	41.1649	-96.4701	Dfa	2001-2013	Verma et al. (2005)
US-Ne3	CRO	41.1797	-96.4397	Dfa	2001-2013	Verma et al. (2005)
US-SRG	GRA	31.7894	-110.8277	Bsk	2008-2014	Scott et al. (2015)
US-SRM	WSA	31.8214	-110.8661	Bsk	2004-2014	Scott et al. (2009)
US-Syv	MF	46.2420	-89.3477	Dfb	2001-2014	Desai et al. (2005)
US-Ton	WSA	38.4316	-120.9660	Csa	2001-2014	Baldocchi et al. (2010)
US-Var	GRA	38.4133	-120.9507	Csa	2000-2014	Ma et al. (2007)
US-WCr	DBF	45.8059	-90.0799	Dfb	1999-2014	Cook et al. (2004)
US-Wkg	GRA	31.7365	-109.9419	Bsk	2004-2014	Scott et al. (2010)
ZA-Kru	SAV	-25.0197	31.4969	Unk	2000-2010	Archibald et al. (2009)
ZM-Mon	DBF	-15.4378	23.2528	Unk	2000-2009	Merbold et al. (2009)

[1] Köppen Climate classification.
